# Adverse Childhood Experiences and Trauma-Informed Care: An Online Module for Pediatricians

**DOI:** 10.15766/mep_2374-8265.10851

**Published:** 2019-11-01

**Authors:** Anna Schmitz, Susan Light, Courtney Barry, Kelly Hodges

**Affiliations:** 1 Assistant Professor, Department of Pediatrics, Medical College of Wisconsin; 2 Pediatrician, Children's Medical Group; 3 Assistant Professor, Department of Psychiatry and Behavioral Medicine, Medical College of Wisconsin; 4 Assistant Professor, Department of Family and Community Medicine, Medical College of Wisconsin; 5 Associate Professor, Department of Pediatrics, Medical College of Wisconsin

**Keywords:** Adverse Childhood Experiences, Trauma-Informed Care, Resident Education, Pediatrics, Preventive Medicine

## Abstract

**Introduction:**

The epidemic of adverse childhood experiences (ACEs) has many known health consequences. Robust research has linked ACEs to increased morbidity and mortality. Because of their frequent interaction with children and their families, pediatricians should be educated to recognize ACEs and practice trauma-informed care (TIC). There is a lack of education for pediatric residents on ACEs despite their significance. Our goals were to identify residents’ baseline perceived importance, confidence, and frequency of discussion of ACEs, TIC, toxic stress, and resiliency and evaluate the efficacy of an educational module addressing these topics.

**Methods:**

A 25-minute self-directed module was created for pediatric residents. The module was accessible online and independently completed by residents during the child advocacy rotation. Pre- and postmodule surveys using a 5-point Likert scale (1 = *low*, 5 = *high*) were administered, and median scores of 11 participants who completed both surveys were compared using the Wilcoxon signed rank test.

**Results:**

Presurvey results demonstrated that residents were not confident discussing ACEs, TIC, or resiliency (median = 2). Residents reported that it was very important to discuss ACEs, toxic stress, and resiliency with families (median = 5), although they were rarely discussed in clinic (median = 1 or 2). Matched pre/post data showed significant increases in knowledge, confidence, and discussion frequency.

**Discussion:**

The results demonstrated a need for ACE education for pediatric residents. The matched survey results showed the module's success in knowledge and behavior change. The module can be adapted to other learners to enhance understanding of ACEs.

## Educational Objectives

By the end of this activity, learners will be able to:

1. Explain the science behind the effects of adverse childhood experiences (ACEs) and toxic stress on the health and development of children and the well-being of their families.
2. List the ways that ACEs and childhood toxic stress impact the health and development of children.
3. Give examples of how a pediatrician could initiate a conversation about previous trauma with a parent or patient.
4. Recognize the interactions and behaviors of patients and families who have been affected by toxic stress.
5. Describe approaches to helping parents and children who have been affected by toxic stress.

## Introduction

The epidemic of adverse childhood experiences (ACEs) is a public health crisis causing short- and long-term negative health outcomes in children, families, and communities. ACEs are stressors that can impact child development and health outcomes for adults. Stress can induce both psychological and physiologic responses within the body. Physiologic responses to stress include activation of the hypothalamic-pituitary-adrenocortical axis and the sympathetic-adrenomedullary systems, which results in the increase of stress hormones.^1^ Chronic stress exposure and release of stress hormones can lead to deterioration in the body.^1^ Exposure to stress is unavoidable, but the impact on the body and mind changes depending on the type of stress and subsequent response. There are three different types of stress responses that the body can experience: positive, tolerable, and toxic.^1^ A positive stress response occurs when the body returns to baseline levels of stress hormones relatively quickly and the experience is considered mild to moderate. Examples of positive stress range from giving an oral presentation at school to a minor car accident; these experiences can help a person learn and grow. A tolerable stress can happen when events occur unexpectedly, such as the loss of a family member, but there are supportive individuals or communities who help reduce the stress response.^1^ Toxic stress is the term used for experiences or stressors that ultimately have lasting negative effects on cognitive, social, emotional, or neurobiological development. Examples of biological changes from toxic stress include changes in neuronal development with overactivation of the fight-or-flight stress response and underdevelopment of other areas of the brain involved in executive function.^1^ ACEs can lead to toxic stress in the right environment and are prevalent in the United States.

A survey of 4,023 youth, 12-17 years of age, found that 17.4% experienced physical abuse, 8.1% experienced sexual abuse, and 39.4% witnessed interpersonal violence.^2^ The original study on the prevalence of ACEs illustrated that more than half of the 9,508 adults who responded reported at least one ACE and that one-fourth reported two or more ACEs.^3^ The study demonstrated that those who experienced four or more ACEs, compared to those with none, had four- to 12-fold increased risk for the following: alcoholism, drug abuse, depression, and suicide attempt. In examining the relationship of ACEs and chronic adulthood diseases, a graded dose-response relationship was found between the number of ACEs to the presence of adult diseases including ischemic heart disease, cancer, chronic lung disease, skeletal fractures, and liver disease.^3^ Specifically, the more ACEs experienced during childhood, the more likely one would be to develop a chronic health condition in adulthood.^3^ Patients with a history of ACEs often have developed certain behavioral coping mechanisms to help them manage thoughts and feelings that arise from their past adversity.^2,4^ Some of these harmful adaptive coping mechanisms, such as substance use or overeating, can make it difficult for a provider to facilitate behavioral change if the trauma is not addressed.^5^ Moreover, these behaviors are not specific for a trauma history and can be subtle or mimic the symptoms of other conditions. It is vital that health care providers understand the prevalence and impact of ACEs on health and development to provide optimal care to patients and families. In fact, individuals with trauma may be more likely to turn to their health care provider, due to the established and trusting relationship.^4^ Considering that patients and families may not always connect their trauma to certain health behaviors or be forthcoming about past adversity, it is important for providers to inquire about trauma to make appropriate diagnoses and recommendations for treatment. Pediatricians can play a vital role in a coordinated effort to promote healthy childhood development on both an individual and community level. The pediatrician can work with community organizations to minimize and mitigate stressors that are identified during clinical care.^6^ In addition to medical services, pediatricians can assist with developing evidence-based programs for children and families and collaborate with political leaders to impact policy changes.^7^

Providing care for patients and families while being mindful of potential past adversities and how these adversities can affect their decisions today is the art of practicing trauma-informed care (TIC). The Substance Abuse and Mental Health Services Administration discusses a TIC approach that entails realizing the impact of trauma, recognizing the symptoms, and responding to the patient by integrating knowledge about trauma into practices while not retraumatizing patients.^8^ This approach has the potential to strengthen the patient-provider relationship and, in turn, positively impact physical and mental health outcomes for patients.

Although the significance of ACEs and TIC on health and child development is well studied, a structured curriculum on these topics is not available to pediatric medical residents within their training program. Many residents intuitively recognize and understand the significance of ACEs, but they often struggle with how to incorporate this knowledge into practice. There is uncertainty about how to counsel and care for patients who have previously experienced adversity. Residents rely on supervising physicians to teach them all aspects of medicine, including patient-family interactions. The type and amount of education that addresses ACEs and TIC are variable and often minimal within residency curricula. The American Board of Pediatrics delineates expectations for pediatric residents to meet certain milestones during training. ACE and TIC education is integral to achieving competency in multiple areas, most notably in professionalism and interpersonal and communication skills. A few examples include interpersonal and communication skills competency numbers 1 and 2: “Communicate effectively with patients, families, and the public, as appropriate, across a broad range of socioeconomic and cultural backgrounds” and “Demonstrate the insight and understanding into emotion and human response to emotion that allow one to appropriately develop and manage human interactions.”^9^ We sought to standardize education on these important topics, as the evidence is relatively new and evolving. It has become increasingly more difficult to get residents and educators together for lectures or small-group discussions due to scheduling conflicts and clinical demands. For these reasons, an online module was chosen to disseminate this information to make it readily accessible and sustainable, without requiring the presence of specific educators.

A review of the trauma literature revealed few training modules on trauma for medical professionals.^10^ A search in *MedEdPORTAL* using the term *adverse childhood experiences* yielded several results. One publication was a curriculum for medical students on the topic of ACEs, TIC, and resiliency.^11^ The results showed that the module, along with a facilitated case discussion in small groups, was an effective way to teach students about ACEs.^11^ The authors found that one area identified by the students for future improvement would be more information on how to incorporate ACEs into clinical care.^11^ This is an important aspect that our module was designed to address. Another module, also for medical students and faculty, focused on teaching physical examination skills in a trauma-informed approach.^12^ The results revealed that the module led to an increase in knowledge of trauma-informed language and physical examination maneuvers.^12^ The results of these publications support the use of modules as an effective way to teach concepts related to ACEs and TIC. Ours is the first module on ACEs and TIC that is designed for pediatric residents, peer-reviewed, and available online. Our online module can help achieve the following goals: (1) to define ACEs and describe their influences on pediatric patients and their families and (2) to provide health care providers with useful clinical tools to recognize patients at risk for the negative impacts of ACEs and approach their care in a way that is mindful of their unique history and needs.

## Methods

The project team included a small group of pediatric residents and several child abuse and neglect (CAN) physicians. The team designed a project with the following aims as related to ACEs, TIC, toxic stress, and resiliency: (1) assess pediatric residents’ perceived importance of these topics, (2) increase knowledge of these topics, (3) change residents’ behaviors/practices related to these topics, and (4) influence the residency program's overall approach to these topics. In addition, the team established the primary goals for the development of the educational tool: It should be effective, accurate, succinct, and easily accessible to learners and should provide practical information for current and future practice. The team also planned to have the learning tool incorporated into the residency curriculum, requiring that it be sustainable over time without the presence of specific instructors and independent of scheduling conflicts or time constraints. With these considerations, as well as the needs of the learners, in mind, the module was designed to be self-guided, easily accessible, and available online.

Two pediatric residents and CAN physicians attended an ACE master training session, designed to train the trainer regarding ACEs, TIC, and resiliency. The training session was an interactive 2-day lecture series that used a licensed PowerPoint presentation created by pioneer ACE researchers Dr. Robert Anda and Laura Porter. It provided background knowledge, emphasized the importance, and encouraged further dissemination of the aforementioned topics. The two pediatric residents utilized the knowledge gained at the training session and completed a literature review on these topics to develop an outline for an online, educational, and interactive module (Appendix A). The residents created the online module and subsequently had key stakeholders edit and revise the module for accuracy. The stakeholders were identified as people with unique perspectives to provide feedback on the module and included pediatric residents, CAN physicians, adult education specialists, and an experienced ACE master trainer. To maximize interactive learning activities in the module, Captivate software (Adobe, San Jose, California) was used for primary module creation, with a similar PowerPoint version also created to allow for distribution of the module for those who did not have access to Captivate. The PowerPoint version is included as Appendix A. The Captivate version was disseminated to residents through an online education website, Desire to Learn (D2L), which was supported in the residency program.

Once the module was made and available online, no further setup or teaching time was required from faculty. The module link was provided to residents at the beginning of their 4-week required rotation in child advocacy and protection (CAP) during the second year of pediatric residency. The module took residents an average of 25 minutes to complete. Residents could complete the module online through the D2L website during any free time. The module was not mandatory, but protected time was arranged and computer space provided during the rotation to facilitate higher rates of module completion. The module was designed to be completed individually and could potentially be followed up with small-group discussion of the topics. During the CAP rotation, residents worked closely with CAN faculty and the topics of ACEs and TIC were frequently discussed during clinical care. In other settings, the module could be completed prior to small-group discussions or as an adjunct to large-group lectures. If the module is used as a lecture guide or part of a small-group discussion, we estimate that it would take at least an hour to go through the material.

To gather baseline data, the project team developed an online, computer-based survey (Appendix B) to assess knowledge and comfort in discussing ACEs, TIC, toxic stress, and resiliency, as well as confidence in and frequency of incorporating the information into clinical practice. This survey utilized an online computer-based program (SurveyMonkey), which allowed for easy completion with internet access. Survey questions were developed and utilized such that subsequent postmodule survey data could be directly compared in measuring the reaction to the module and the effects on learning and behavior, in accordance with the Kirkpatrick model. The survey examined the general reaction to the module by inquiring if learners thought the module was effective and how likely they would be to recommend the module to others. To assess the learning and knowledge gained from the module, the participants rated their knowledge on the topics (ACEs, resiliency, TIC, toxic stress) on a 5-point Likert scale (1 = *low*, 3 = *neutral*, 5 = *high*). To assess behavior changes, the surveys asked for a rating of frequency of discussion of these topics during a regular outpatient clinic visit on a 5-point Likert scale (1 = *no visits*, 5 = *all visits*). To improve completion rates, the survey was linked to the online module, and opening the survey was required prior to beginning the module. The premodule survey results are described as median values (*n* = 29).

The residents received a postmodule survey 1-3 months following their CAP rotation (Appendix C). This time frame was chosen to balance the response rate and determine if any behavior changes were established and sustained. This survey assessed the efficacy of the module in improving knowledge of, confidence in, and frequency of discussion of the topics. To protect anonymity and allow for matched results, the pre- and postmodule surveys contained unique identifiers. The survey results were analyzed using SPSS software (IBM, Armonk, New York). The Likert-scale answers were tested for normality utilizing the Kurtosis method and found to be not normally distributed. To compare the pre- and postmodule survey median scores of participants who completed both surveys (*n* = 11), researchers used the nonparametric Wilcoxon signed rank exact test. The postmodule surveys were sent to residents who completed the module through July 2018.

The project, including the surveys, was approved by the Medical College of Wisconsin Institutional Review Board (IRB) on February 15, 2016. In accordance with IRB approval, survey and module completion were voluntary. The number and timing of email reminders for survey completion were also regulated.

## Results

**Figure 1.**
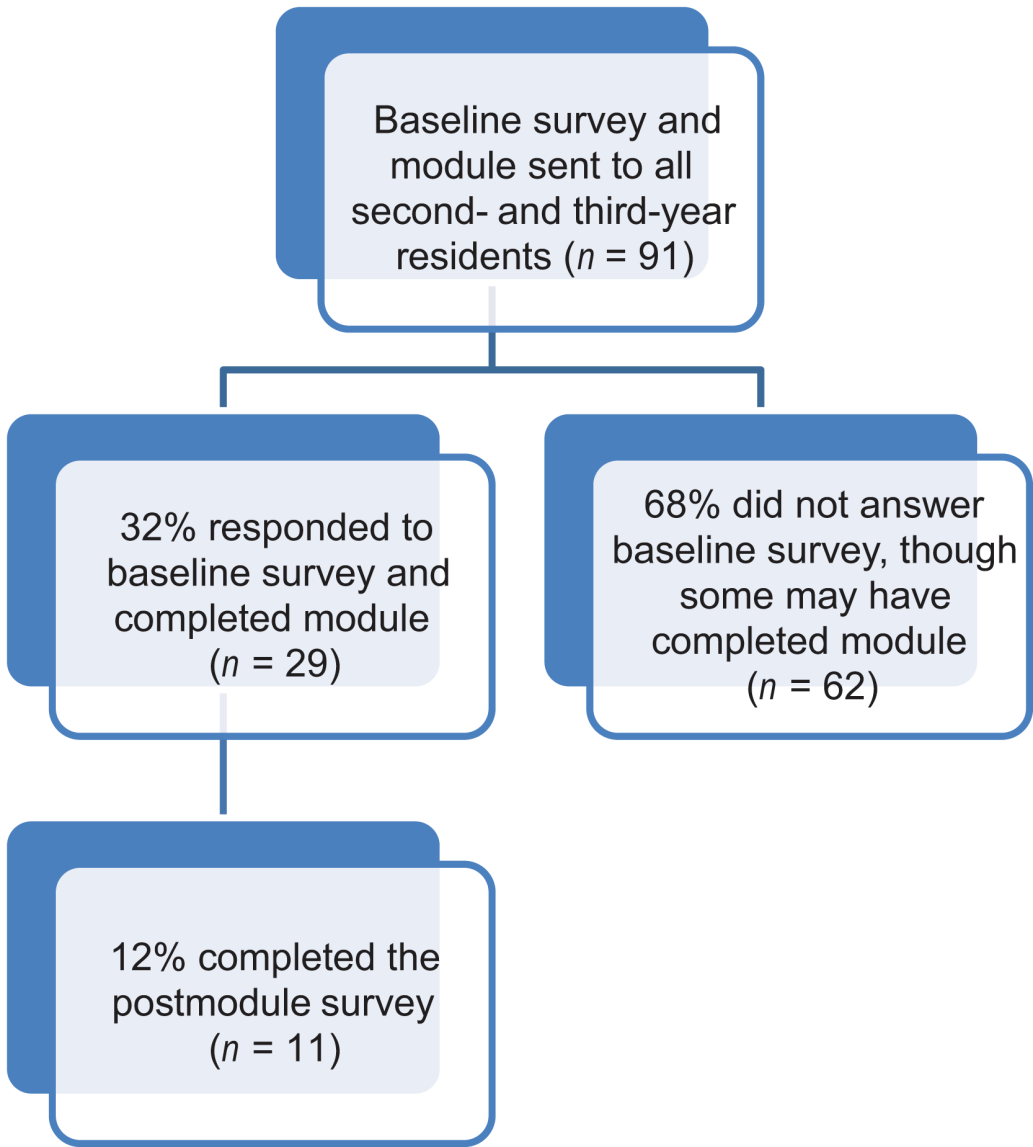
Breakdown of residents who completed the baseline survey, module, and postmodule survey.

The survey and module were distributed to a total of 91 pediatric residents over the course of 19 months in 2016-2017. In the first month, the survey and module were sent to all third-year pediatric and medicine-pediatric residents, as these residents had already completed their required 4-week CAP rotation. In the subsequent months, second-year pediatric and medicine-pediatric residents were provided with the survey and module link during the orientation for their required CAP rotation. We were not able to assess how many of these residents completed the module, although we expect that it was viewed at a higher rate than our survey response rate based on verbal feedback from many residents who viewed the module but declined to answer the baseline survey. There were 29 residents who responded to the baseline survey, for a response rate of 32%. The postmodule survey sent out 1-3 months after the CAP rotation was completed by 11 residents, for a response rate of 12%. Figure 1 presents a breakdown of resident participants. Since the study period, the module has remained a part of the CAP rotation; thus, each resident graduating from the program is exposed to the module.

In the surveys, questions were asked about confidence, importance, and frequency of discussion in certain topics. It should be noted that TIC is not something discussed but rather practiced. This should be clarified on future surveys. The results described in this section reflect the original wording in the survey. No personal information was obtained from survey respondents. One question asked about the type of continuity clinic in which the resident participated. The majority of respondents participated in a suburban private practice with more than 50% Medicaid patients (*n* = 15, 52%), followed by a federally qualified health center (*n* = 9, 31%) and a suburban private practice with less than 50% Medicaid patients (*n* = 2, 7%). Three people did not answer the question. This question was included to evaluate if there were differences in how residents responded to or incorporated these topics into practice based on where they spent the majority of their clinic time. With the low response rate in the survey, no significant conclusions could be drawn from this information.

**Figure 2.**
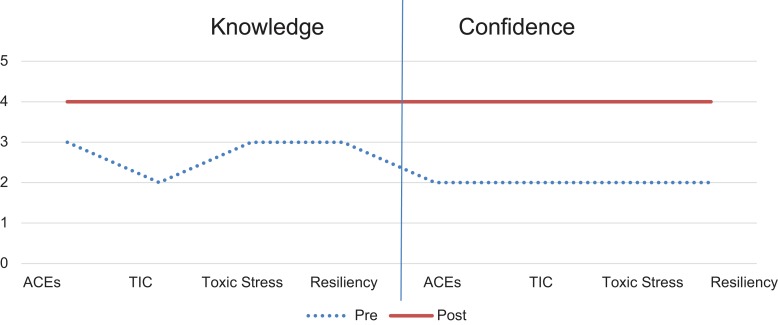
Median rating of knowledge and confidence of topics in the premodule (*n* = 29) and postmodule (*n* = 11) surveys. For matched pairs, *p* < .05. ACEs, adverse childhood experiences; TIC, trauma-informed care.

In the baseline survey, residents felt it was important to discuss ACEs (median = 5 [very important]), TIC (median = 4), toxic stress (median = 5), and resiliency (median = 5) with patients and families. Although residents felt it was important to address ACEs, many residents did not feel confident in discussing ACEs, TIC, or resiliency with patients and families (median = 2). Results from matched pre- and postmodule surveys demonstrated an increased confidence in knowledge of ACEs (from 3 to 4, *p* < .05), TIC (from 2 to 4, *p* < .05), toxic stress (from 2 to 4, *p* < .05), and resiliency (from 3 to 4, *p* < .05). Confidence in discussing all topics (ACEs, TIC, toxic stress, and resiliency) increased significantly from a median of 2 premodule to a median of 4 postmodule (*p* < .05). Figure 2 provides a graphic representation of pre- and postmodule survey results. The importance of discussing ACEs, TIC, toxic stress, and resiliency was rated as important or very important on both the pre- and postmodule surveys.

**Figure 3.**
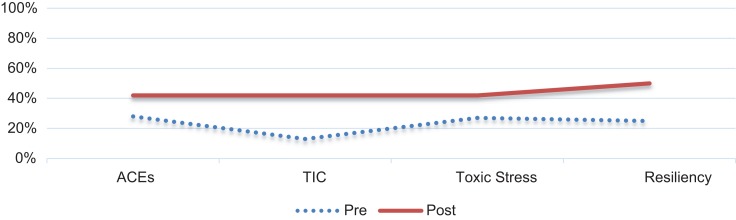
Percentage of residents who reported discussing topics at some or most visits with the families premodule (*n* = 29) and postmodule (*n* = 11). For all matched pairs, *p* < .01. ACEs, adverse childhood experiences; TIC, trauma-informed care.

In addition to increasing knowledge and confidence in discussing the topics with families, the module was assessed for its ability to impact behavior change. The residents self-reported increased frequency of discussion of all topics in the postmodule survey. The percentage of residents who reported discussing these topics at some or most visits with families increased following module completion. Discussion of ACEs increased from 28% to 42%, TIC discussion increased from 13% to 42%, toxic stress discussion increased from 27% to 42%, and resiliency discussion increased from 25% to 50% (*p* < .01 for all matched pairs). Figure 3 presents a graphical comparison. As a balancing measure, residents were asked how long a typical clinic visit took (in minutes). The responses in the pre- and postmodule surveys were the same, which helps support the idea that incorporating these topics into clinic visits does not significantly lengthen the encounter.

All residents who responded reported the module to be an effective means of teaching these concepts and would recommend the module to others (only four answered this question; all others left the answer blank). Comments on the postmodule survey included the following: “The module has made me more aware of how ACEs impact my patients,” “I do consider a patient's background more before clinical encounters and at least feel a little more empathetic,” and “The training made me more aware of these issues and how they impact patients and their families.”

## Discussion

The literature demonstrates the importance of understanding ACEs and their relationship to the development of chronic health conditions. However, there is not a standardized curriculum to educate pediatric residents on ACEs and TIC. Some medical schools are in the early stages of incorporating this education into training, but most residents have little to no exposure to these topics prior to residency. This gap in training and knowledge led to the creation of a module focusing on ACEs and TIC education for pediatric residents. The baseline survey results emphasized that pediatric residents found it important to address ACEs, TIC, toxic stress, and resiliency when interacting with families, yet they did not feel confident in doing so. After being introduced to these topics and provided with tools on how to address them with families, pediatric residents significantly increased confidence in their knowledge and ability to discuss or practice the topics. Perhaps most importantly, residents demonstrated behavioral changes as they reported more frequently addressing ACEs, TIC, toxic stress, and resiliency within a clinical encounter.

The module functions to ensure that core elements are conveyed to all learners. It has the potential to be utilized in a variety of settings, including individual use, as preparation for a lecture or small-group discussion, as the core of a lecture or presentation to a group, or as a template for the development of a curriculum on these topics. In an effort to keep the module succinct and focused on ACEs and TIC, some related important topics were not covered in detail. Confidentiality and documentation of sensitive materials in the medical record were not stressed in the module, as they were addressed at other times during our pediatric residency training. Similarly, although mandatory reporting principles were included in the module, specific guidelines were not included, as our residents received separate lectures and training on them and more detail was not within the scope of this module. For other institutions, these may be important topics to consider covering more thoroughly with ACE and TIC teaching.

The research team noted several limitations in the development and implementation of this module and survey design. The survey asked residents about the frequency of discussion of TIC; however, in actuality, TIC is something that is practiced, and trauma experiences or symptoms are discussed. The survey wording should be clarified if it is to be used again. Residents are interested in learning about these topics, although barriers to gaining this knowledge include limited time during medical training for additional topics and the difficulty in completing an optional module during their free time. To address these barriers, the project team embedded the module into an already established rotation, and the rotation provided protected time to complete the module. To allow for accessibility, the Captivate module distribution was done through D2L, an online interface with which residents were already familiar. Captivate requires a specific website platform for viewing and purchased software for editing; for these reasons, it is not included in this publication. For those who may not have D2L or a similar website with Captivate capabilities, the PowerPoint presentation format is a more widely accepted mode of accessing the module content. A disadvantage of utilizing the PowerPoint version is that the interactive and aesthetic features are not as robust; however, the content of the module remains consistent in both software platforms. In future use, the PowerPoint version may be enhanced through combination with a small-group activity or a voice-over facilitator guide to highlight certain points.

Another limitation identified was completion of the surveys. To allow for a greater sample size, premodule survey completion was facilitated by requiring opening of the survey to access the module. Postmodule survey completion rate was low, which made gathering enough paired samples to produce statistically significant results more difficult. The limited postmodule survey response rate is likely due to that survey not being mandatory to complete. The initial results with 11 paired samples show the module's success, although more responses could show greater impact. Going forward, small-group in-person discussions either before or after the module may increase module participation and likely increase survey response rates. Additional opportunities for the growth of this content exist in adjusting the mode of delivery to learners, whether it be through module participation as a group activity or modification for use as a lecture.

The survey responses demonstrate a need for pediatric residents to receive TIC and ACE education. To further address ACEs and TIC, the Medical College of Wisconsin Department of Pediatrics has formed partnerships to begin a system-wide approach to TIC. This may be an opportunity for the module to be adapted for more widespread distribution. Due to the lack of curricula and modules available for residency training on ACEs, TIC, toxic stress, and resiliency, we foresee medical schools, residency programs, and hospital systems looking for ways to provide awareness and education on these important topics. This module can be utilized to start this important conversation, as it is designed not only to provide education but also to empower residents to feel more comfortable having these discussions in everyday practice. The module can easily be adapted to other training programs, including medical or other professional health care schools, to increase and augment knowledge, awareness, and confidence in these topics. With further education on ACEs and TIC, we can begin to disrupt the cycle of trauma and its effect on chronic health conditions.

## Appendices

A. ACEs PowerPoint.pptx
B. ACEs Premodule Survey.docx
C. ACEs Postmodule Survey.docx All appendices are peer reviewed as integral parts of the Original Publication.
