# Supplementary material for: Adverse Childhood Experiences and Trauma-Informed Care: An Online Module for Pediatricians
Source: MedEdPORTAL. 2019 Nov 1;15:10851. doi: 10.15766/mep_2374-8265.10851 (PMC6952282; doi:10.15766/mep_2374-8265.10851)
Supplement: Supplementary file 1 — A. ACEs PowerPoint.pptx B. ACEs Premodule Survey.docx C. ACEs Postmodule Survey.docx [file mep-15-10851-s001.zip › A. ACEs PowerPoint.pptx]

## Slide 1
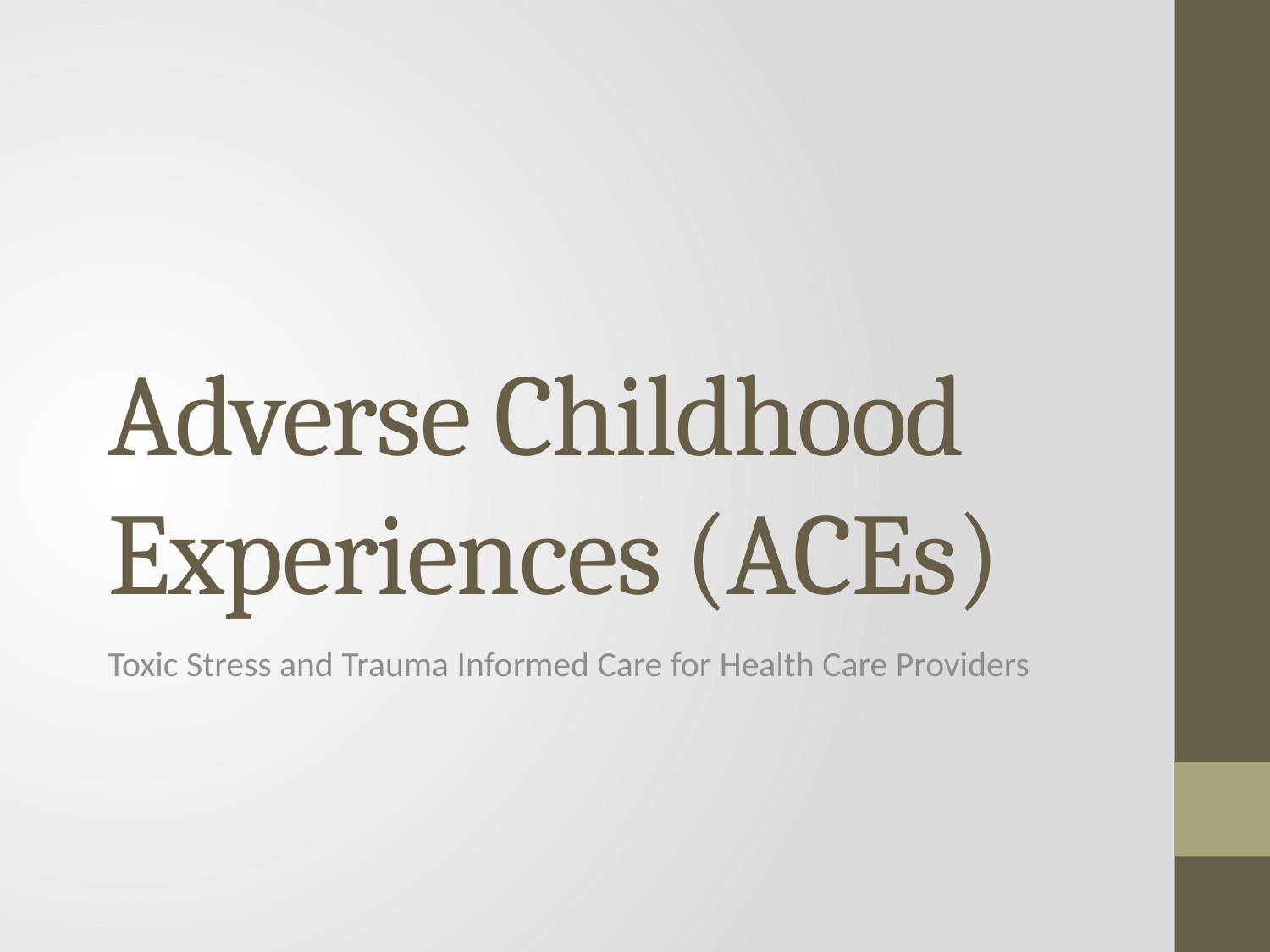

# Adverse Childhood Experiences (ACEs)
Toxic Stress and Trauma Informed Care for Health Care Providers

## Slide 2
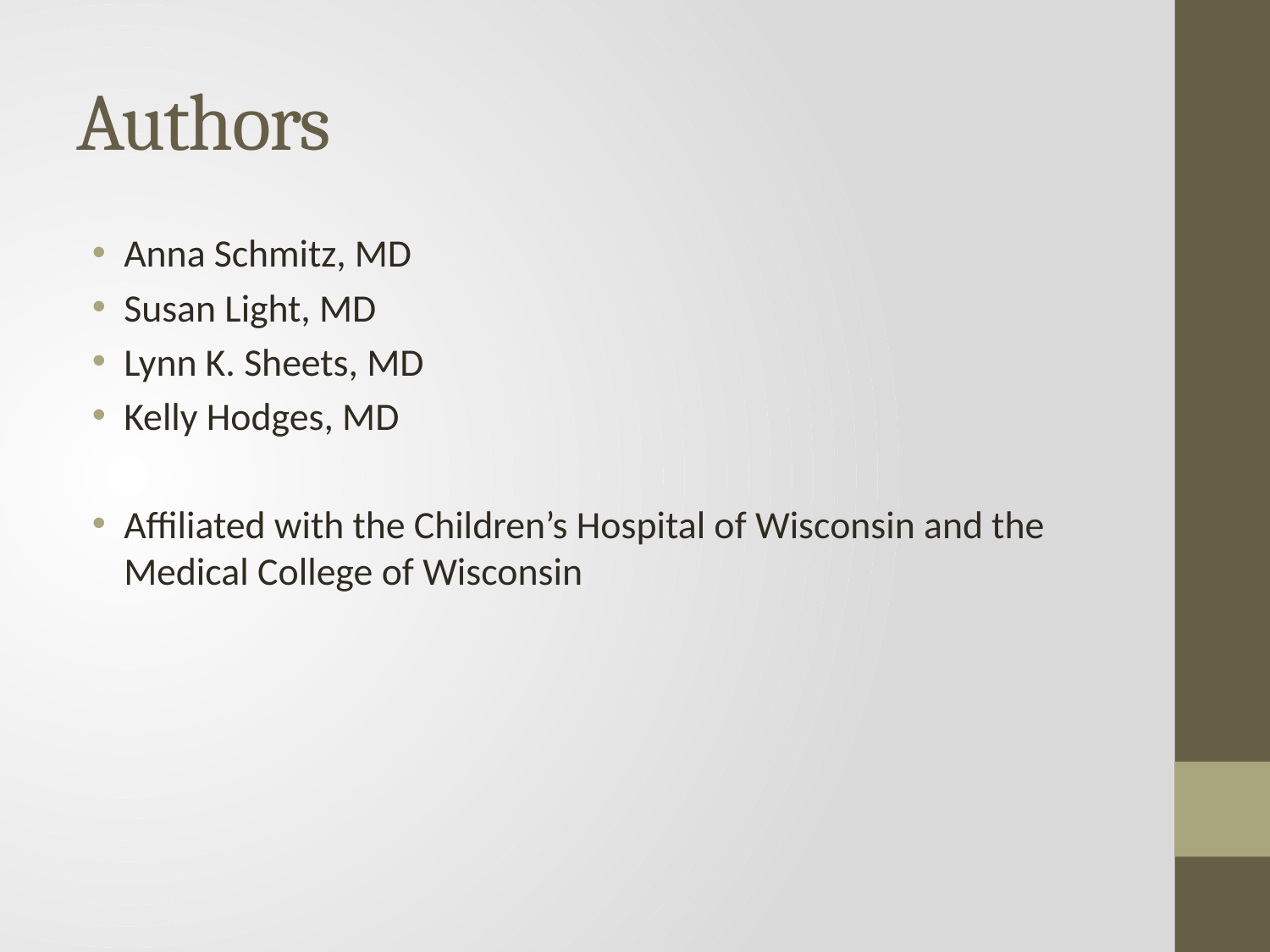

# Authors
Anna Schmitz, MD
Susan Light, MD
Lynn K. Sheets, MD
Kelly Hodges, MD
Affiliated with the Children’s Hospital of Wisconsin and the Medical College of Wisconsin

## Slide 3
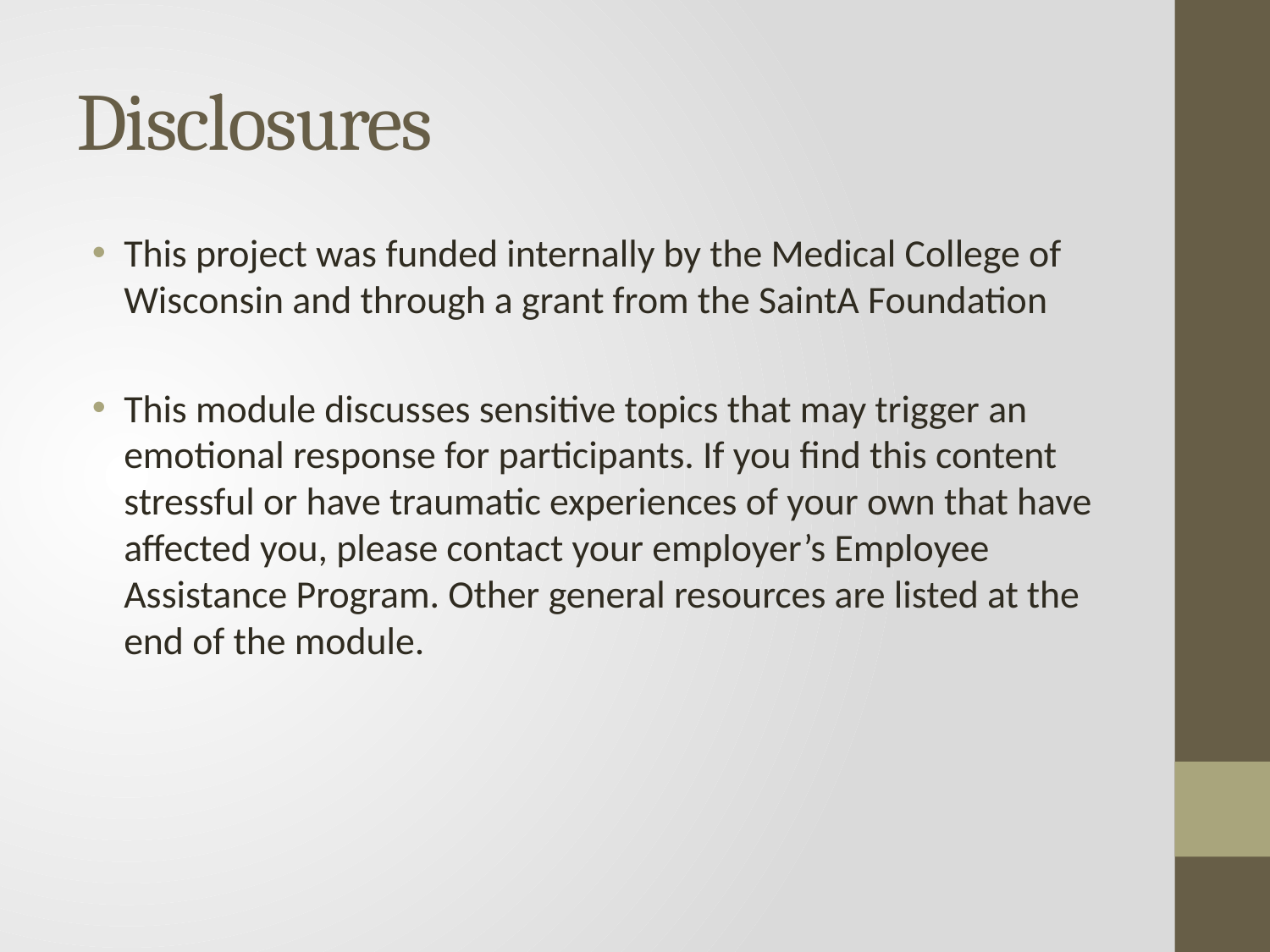

# Disclosures
This project was funded internally by the Medical College of Wisconsin and through a grant from the SaintA Foundation
This module discusses sensitive topics that may trigger an emotional response for participants. If you find this content stressful or have traumatic experiences of your own that have affected you, please contact your employer’s Employee Assistance Program. Other general resources are listed at the end of the module.

## Slide 4
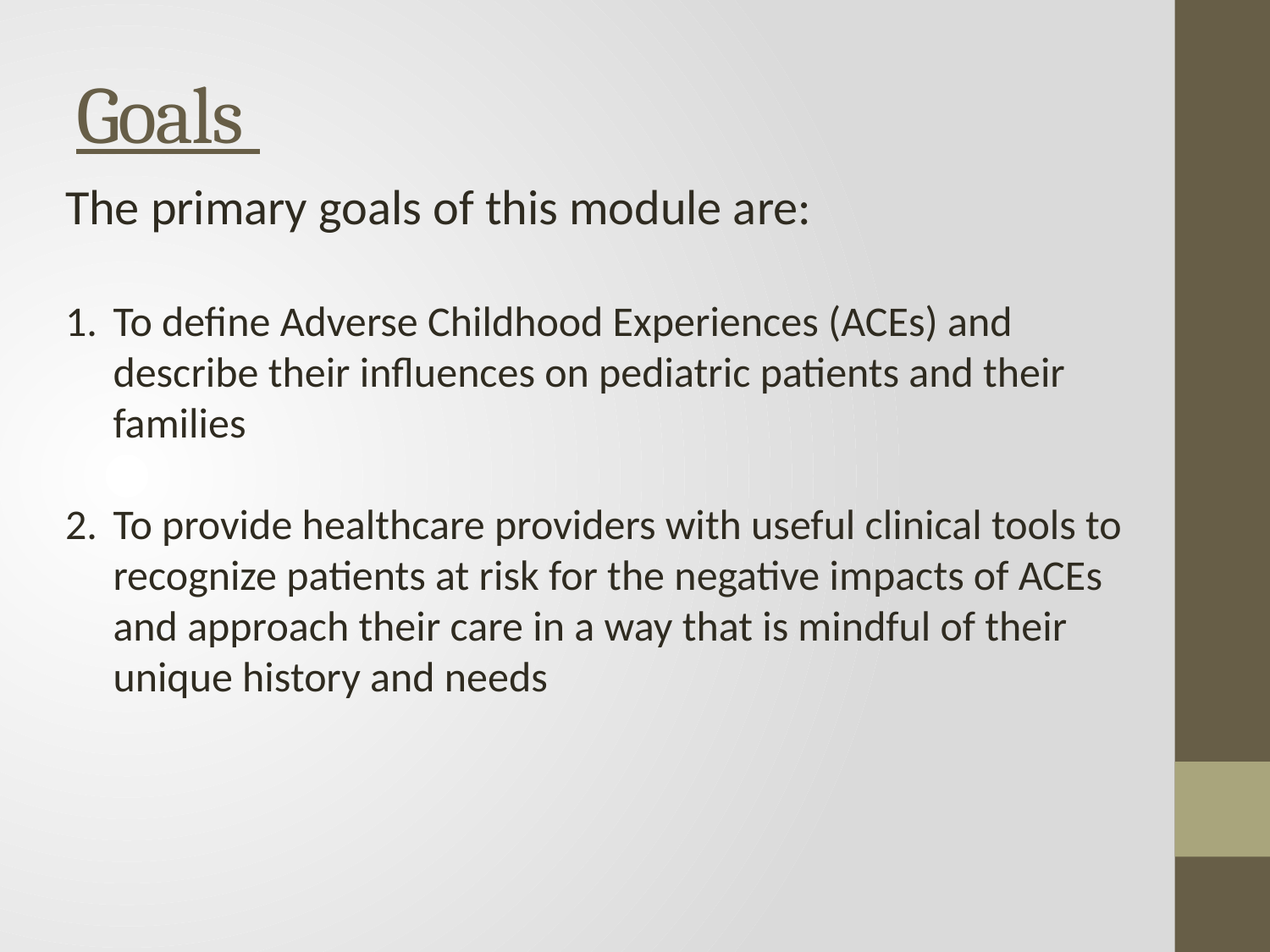

# Goals
The primary goals of this module are:
To define Adverse Childhood Experiences (ACEs) and describe their influences on pediatric patients and their families
To provide healthcare providers with useful clinical tools to recognize patients at risk for the negative impacts of ACEs and approach their care in a way that is mindful of their unique history and needs

## Slide 5
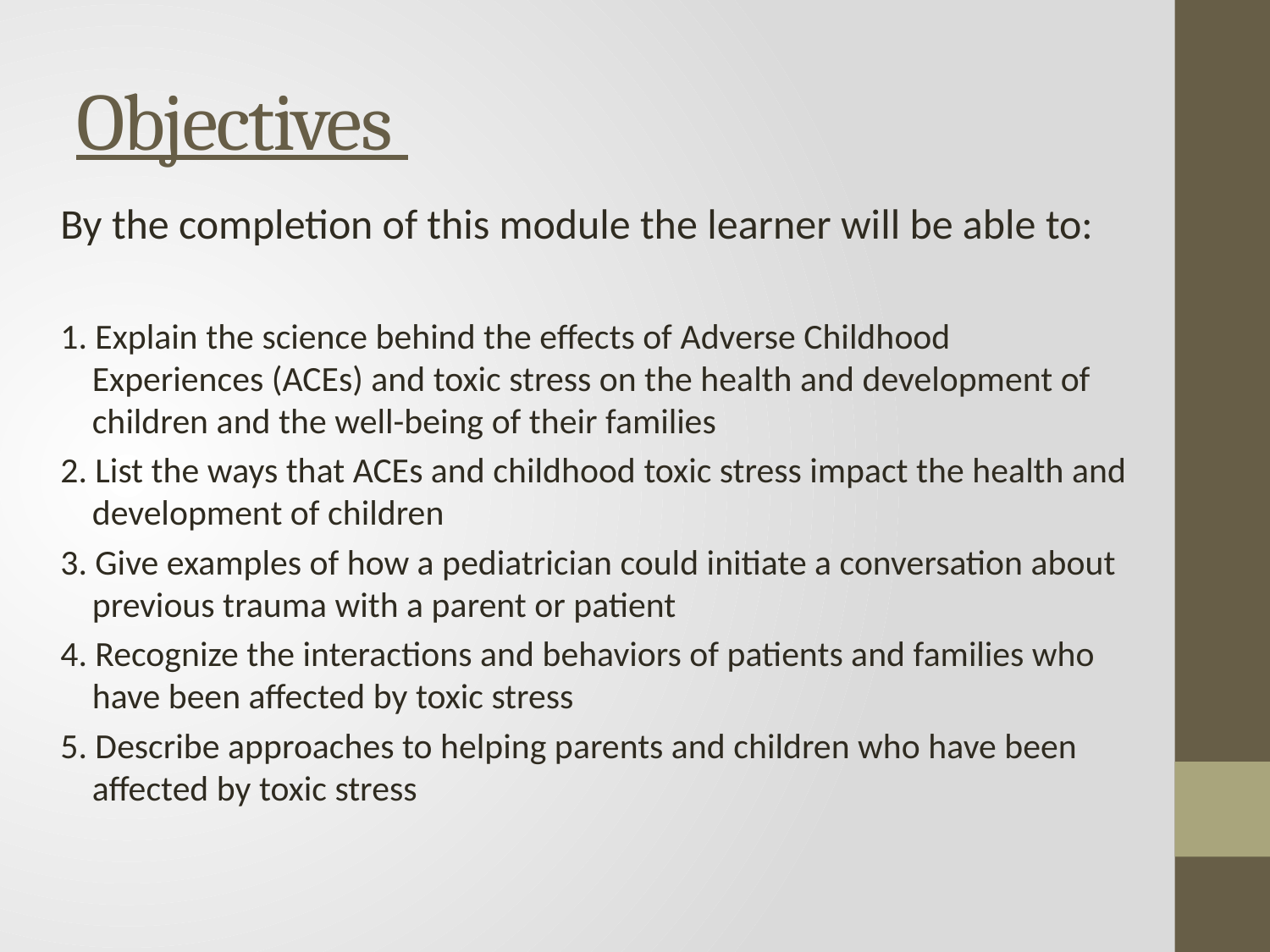

# Objectives
By the completion of this module the learner will be able to:
1. Explain the science behind the effects of Adverse Childhood Experiences (ACEs) and toxic stress on the health and development of children and the well-being of their families
2. List the ways that ACEs and childhood toxic stress impact the health and development of children
3. Give examples of how a pediatrician could initiate a conversation about previous trauma with a parent or patient
4. Recognize the interactions and behaviors of patients and families who have been affected by toxic stress
5. Describe approaches to helping parents and children who have been affected by toxic stress

## Slide 6
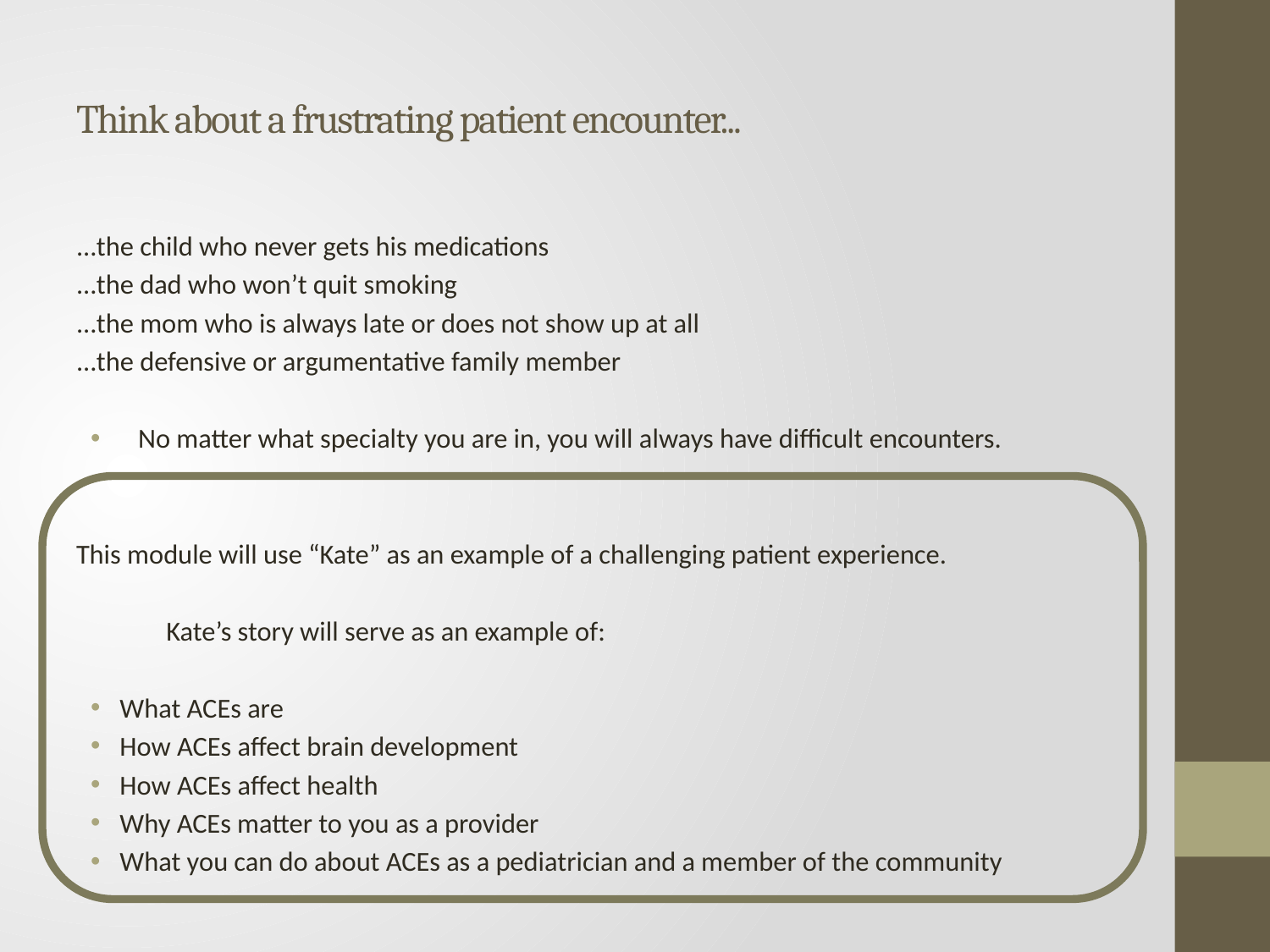

# Think about a frustrating patient encounter...
...the child who never gets his medications
...the dad who won’t quit smoking
...the mom who is always late or does not show up at all
...the defensive or argumentative family member
 No matter what specialty you are in, you will always have difficult encounters.
This module will use “Kate” as an example of a challenging patient experience.
	Kate’s story will serve as an example of:
What ACEs are
How ACEs affect brain development
How ACEs affect health
Why ACEs matter to you as a provider
What you can do about ACEs as a pediatrician and a member of the community

## Slide 7
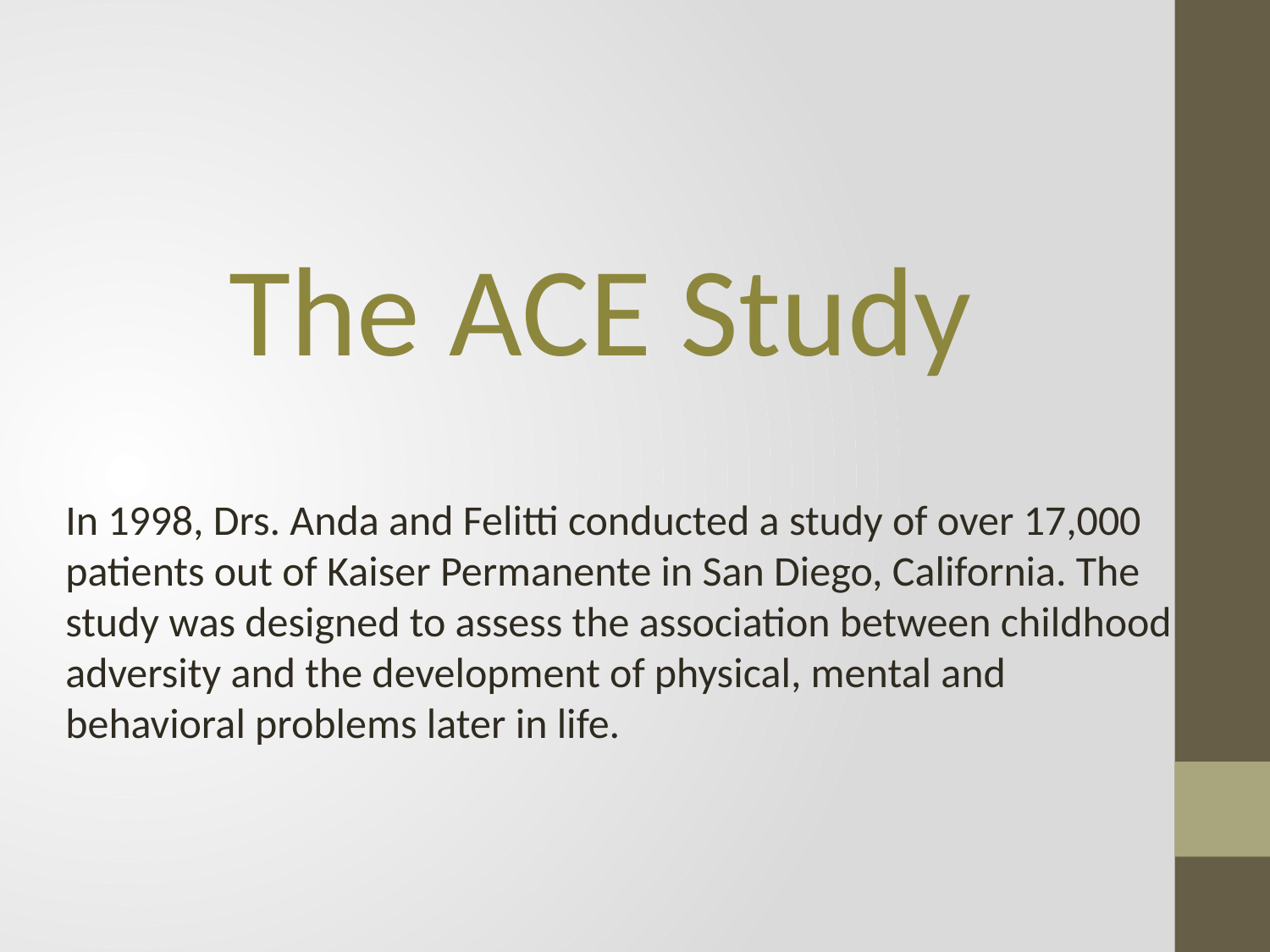

#
The ACE Study
In 1998, Drs. Anda and Felitti conducted a study of over 17,000 patients out of Kaiser Permanente in San Diego, California. The study was designed to assess the association between childhood adversity and the development of physical, mental and behavioral problems later in life.

## Slide 8
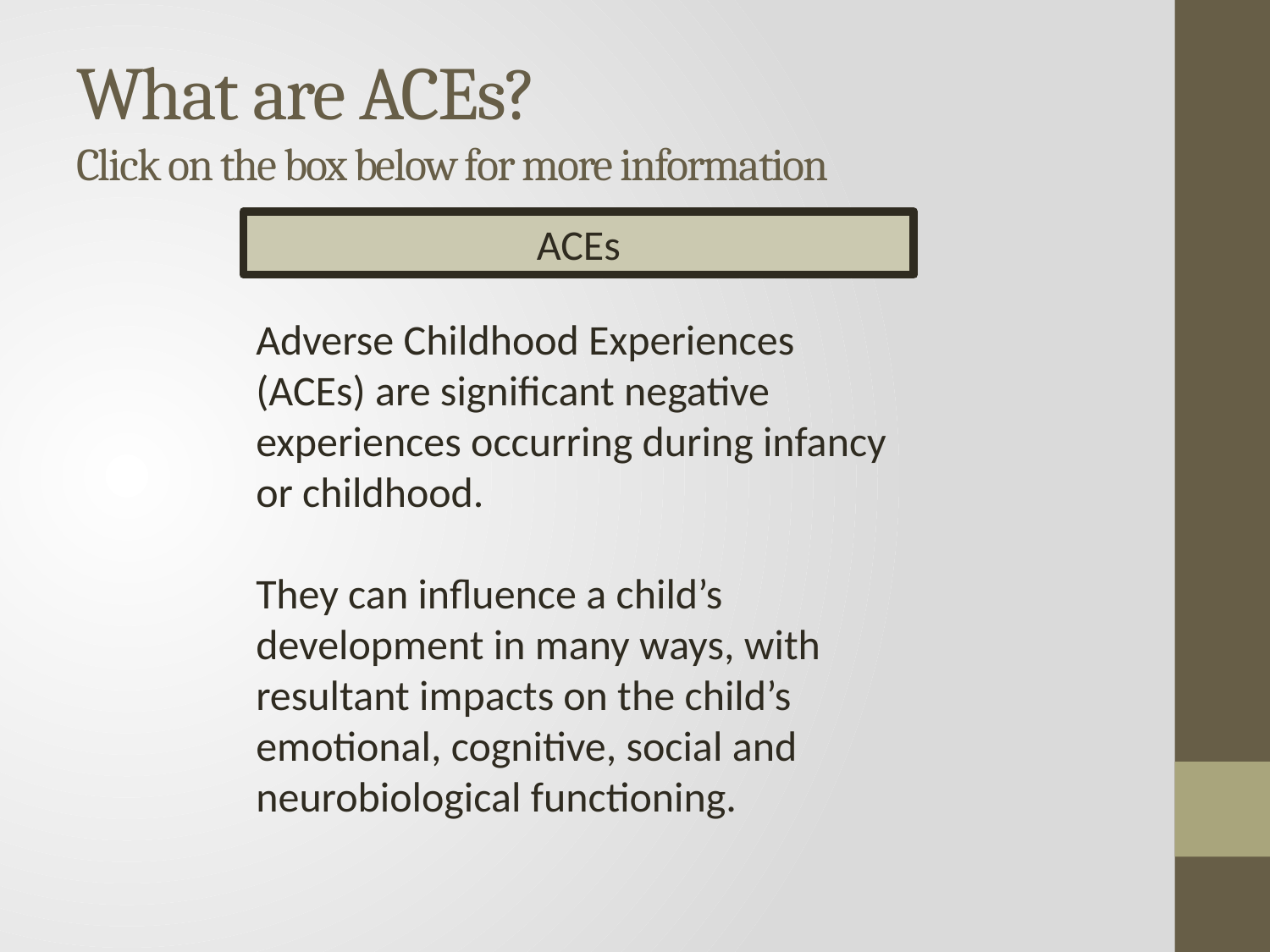

# What are ACEs? Click on the box below for more information
ACEs
Adverse Childhood Experiences (ACEs) are significant negative experiences occurring during infancy or childhood.
They can influence a child’s development in many ways, with resultant impacts on the child’s emotional, cognitive, social and neurobiological functioning.

## Slide 9
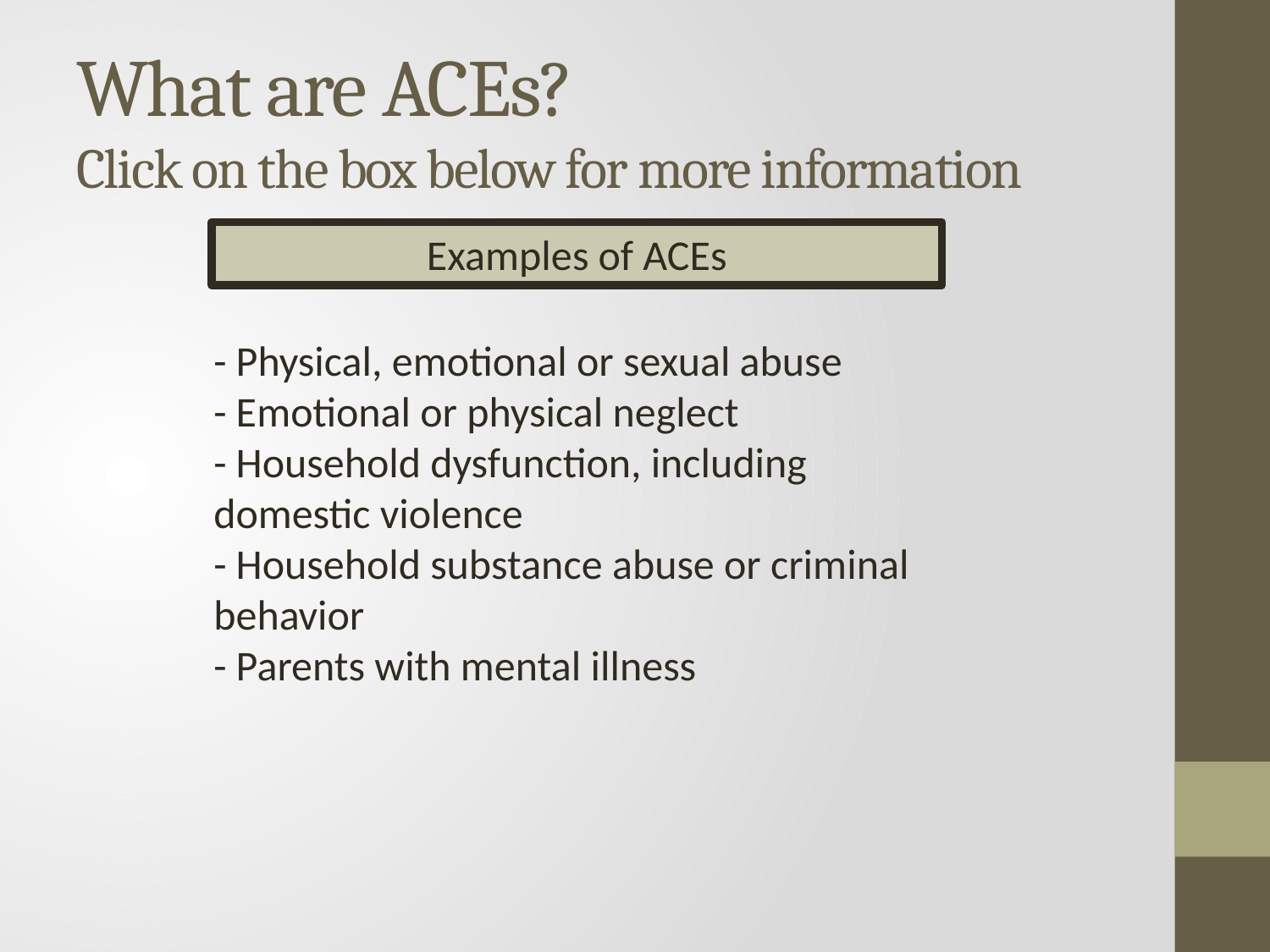

# What are ACEs? Click on the box below for more information
Examples of ACEs
- Physical, emotional or sexual abuse
- Emotional or physical neglect
- Household dysfunction, including domestic violence
- Household substance abuse or criminal behavior
- Parents with mental illness

## Slide 10
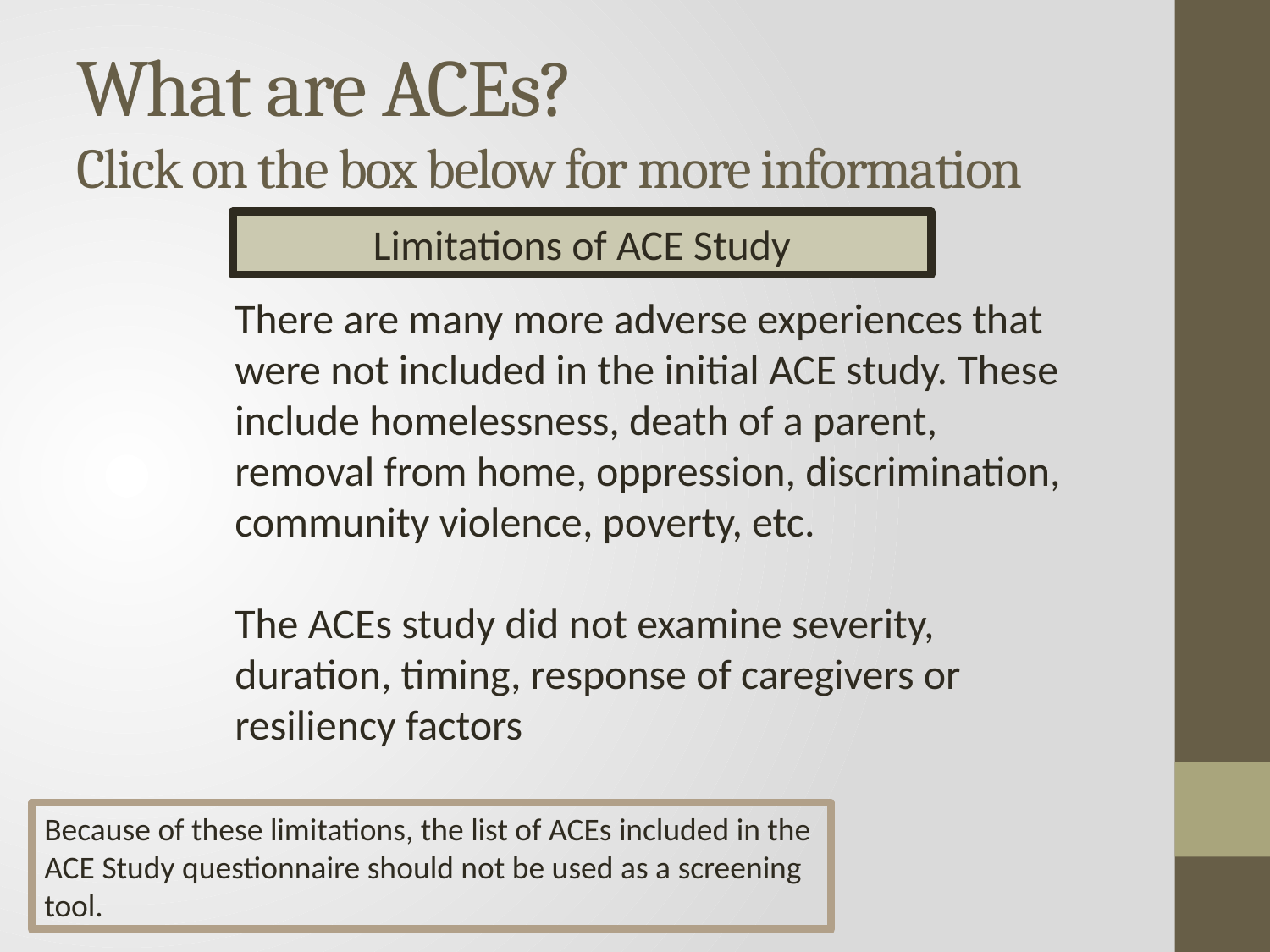

# What are ACEs? Click on the box below for more information
Limitations of ACE Study
There are many more adverse experiences that were not included in the initial ACE study. These include homelessness, death of a parent, removal from home, oppression, discrimination, community violence, poverty, etc.
The ACEs study did not examine severity, duration, timing, response of caregivers or resiliency factors
Because of these limitations, the list of ACEs included in the ACE Study questionnaire should not be used as a screening tool.

## Slide 11
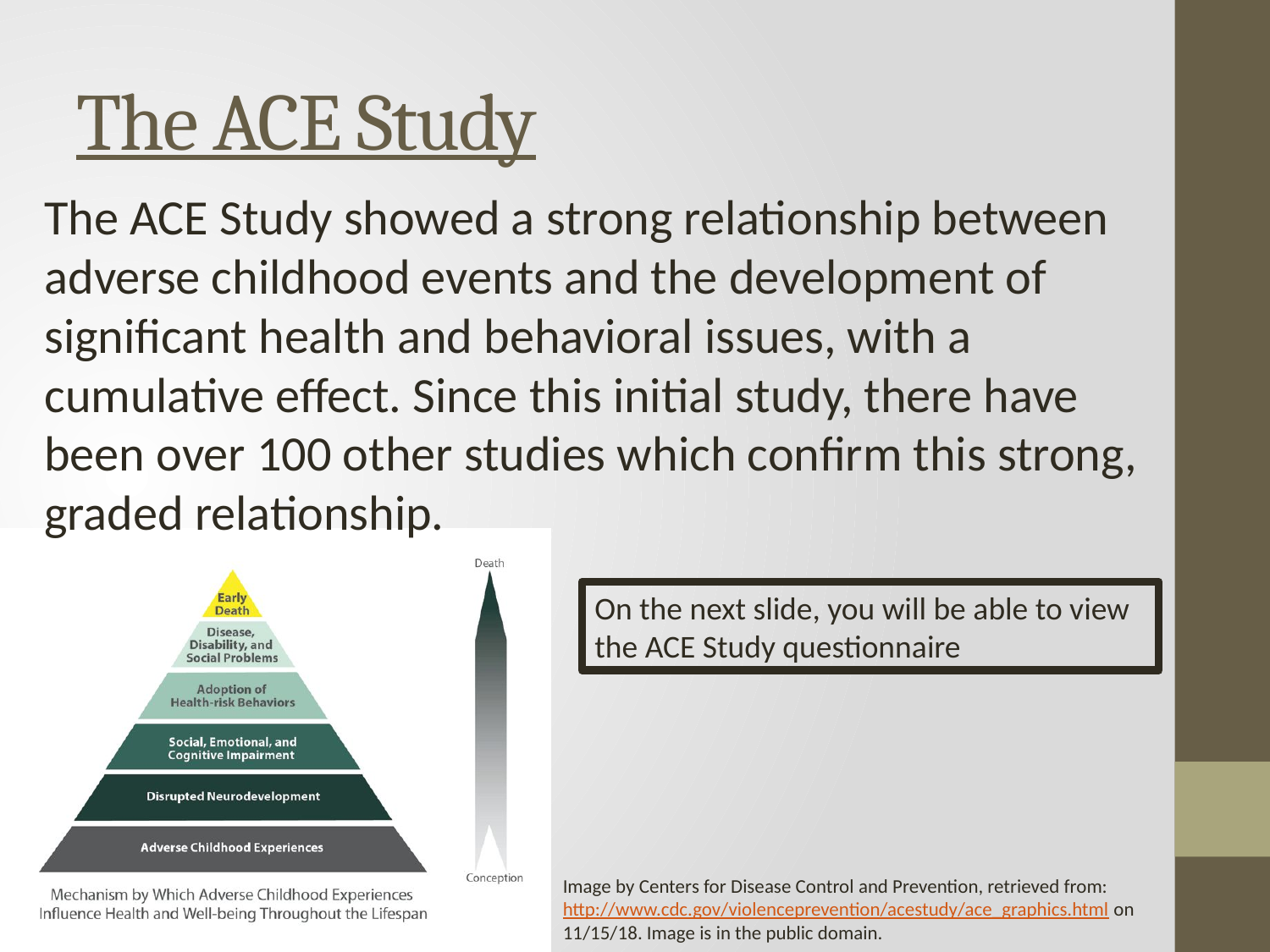

# The ACE Study
The ACE Study showed a strong relationship between adverse childhood events and the development of significant health and behavioral issues, with a cumulative effect. Since this initial study, there have been over 100 other studies which confirm this strong, graded relationship.
On the next slide, you will be able to view the ACE Study questionnaire
Image by Centers for Disease Control and Prevention, retrieved from: http://www.cdc.gov/violenceprevention/acestudy/ace_graphics.html on 11/15/18. Image is in the public domain.

## Slide 12
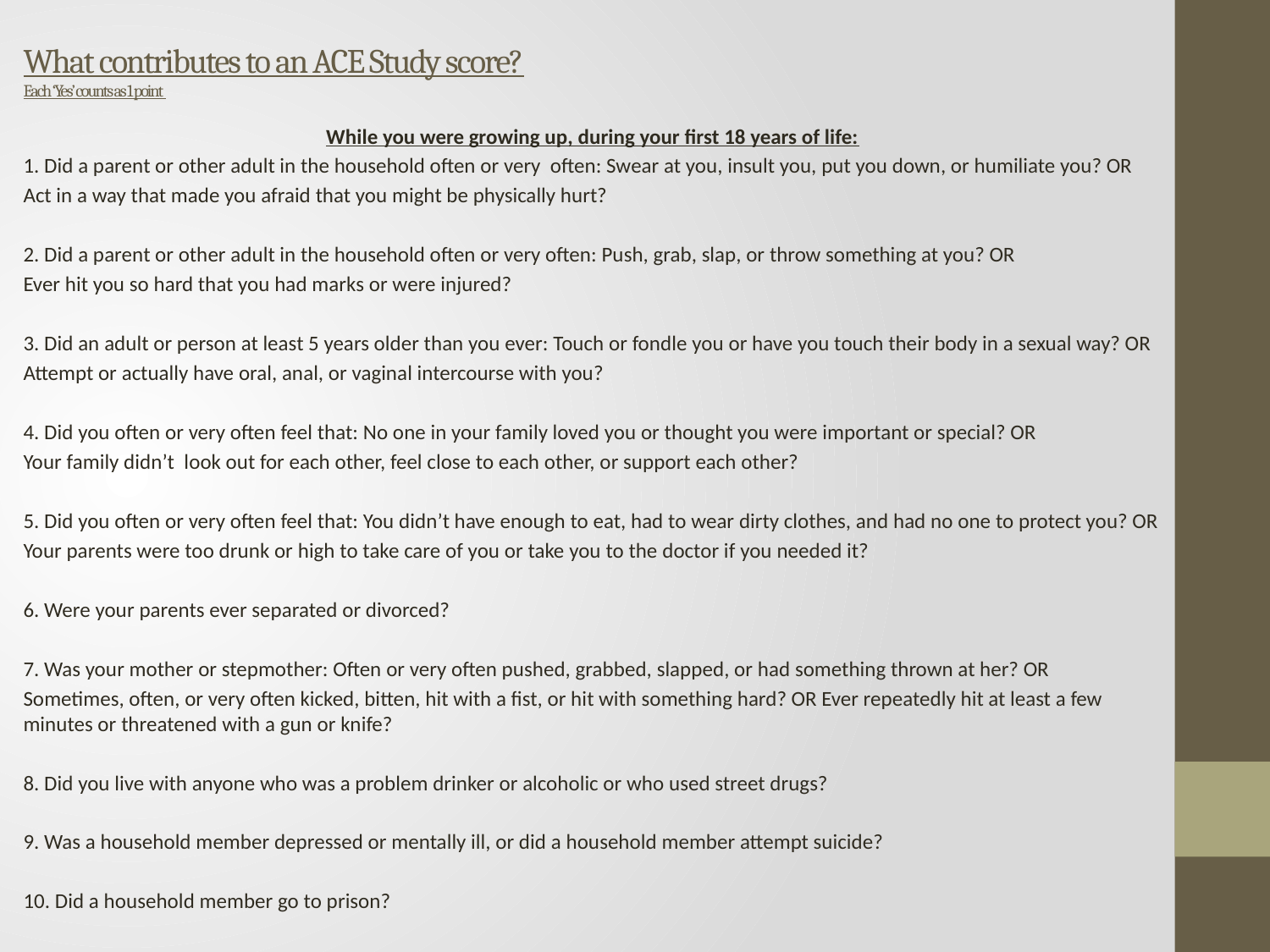

# What contributes to an ACE Study score? Each ‘Yes’ counts as 1 point
While you were growing up, during your first 18 years of life:
1. Did a parent or other adult in the household often or very often: Swear at you, insult you, put you down, or humiliate you? OR
Act in a way that made you afraid that you might be physically hurt?
2. Did a parent or other adult in the household often or very often: Push, grab, slap, or throw something at you? OR
Ever hit you so hard that you had marks or were injured?
3. Did an adult or person at least 5 years older than you ever: Touch or fondle you or have you touch their body in a sexual way? OR
Attempt or actually have oral, anal, or vaginal intercourse with you?
4. Did you often or very often feel that: No one in your family loved you or thought you were important or special? OR
Your family didn’t look out for each other, feel close to each other, or support each other?
5. Did you often or very often feel that: You didn’t have enough to eat, had to wear dirty clothes, and had no one to protect you? OR
Your parents were too drunk or high to take care of you or take you to the doctor if you needed it?
6. Were your parents ever separated or divorced?
7. Was your mother or stepmother: Often or very often pushed, grabbed, slapped, or had something thrown at her? OR
Sometimes, often, or very often kicked, bitten, hit with a fist, or hit with something hard? OR Ever repeatedly hit at least a few minutes or threatened with a gun or knife?
8. Did you live with anyone who was a problem drinker or alcoholic or who used street drugs?
9. Was a household member depressed or mentally ill, or did a household member attempt suicide?
10. Did a household member go to prison?

## Slide 13
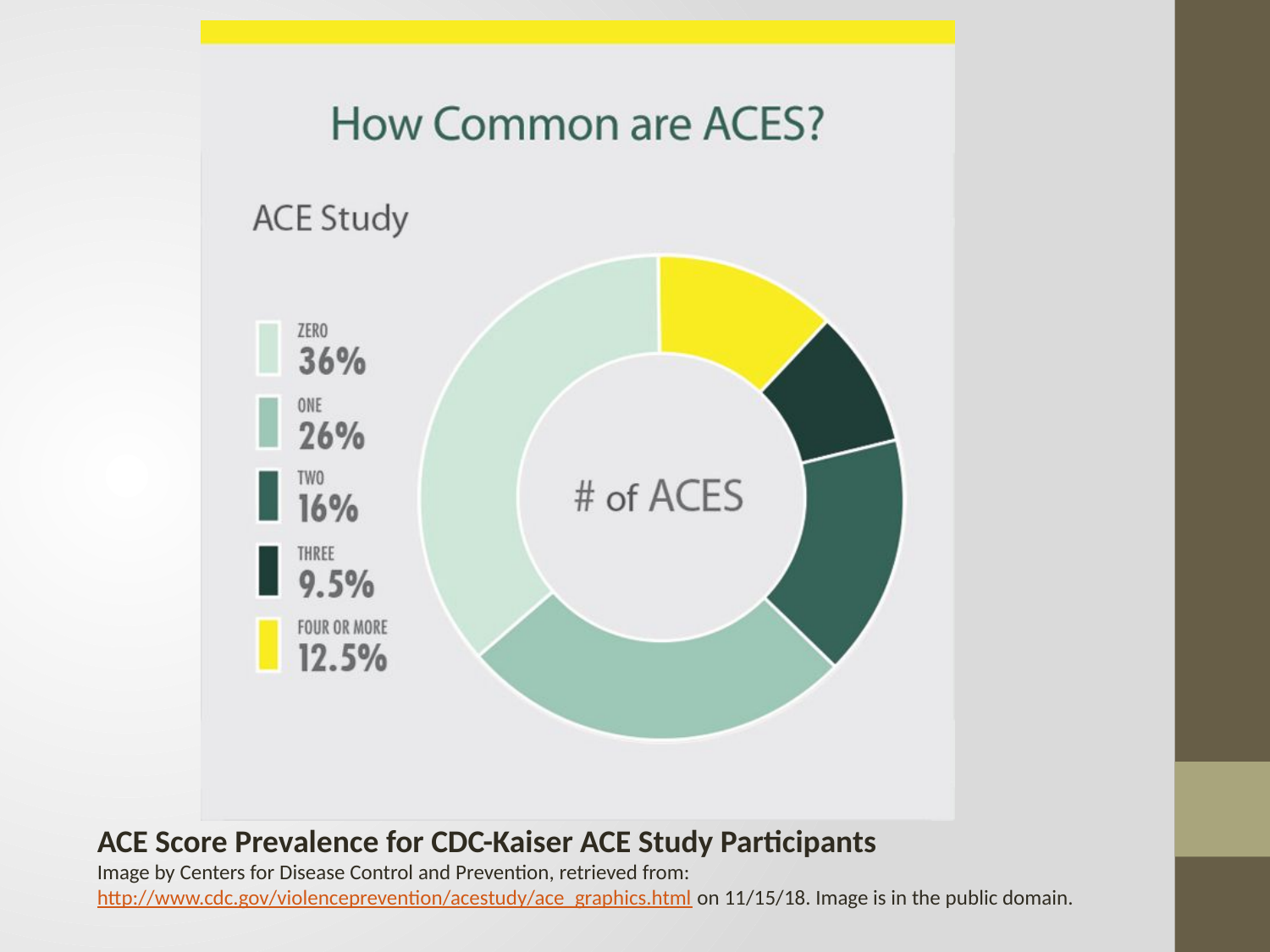

#
ACE Score Prevalence for CDC-Kaiser ACE Study Participants
Image by Centers for Disease Control and Prevention, retrieved from: http://www.cdc.gov/violenceprevention/acestudy/ace_graphics.html on 11/15/18. Image is in the public domain.

## Slide 14
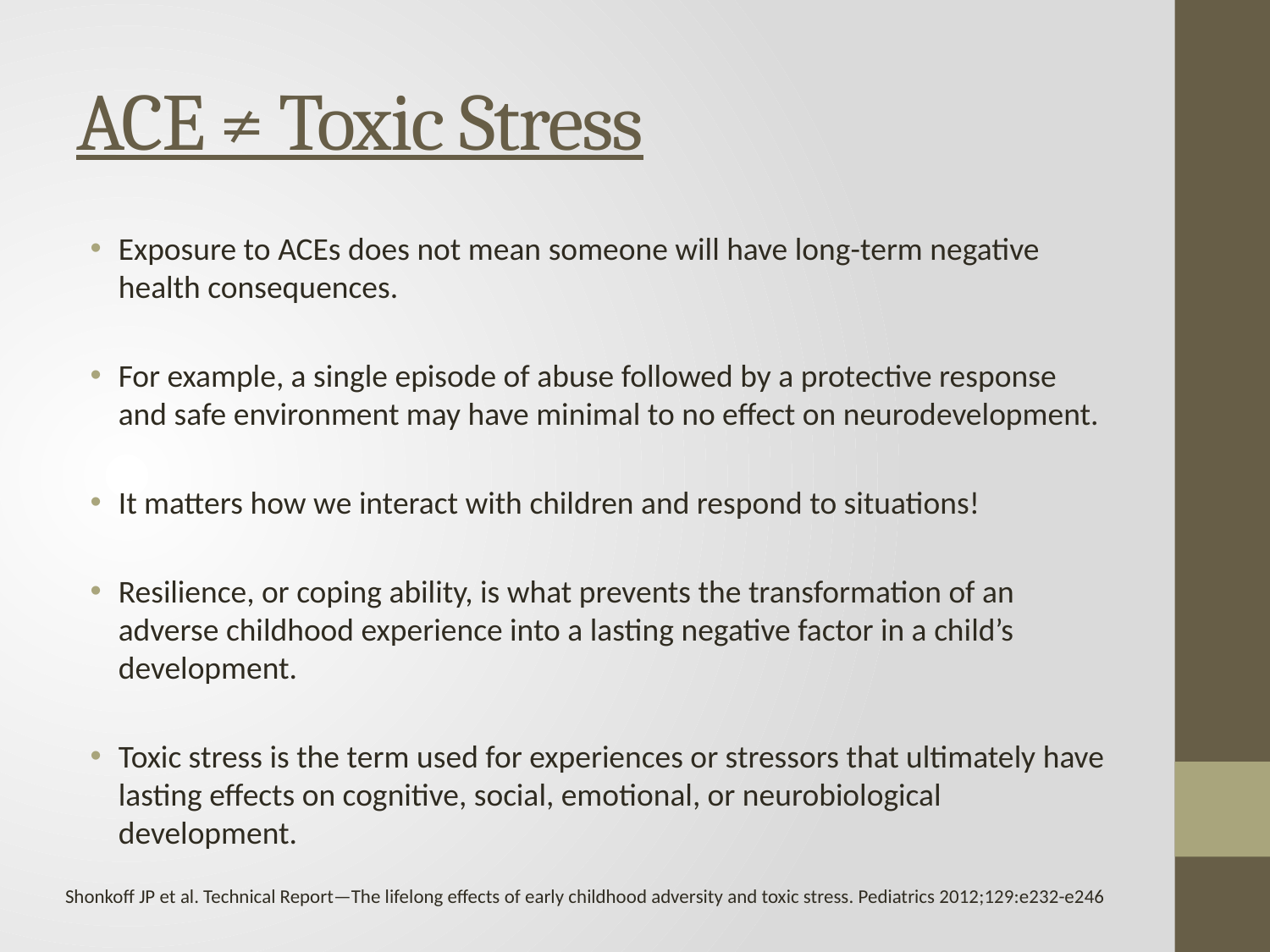

# ACE ≠ Toxic Stress
Exposure to ACEs does not mean someone will have long-term negative health consequences.
For example, a single episode of abuse followed by a protective response and safe environment may have minimal to no effect on neurodevelopment.
It matters how we interact with children and respond to situations!
Resilience, or coping ability, is what prevents the transformation of an adverse childhood experience into a lasting negative factor in a child’s development.
Toxic stress is the term used for experiences or stressors that ultimately have lasting effects on cognitive, social, emotional, or neurobiological development.
Shonkoff JP et al. Technical Report—The lifelong effects of early childhood adversity and toxic stress. Pediatrics 2012;129:e232-e246

## Slide 15
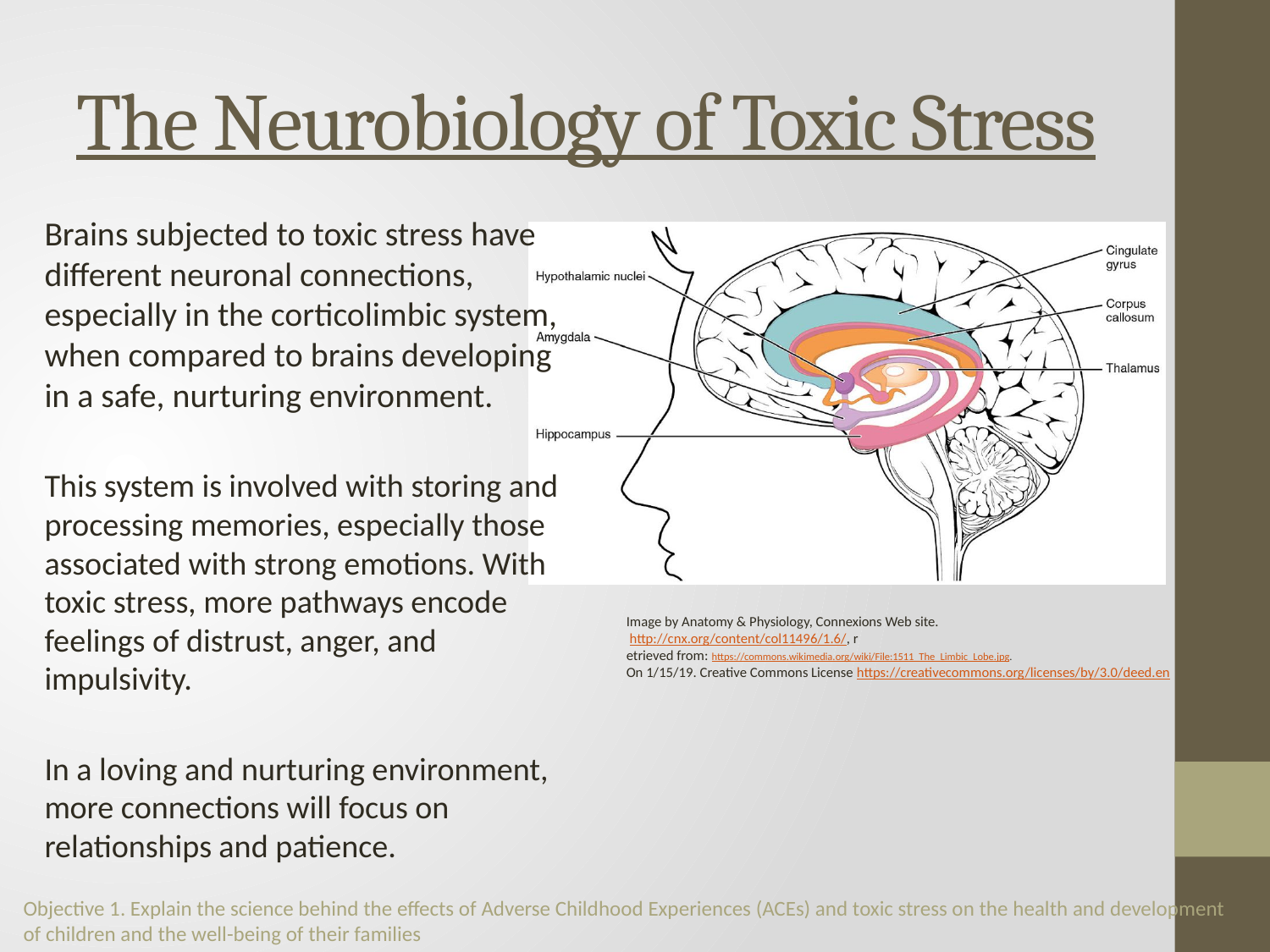

# The Neurobiology of Toxic Stress
Brains subjected to toxic stress have different neuronal connections, especially in the corticolimbic system, when compared to brains developing in a safe, nurturing environment.
This system is involved with storing and processing memories, especially those associated with strong emotions. With toxic stress, more pathways encode feelings of distrust, anger, and impulsivity.
In a loving and nurturing environment, more connections will focus on relationships and patience.
Image by Anatomy & Physiology, Connexions Web site.
 http://cnx.org/content/col11496/1.6/, r
etrieved from: https://commons.wikimedia.org/wiki/File:1511_The_Limbic_Lobe.jpg.
On 1/15/19. Creative Commons License https://creativecommons.org/licenses/by/3.0/deed.en
Objective 1. Explain the science behind the effects of Adverse Childhood Experiences (ACEs) and toxic stress on the health and development of children and the well-being of their families

## Slide 16
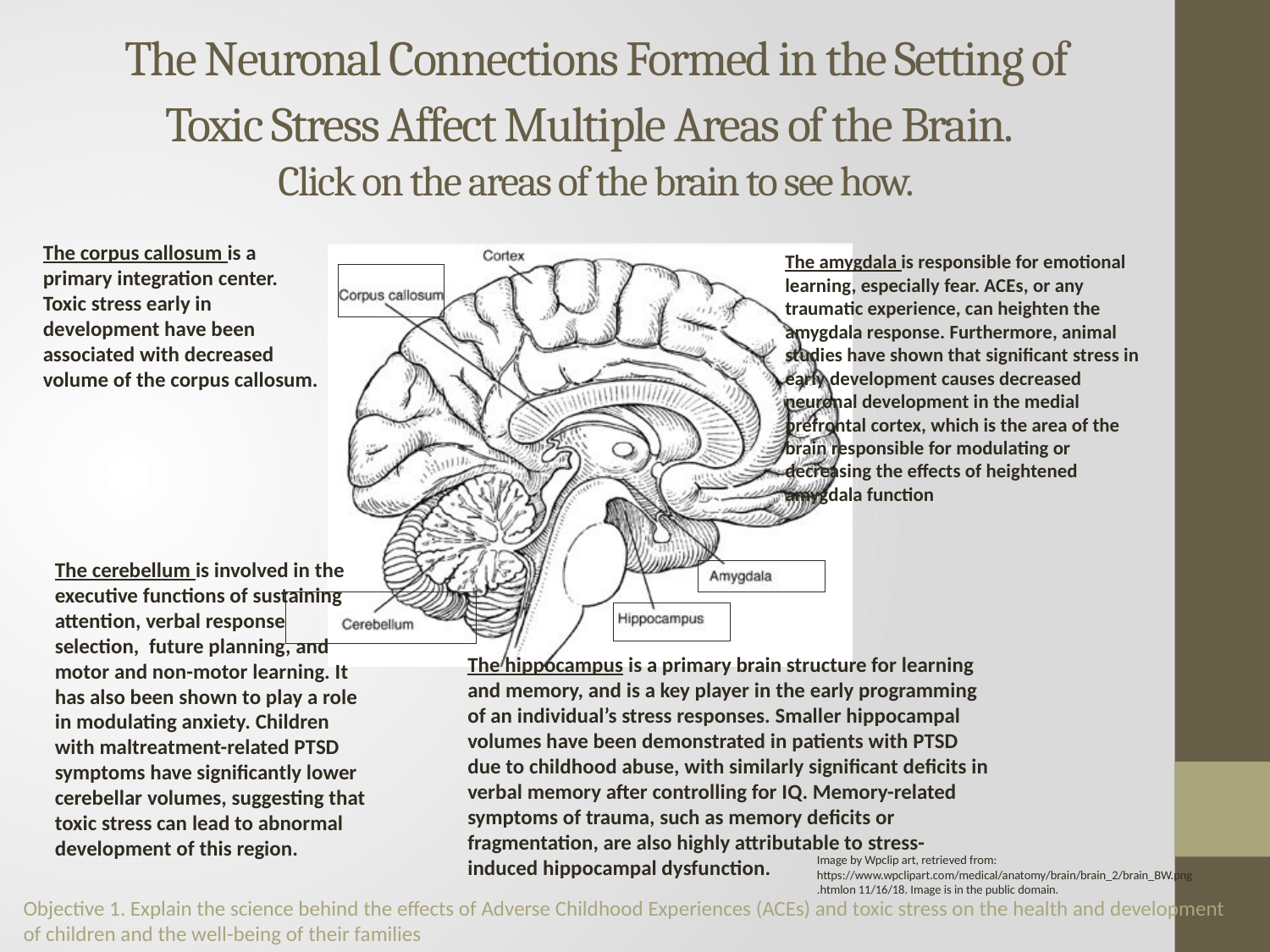

# The Neuronal Connections Formed in the Setting of Toxic Stress Affect Multiple Areas of the Brain. Click on the areas of the brain to see how.
The corpus callosum is a primary integration center. Toxic stress early in development have been associated with decreased volume of the corpus callosum.
The amygdala is responsible for emotional learning, especially fear. ACEs, or any traumatic experience, can heighten the amygdala response. Furthermore, animal studies have shown that significant stress in early development causes decreased neuronal development in the medial prefrontal cortex, which is the area of the brain responsible for modulating or decreasing the effects of heightened amygdala function
The cerebellum is involved in the executive functions of sustaining attention, verbal response selection, future planning, and motor and non-motor learning. It has also been shown to play a role in modulating anxiety. Children with maltreatment-related PTSD symptoms have significantly lower cerebellar volumes, suggesting that toxic stress can lead to abnormal development of this region.
The hippocampus is a primary brain structure for learning and memory, and is a key player in the early programming of an individual’s stress responses. Smaller hippocampal volumes have been demonstrated in patients with PTSD due to childhood abuse, with similarly significant deficits in verbal memory after controlling for IQ. Memory-related symptoms of trauma, such as memory deficits or fragmentation, are also highly attributable to stress-induced hippocampal dysfunction.
Image by Wpclip art, retrieved from: https://www.wpclipart.com/medical/anatomy/brain/brain_2/brain_BW.png.htmlon 11/16/18. Image is in the public domain.
Objective 1. Explain the science behind the effects of Adverse Childhood Experiences (ACEs) and toxic stress on the health and development of children and the well-being of their families

## Slide 17
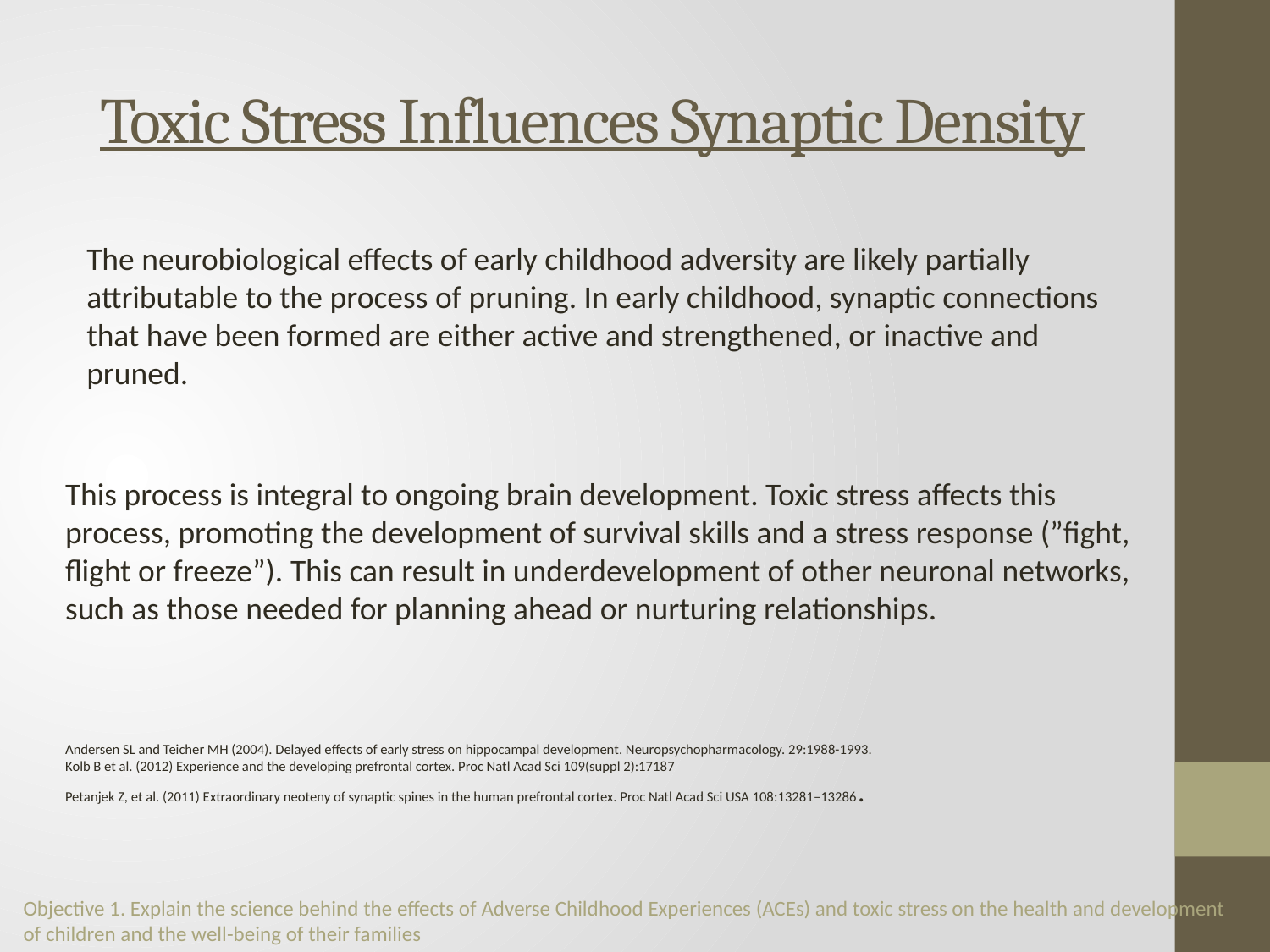

# Toxic Stress Influences Synaptic Density
The neurobiological effects of early childhood adversity are likely partially attributable to the process of pruning. In early childhood, synaptic connections that have been formed are either active and strengthened, or inactive and pruned.
This process is integral to ongoing brain development. Toxic stress affects this process, promoting the development of survival skills and a stress response (”fight, flight or freeze”). This can result in underdevelopment of other neuronal networks, such as those needed for planning ahead or nurturing relationships.
Andersen SL and Teicher MH (2004). Delayed effects of early stress on hippocampal development. Neuropsychopharmacology. 29:1988-1993.
Kolb B et al. (2012) Experience and the developing prefrontal cortex. Proc Natl Acad Sci 109(suppl 2):17187
Petanjek Z, et al. (2011) Extraordinary neoteny of synaptic spines in the human prefrontal cortex. Proc Natl Acad Sci USA 108:13281–13286.
Objective 1. Explain the science behind the effects of Adverse Childhood Experiences (ACEs) and toxic stress on the health and development of children and the well-being of their families

## Slide 18
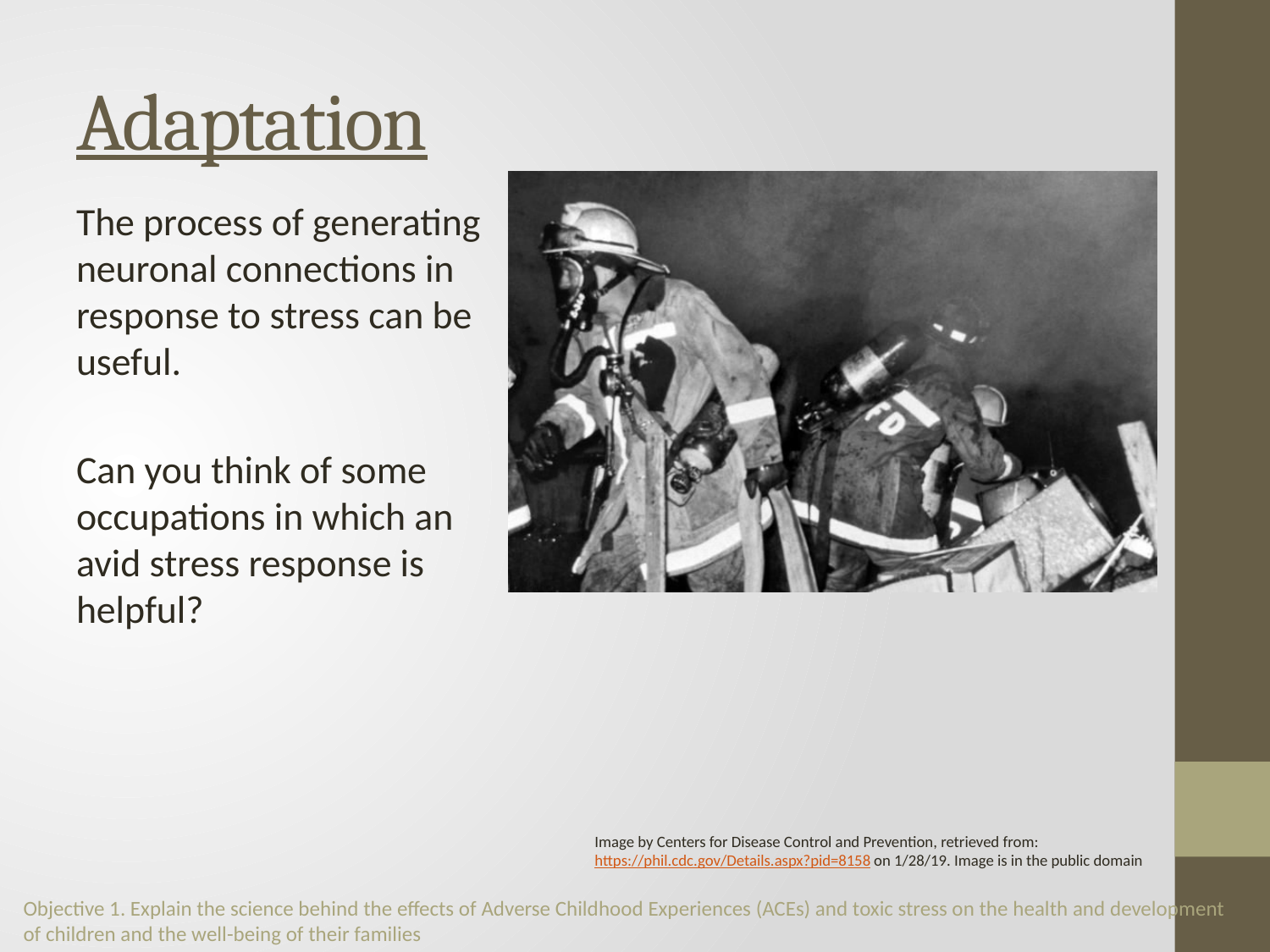

# Adaptation
The process of generating neuronal connections in response to stress can be useful.
Can you think of some occupations in which an avid stress response is helpful?
Image by Centers for Disease Control and Prevention, retrieved from: https://phil.cdc.gov/Details.aspx?pid=8158 on 1/28/19. Image is in the public domain
Objective 1. Explain the science behind the effects of Adverse Childhood Experiences (ACEs) and toxic stress on the health and development of children and the well-being of their families

## Slide 19
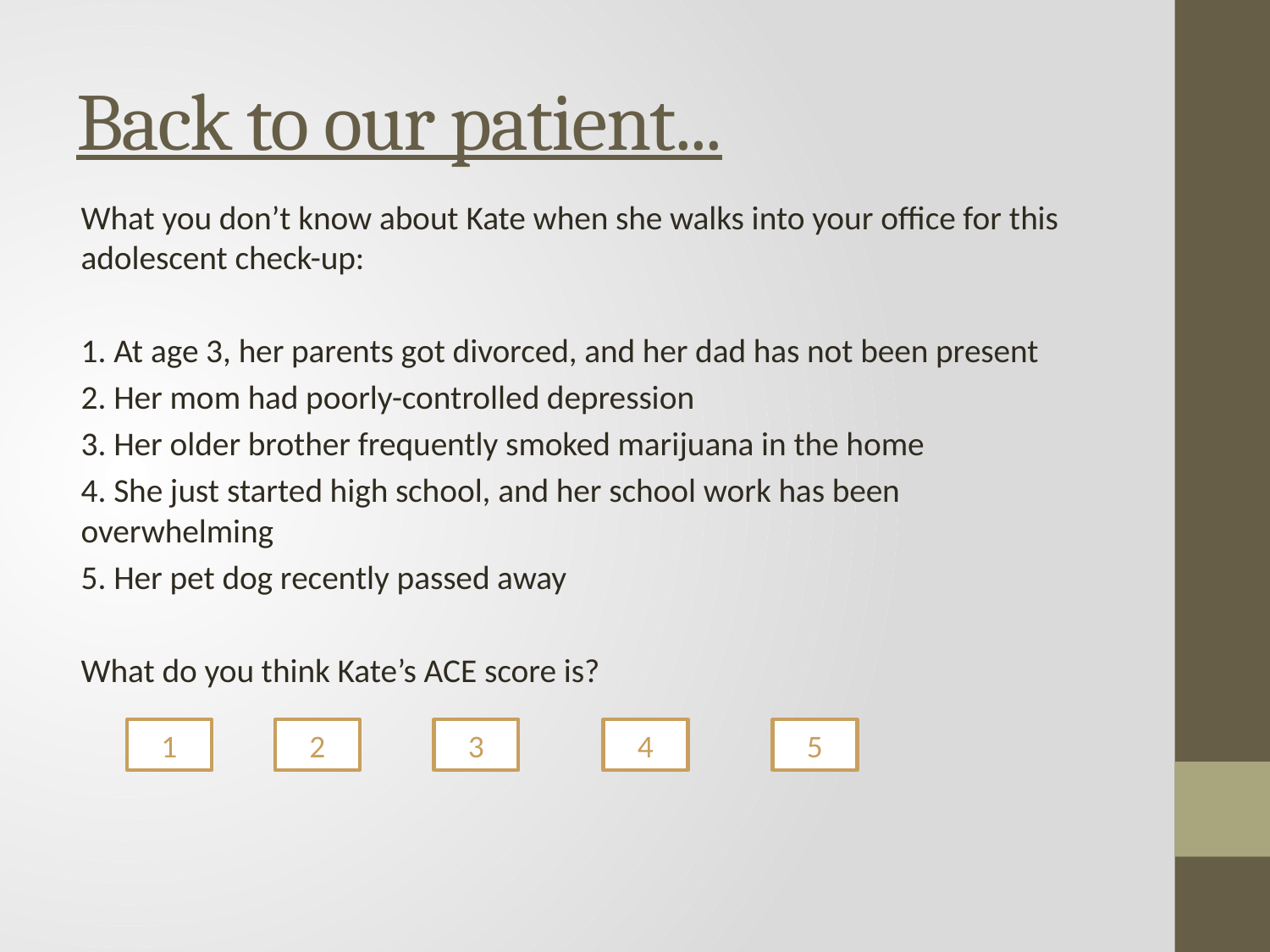

# Back to our patient...
What you don’t know about Kate when she walks into your office for this adolescent check-up:
1. At age 3, her parents got divorced, and her dad has not been present
2. Her mom had poorly-controlled depression
3. Her older brother frequently smoked marijuana in the home
4. She just started high school, and her school work has been overwhelming
5. Her pet dog recently passed away
What do you think Kate’s ACE score is?
1
2
3
4
5

## Slide 20
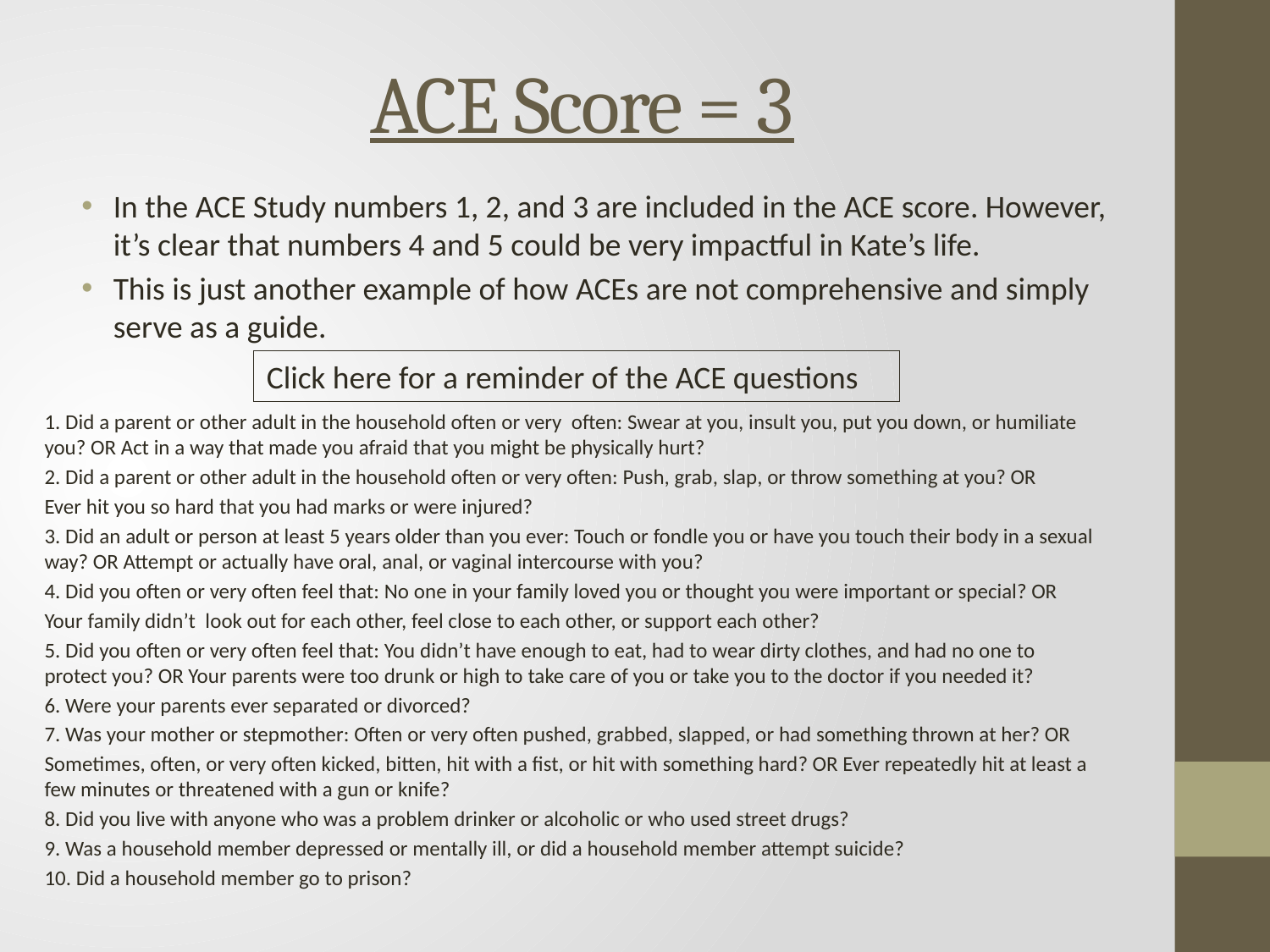

# ACE Score = 3
In the ACE Study numbers 1, 2, and 3 are included in the ACE score. However, it’s clear that numbers 4 and 5 could be very impactful in Kate’s life.
This is just another example of how ACEs are not comprehensive and simply serve as a guide.
Click here for a reminder of the ACE questions
1. Did a parent or other adult in the household often or very often: Swear at you, insult you, put you down, or humiliate you? OR Act in a way that made you afraid that you might be physically hurt?
2. Did a parent or other adult in the household often or very often: Push, grab, slap, or throw something at you? OR
Ever hit you so hard that you had marks or were injured?
3. Did an adult or person at least 5 years older than you ever: Touch or fondle you or have you touch their body in a sexual way? OR Attempt or actually have oral, anal, or vaginal intercourse with you?
4. Did you often or very often feel that: No one in your family loved you or thought you were important or special? OR
Your family didn’t look out for each other, feel close to each other, or support each other?
5. Did you often or very often feel that: You didn’t have enough to eat, had to wear dirty clothes, and had no one to protect you? OR Your parents were too drunk or high to take care of you or take you to the doctor if you needed it?
6. Were your parents ever separated or divorced?
7. Was your mother or stepmother: Often or very often pushed, grabbed, slapped, or had something thrown at her? OR
Sometimes, often, or very often kicked, bitten, hit with a fist, or hit with something hard? OR Ever repeatedly hit at least a few minutes or threatened with a gun or knife?
8. Did you live with anyone who was a problem drinker or alcoholic or who used street drugs?
9. Was a household member depressed or mentally ill, or did a household member attempt suicide?
10. Did a household member go to prison?

## Slide 21
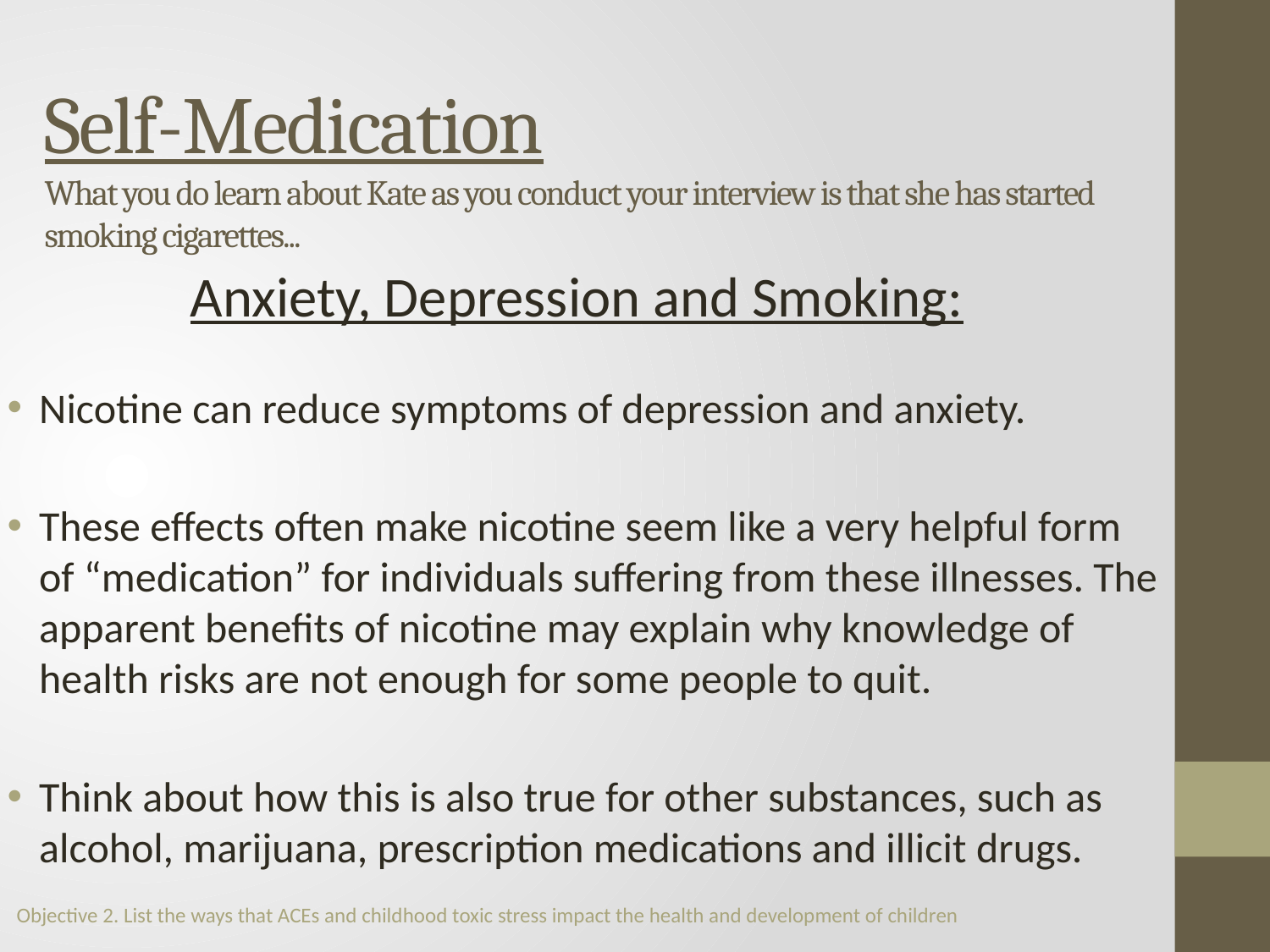

# Self-Medication What you do learn about Kate as you conduct your interview is that she has started smoking cigarettes...
Anxiety, Depression and Smoking:
Nicotine can reduce symptoms of depression and anxiety.
These effects often make nicotine seem like a very helpful form of “medication” for individuals suffering from these illnesses. The apparent benefits of nicotine may explain why knowledge of health risks are not enough for some people to quit.
Think about how this is also true for other substances, such as alcohol, marijuana, prescription medications and illicit drugs.
Objective 2. List the ways that ACEs and childhood toxic stress impact the health and development of children

## Slide 22
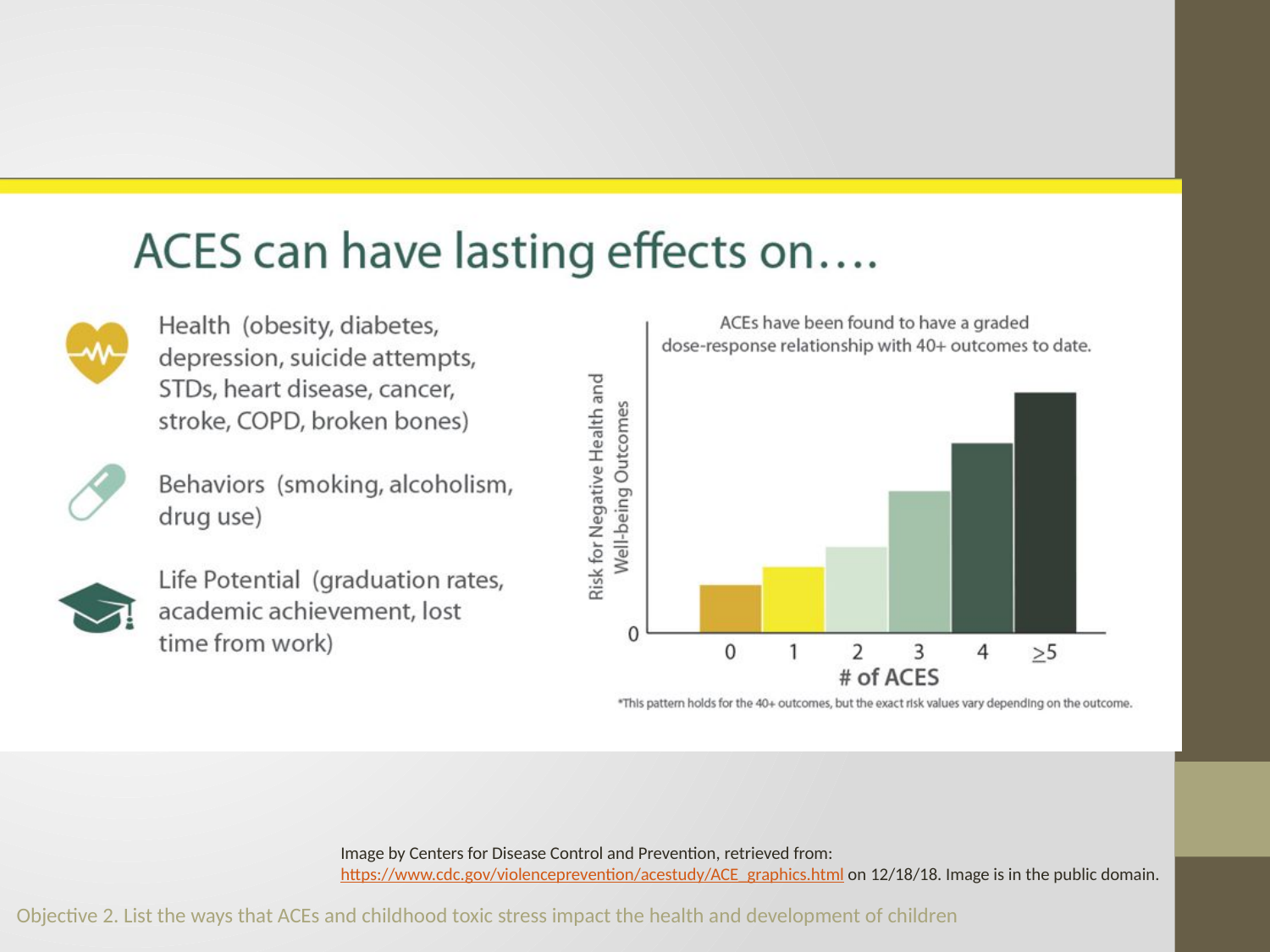

#
Image by Centers for Disease Control and Prevention, retrieved from: https://www.cdc.gov/violenceprevention/acestudy/ACE_graphics.html on 12/18/18. Image is in the public domain.
Objective 2. List the ways that ACEs and childhood toxic stress impact the health and development of children

## Slide 23
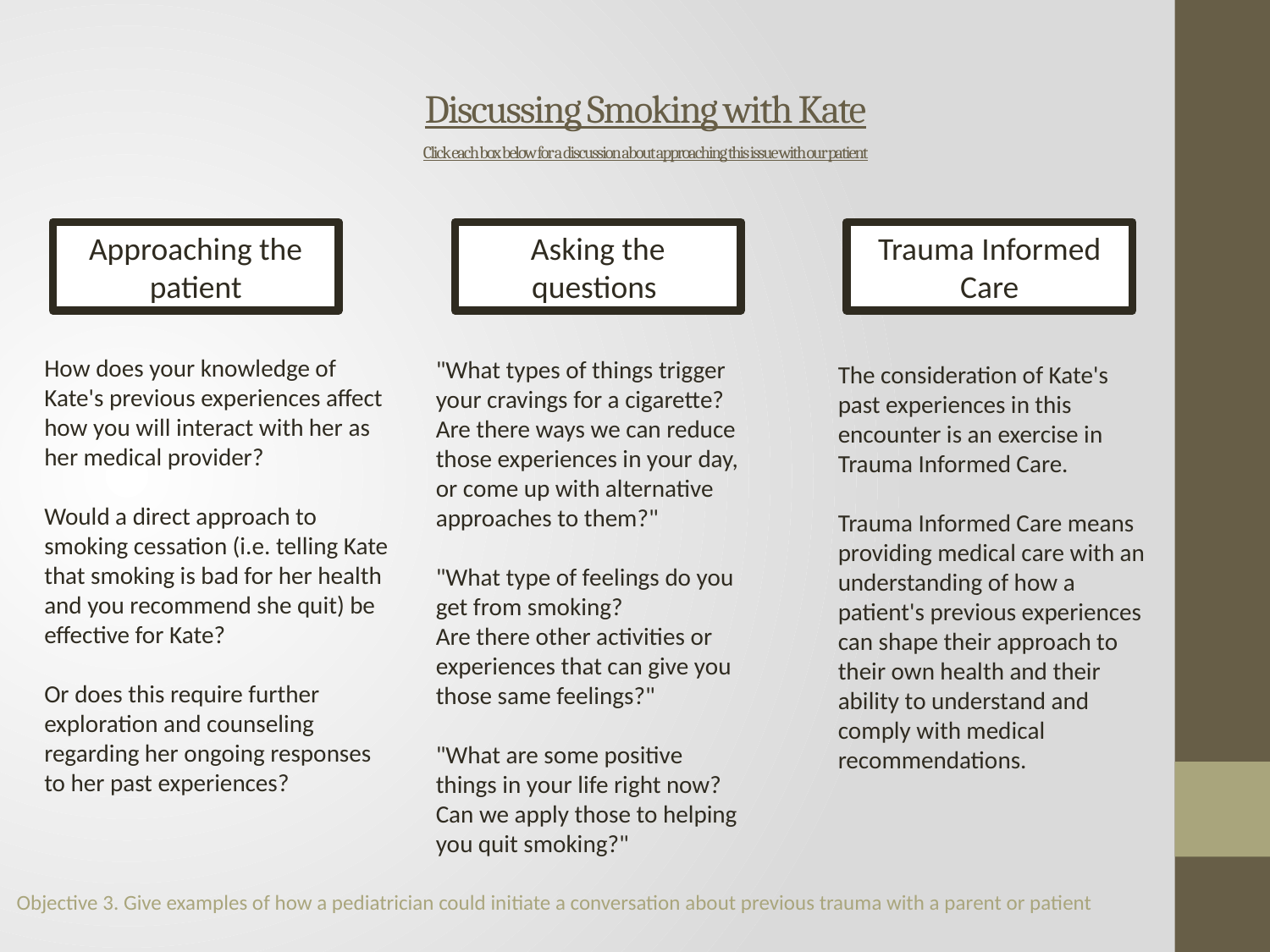

# Discussing Smoking with Kate Click each box below for a discussion about approaching this issue with our patient
Approaching the patient
Asking the questions
Trauma Informed Care
How does your knowledge of Kate's previous experiences affect how you will interact with her as her medical provider?
Would a direct approach to smoking cessation (i.e. telling Kate that smoking is bad for her health and you recommend she quit) be effective for Kate?
Or does this require further exploration and counseling regarding her ongoing responses to her past experiences?
"What types of things trigger your cravings for a cigarette? Are there ways we can reduce those experiences in your day, or come up with alternative approaches to them?"
"What type of feelings do you get from smoking?
Are there other activities or experiences that can give you those same feelings?"
"What are some positive things in your life right now? Can we apply those to helping you quit smoking?"
The consideration of Kate's past experiences in this encounter is an exercise in Trauma Informed Care.
Trauma Informed Care means providing medical care with an understanding of how a patient's previous experiences can shape their approach to their own health and their ability to understand and comply with medical recommendations.
Objective 3. Give examples of how a pediatrician could initiate a conversation about previous trauma with a parent or patient

## Slide 24
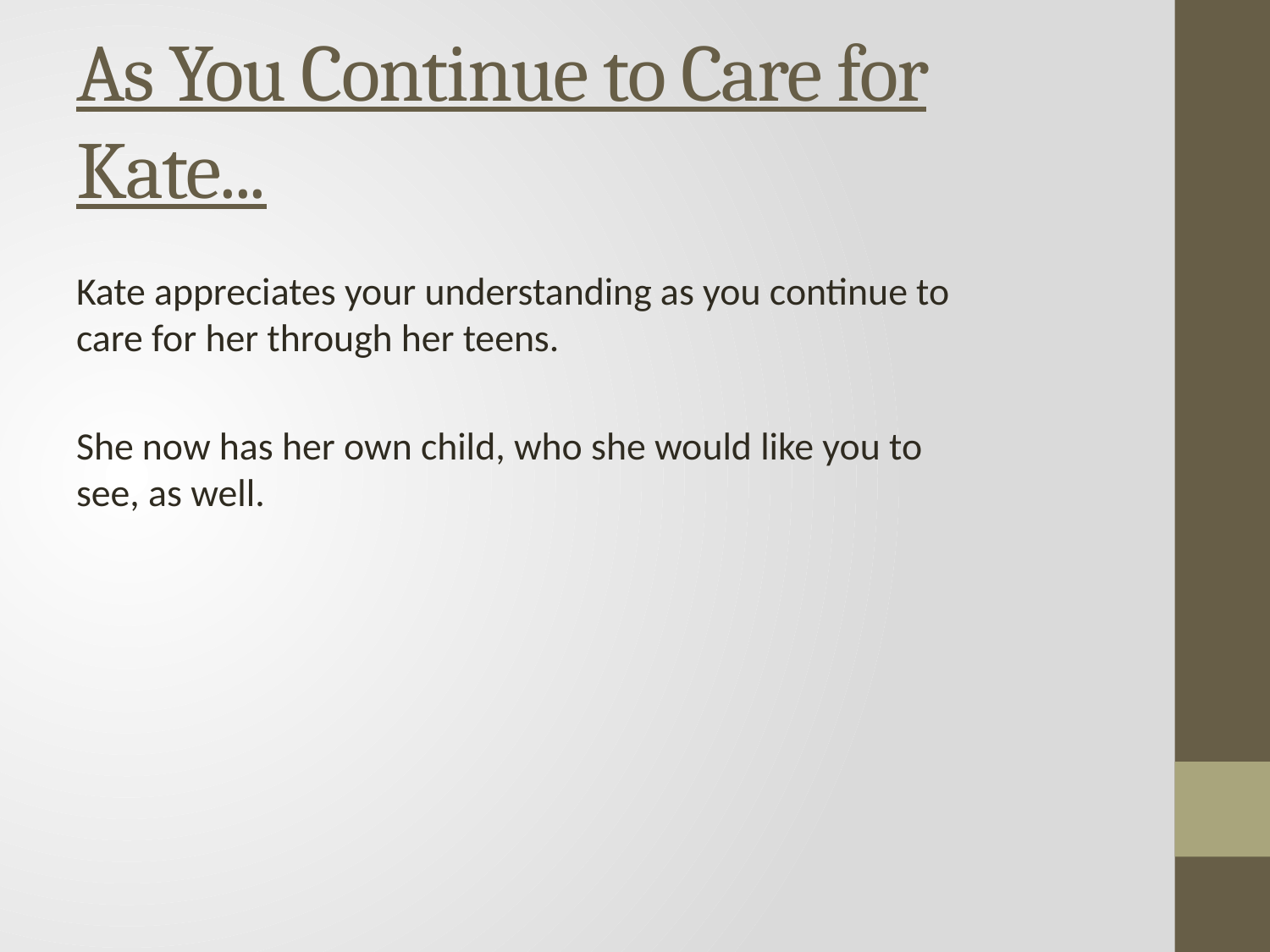

# As You Continue to Care for Kate...
Kate appreciates your understanding as you continue to care for her through her teens.
She now has her own child, who she would like you to see, as well.

## Slide 25
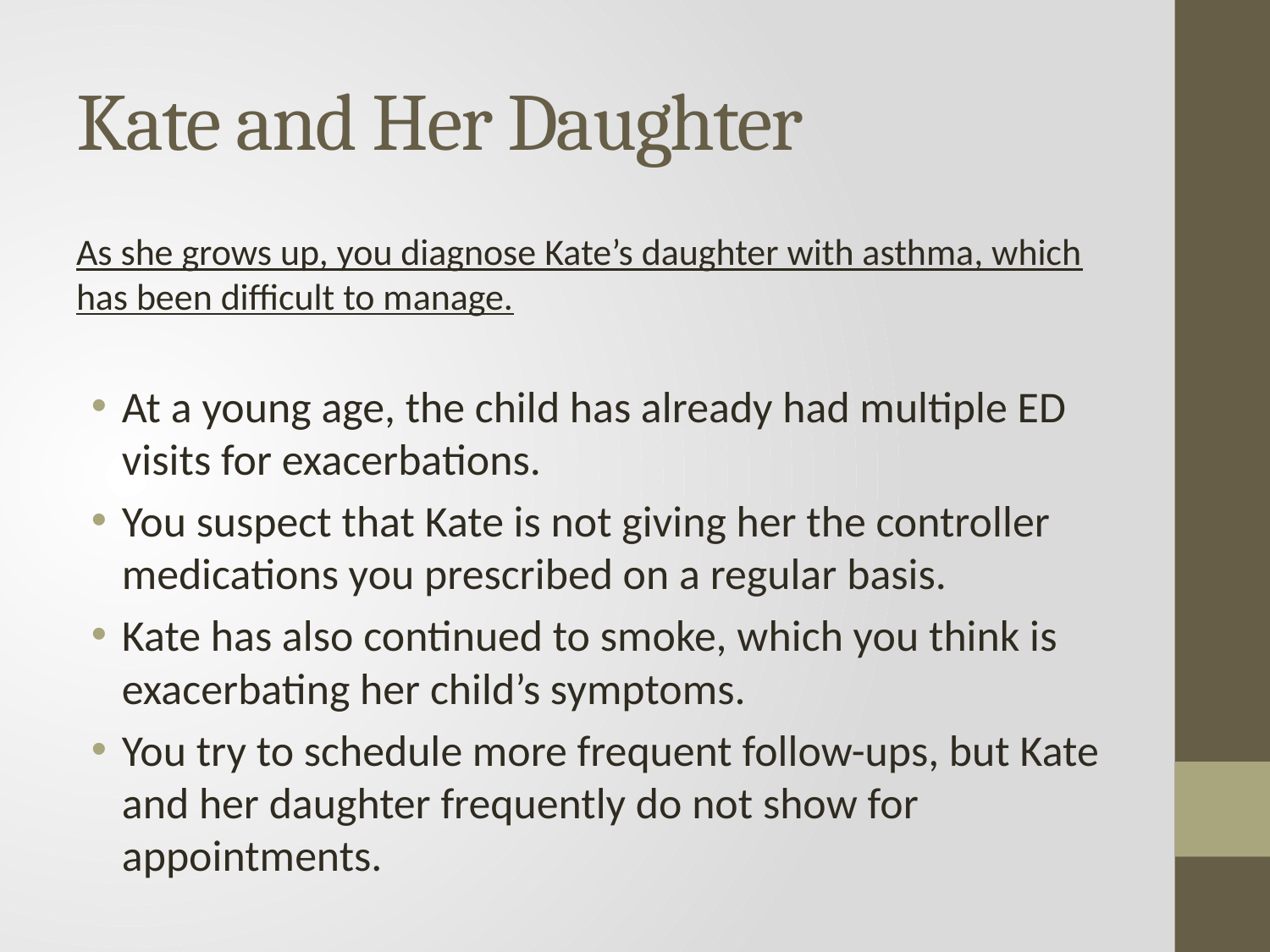

# Kate and Her Daughter
As she grows up, you diagnose Kate’s daughter with asthma, which has been difficult to manage.
At a young age, the child has already had multiple ED visits for exacerbations.
You suspect that Kate is not giving her the controller medications you prescribed on a regular basis.
Kate has also continued to smoke, which you think is exacerbating her child’s symptoms.
You try to schedule more frequent follow-ups, but Kate and her daughter frequently do not show for appointments.

## Slide 26
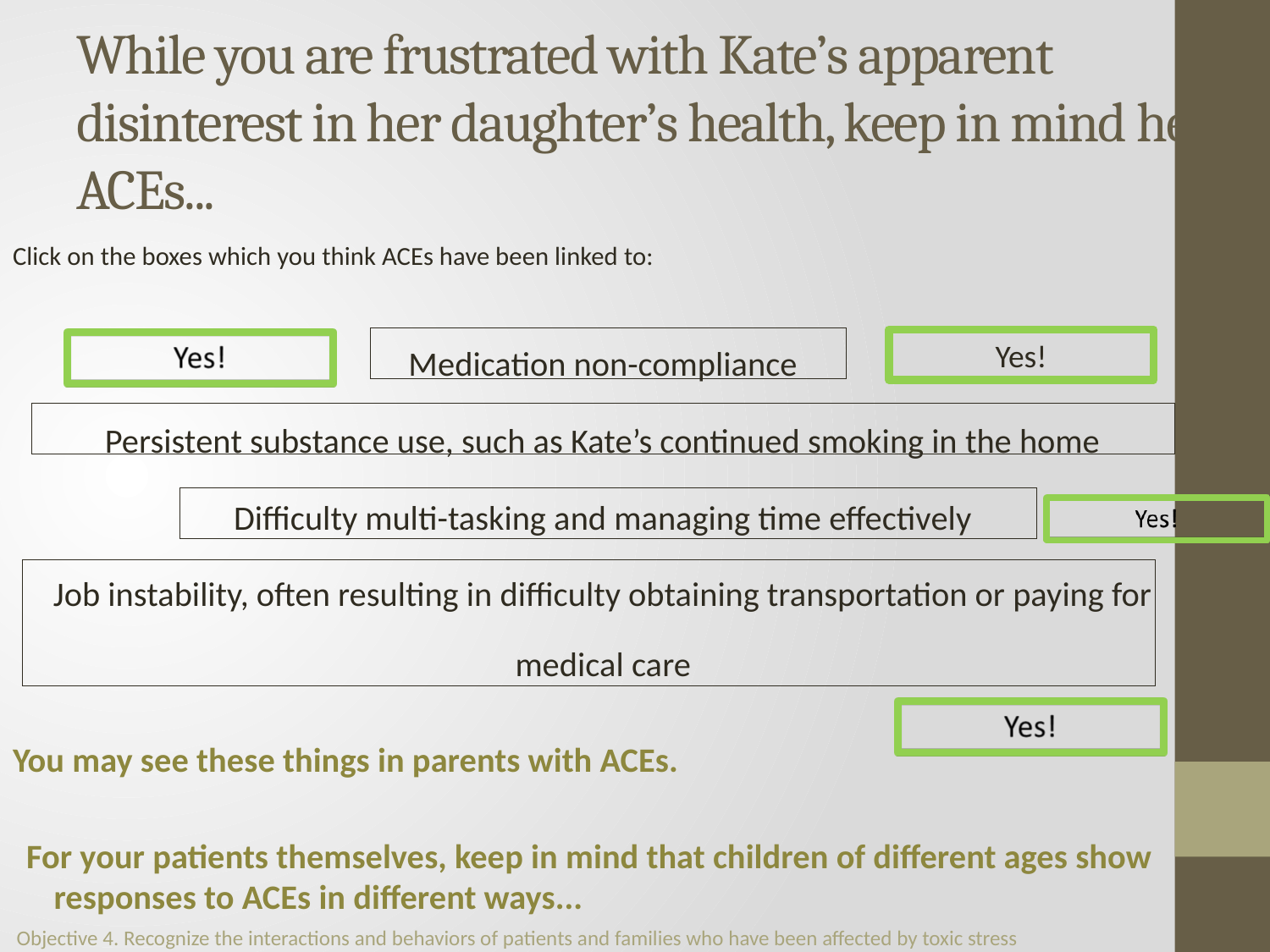

# While you are frustrated with Kate’s apparent disinterest in her daughter’s health, keep in mind her ACEs...
Click on the boxes which you think ACEs have been linked to:
Medication non-compliance
Persistent substance use, such as Kate’s continued smoking in the home
Difficulty multi-tasking and managing time effectively
Job instability, often resulting in difficulty obtaining transportation or paying for medical care
You may see these things in parents with ACEs.
For your patients themselves, keep in mind that children of different ages show responses to ACEs in different ways...
Yes!
Objective 4. Recognize the interactions and behaviors of patients and families who have been affected by toxic stress

## Slide 27
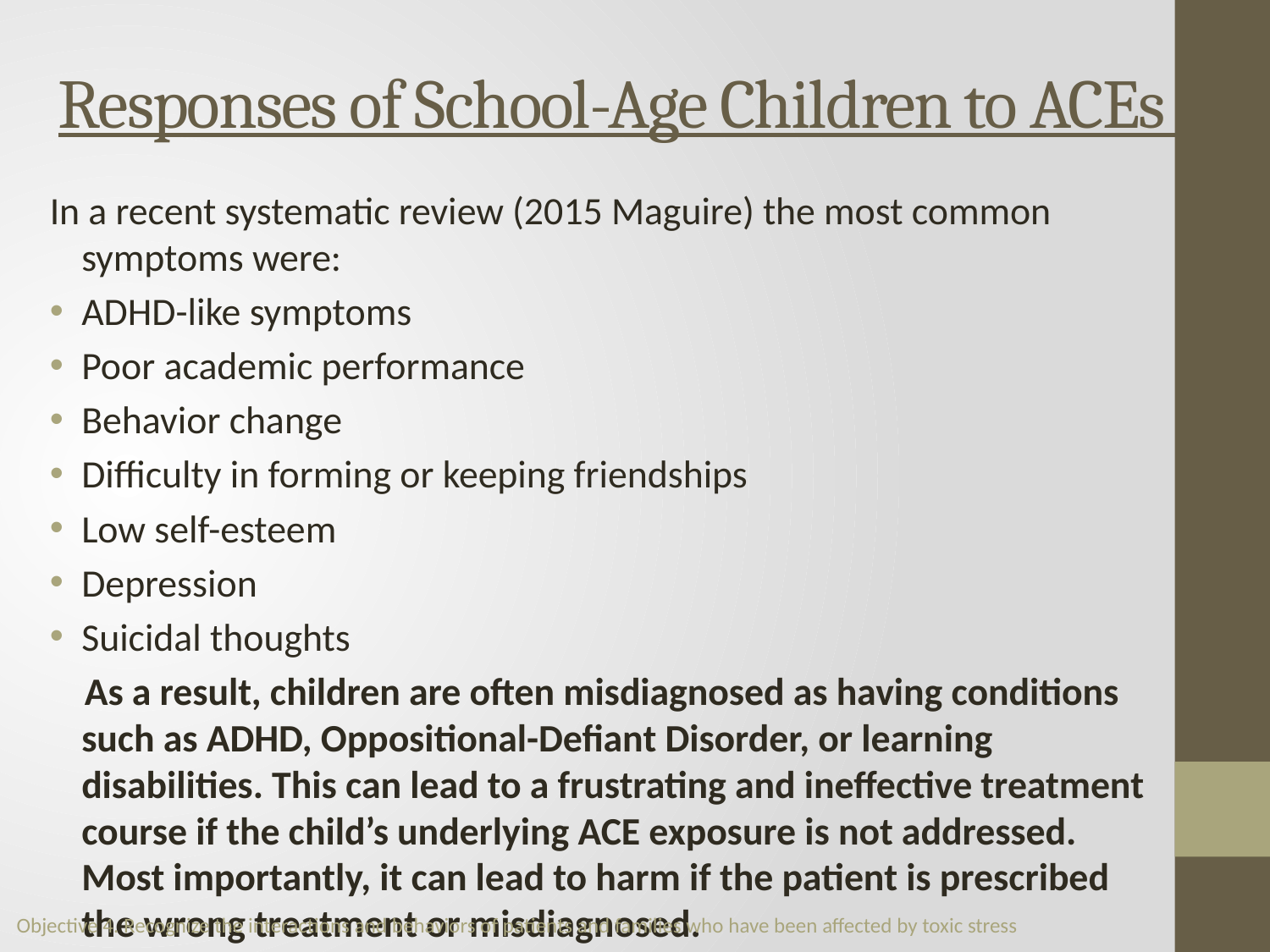

# Responses of School-Age Children to ACEs
In a recent systematic review (2015 Maguire) the most common symptoms were:
ADHD-like symptoms
Poor academic performance
Behavior change
Difficulty in forming or keeping friendships
Low self-esteem
Depression
Suicidal thoughts
 As a result, children are often misdiagnosed as having conditions such as ADHD, Oppositional-Defiant Disorder, or learning disabilities. This can lead to a frustrating and ineffective treatment course if the child’s underlying ACE exposure is not addressed. Most importantly, it can lead to harm if the patient is prescribed the wrong treatment or misdiagnosed.
Objective 4. Recognize the interactions and behaviors of patients and families who have been affected by toxic stress

## Slide 28
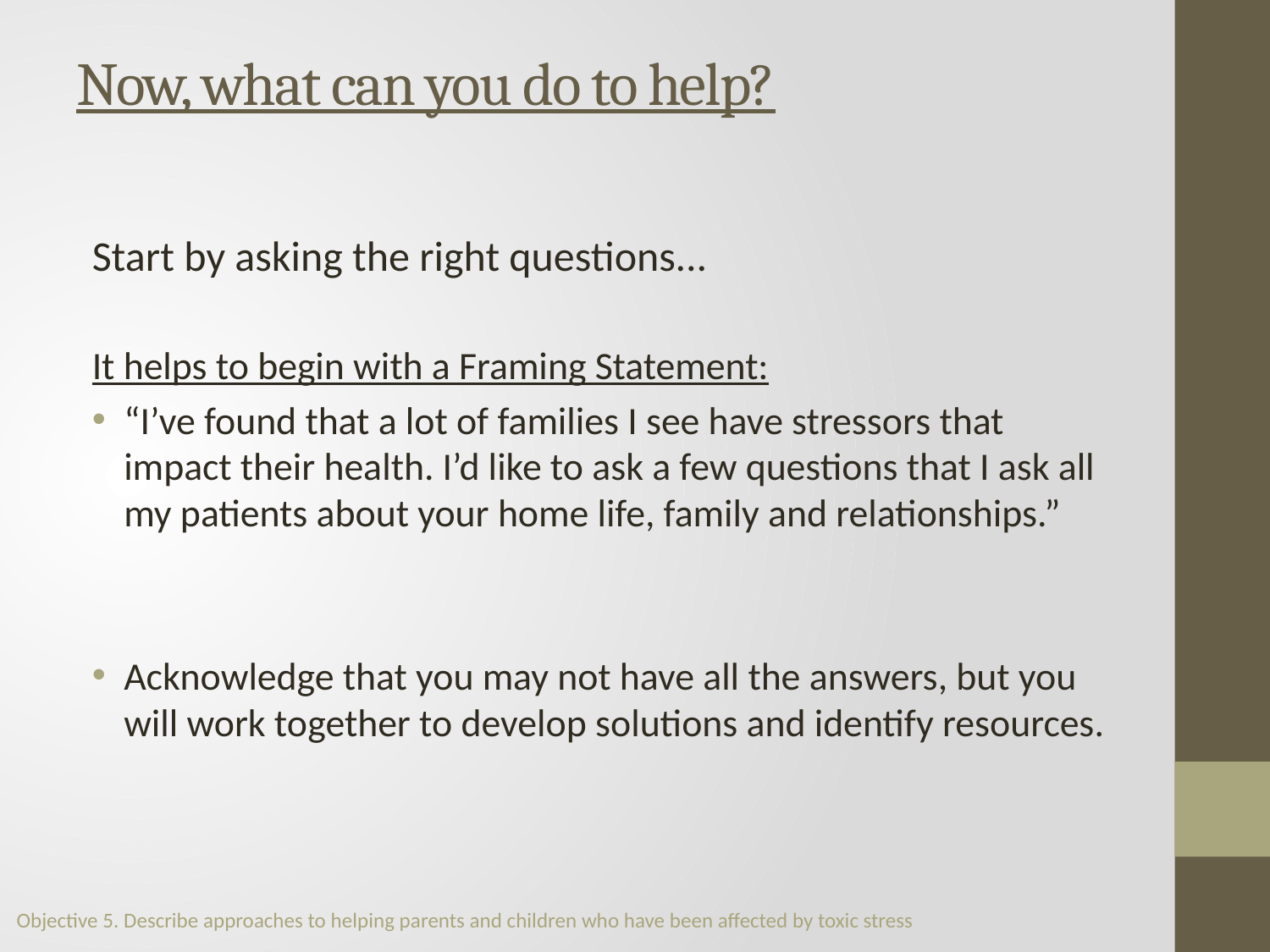

# Now, what can you do to help?
Start by asking the right questions...
It helps to begin with a Framing Statement:
“I’ve found that a lot of families I see have stressors that impact their health. I’d like to ask a few questions that I ask all my patients about your home life, family and relationships.”
Acknowledge that you may not have all the answers, but you will work together to develop solutions and identify resources.
Objective 5. Describe approaches to helping parents and children who have been affected by toxic stress

## Slide 29
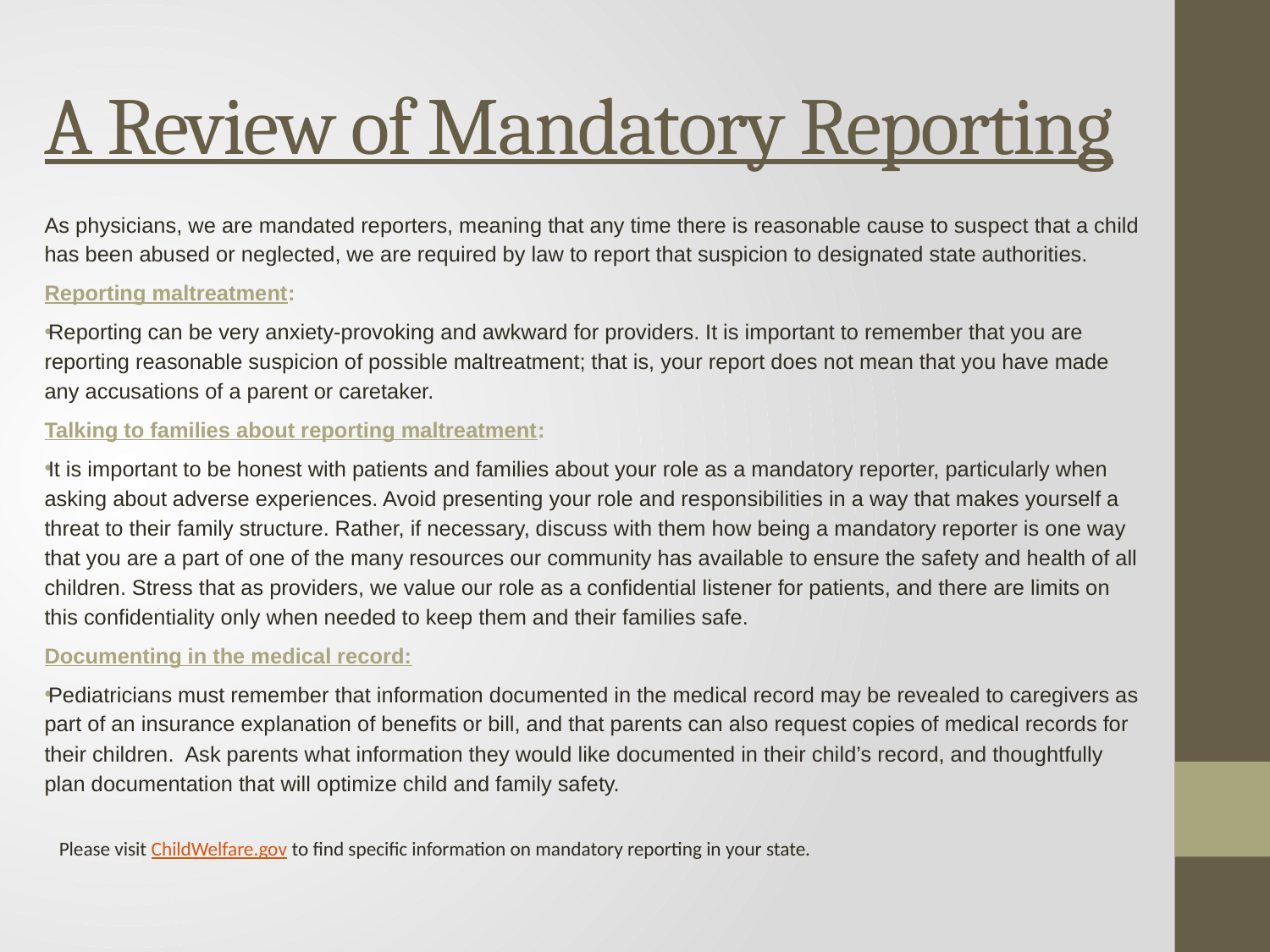

# A Review of Mandatory Reporting
As physicians, we are mandated reporters, meaning that any time there is reasonable cause to suspect that a child has been abused or neglected, we are required by law to report that suspicion to designated state authorities.
Reporting maltreatment:
Reporting can be very anxiety-provoking and awkward for providers. It is important to remember that you are reporting reasonable suspicion of possible maltreatment; that is, your report does not mean that you have made any accusations of a parent or caretaker.
Talking to families about reporting maltreatment:
It is important to be honest with patients and families about your role as a mandatory reporter, particularly when asking about adverse experiences. Avoid presenting your role and responsibilities in a way that makes yourself a threat to their family structure. Rather, if necessary, discuss with them how being a mandatory reporter is one way that you are a part of one of the many resources our community has available to ensure the safety and health of all children. Stress that as providers, we value our role as a confidential listener for patients, and there are limits on this confidentiality only when needed to keep them and their families safe.
Documenting in the medical record:
Pediatricians must remember that information documented in the medical record may be revealed to caregivers as part of an insurance explanation of benefits or bill, and that parents can also request copies of medical records for their children. Ask parents what information they would like documented in their child’s record, and thoughtfully plan documentation that will optimize child and family safety.
Please visit ChildWelfare.gov to find specific information on mandatory reporting in your state.

## Slide 30
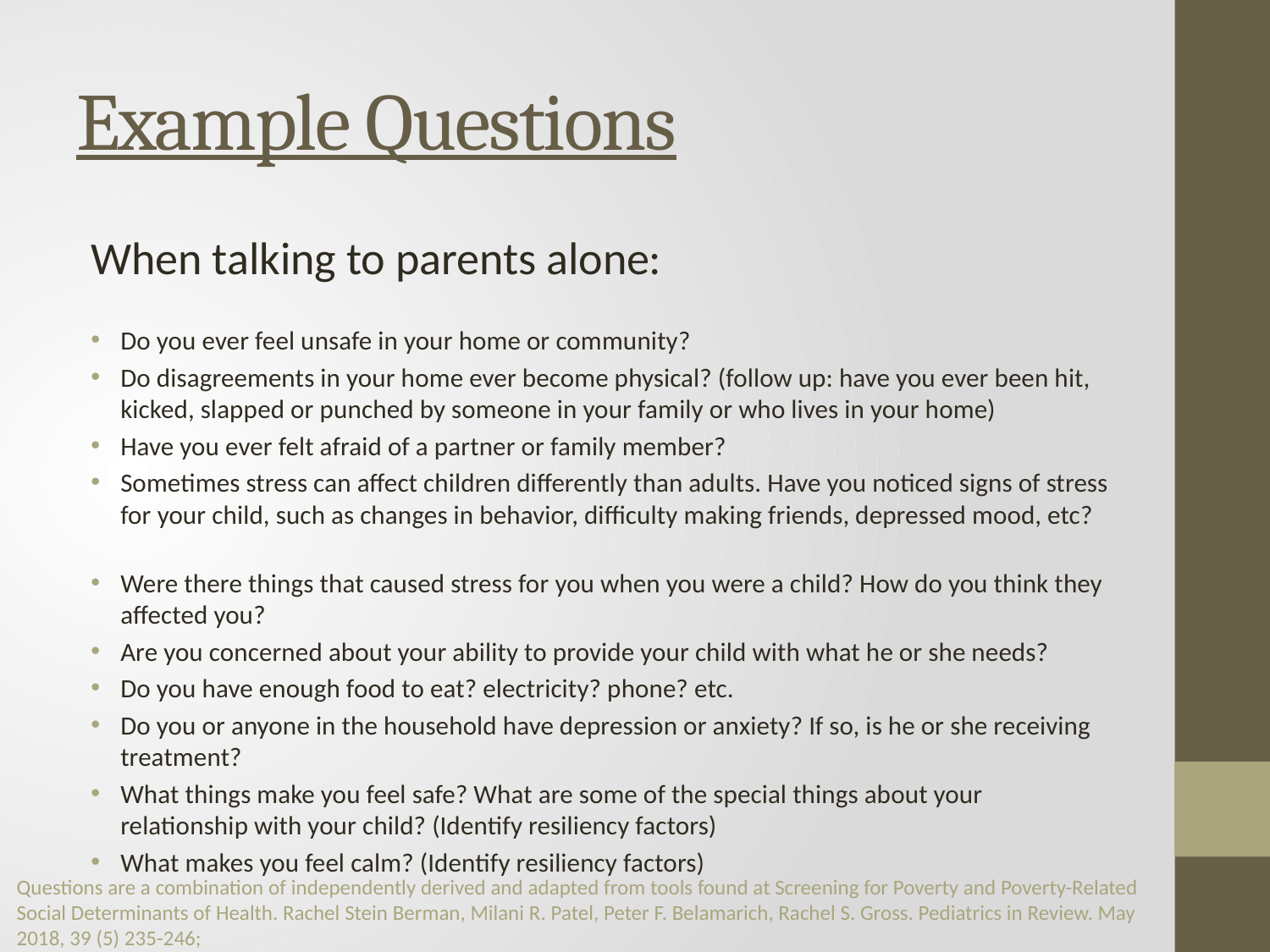

# Example Questions
When talking to parents alone:
Do you ever feel unsafe in your home or community?
Do disagreements in your home ever become physical? (follow up: have you ever been hit, kicked, slapped or punched by someone in your family or who lives in your home)
Have you ever felt afraid of a partner or family member?
Sometimes stress can affect children differently than adults. Have you noticed signs of stress for your child, such as changes in behavior, difficulty making friends, depressed mood, etc?
Were there things that caused stress for you when you were a child? How do you think they affected you?
Are you concerned about your ability to provide your child with what he or she needs?
Do you have enough food to eat? electricity? phone? etc.
Do you or anyone in the household have depression or anxiety? If so, is he or she receiving treatment?
What things make you feel safe? What are some of the special things about your relationship with your child? (Identify resiliency factors)
What makes you feel calm? (Identify resiliency factors)
Questions are a combination of independently derived and adapted from tools found at Screening for Poverty and Poverty-Related Social Determinants of Health. Rachel Stein Berman, Milani R. Patel, Peter F. Belamarich, Rachel S. Gross. Pediatrics in Review. May 2018, 39 (5) 235-246;

## Slide 31
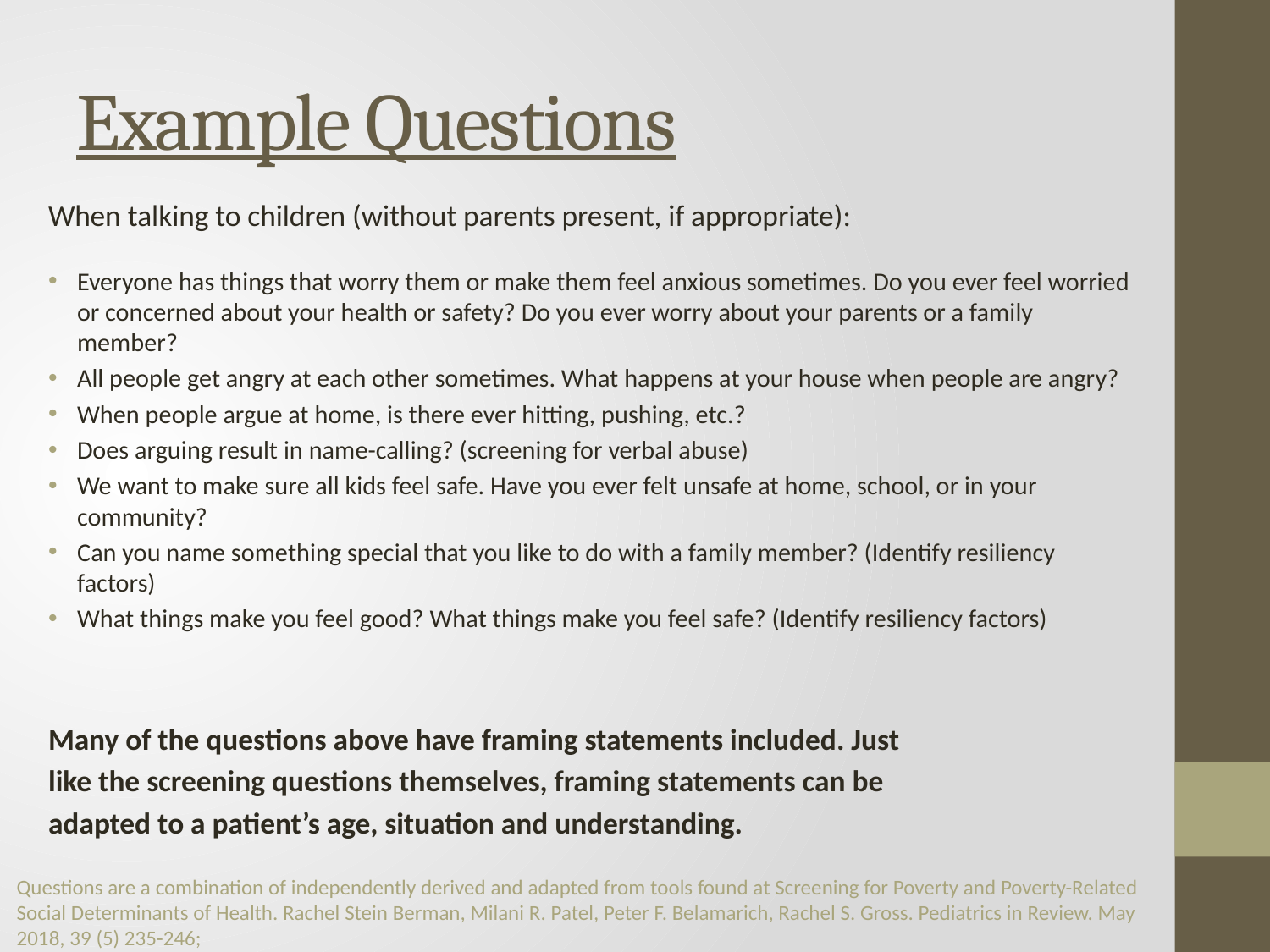

# Example Questions
When talking to children (without parents present, if appropriate):
Everyone has things that worry them or make them feel anxious sometimes. Do you ever feel worried or concerned about your health or safety? Do you ever worry about your parents or a family member?
All people get angry at each other sometimes. What happens at your house when people are angry?
When people argue at home, is there ever hitting, pushing, etc.?
Does arguing result in name-calling? (screening for verbal abuse)
We want to make sure all kids feel safe. Have you ever felt unsafe at home, school, or in your community?
Can you name something special that you like to do with a family member? (Identify resiliency factors)
What things make you feel good? What things make you feel safe? (Identify resiliency factors)
Many of the questions above have framing statements included. Just
like the screening questions themselves, framing statements can be
adapted to a patient’s age, situation and understanding.
Questions are a combination of independently derived and adapted from tools found at Screening for Poverty and Poverty-Related Social Determinants of Health. Rachel Stein Berman, Milani R. Patel, Peter F. Belamarich, Rachel S. Gross. Pediatrics in Review. May 2018, 39 (5) 235-246;

## Slide 32
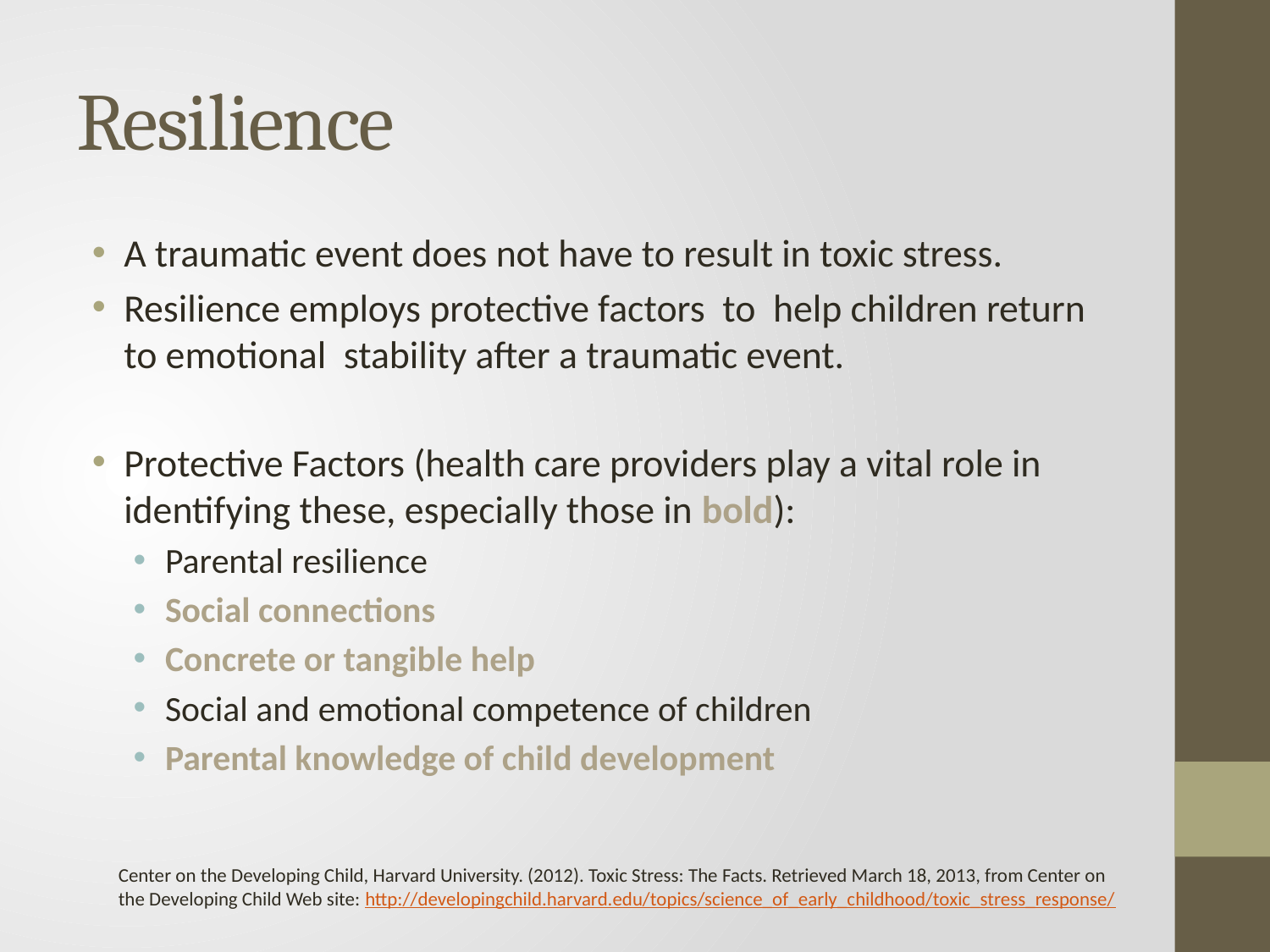

# Resilience
A traumatic event does not have to result in toxic stress.
Resilience employs protective factors to help children return to emotional stability after a traumatic event.
Protective Factors (health care providers play a vital role in identifying these, especially those in bold):
Parental resilience
Social connections
Concrete or tangible help
Social and emotional competence of children
Parental knowledge of child development
Center on the Developing Child, Harvard University. (2012). Toxic Stress: The Facts. Retrieved March 18, 2013, from Center on the Developing Child Web site: http://developingchild.harvard.edu/topics/science_of_early_childhood/toxic_stress_response/​

## Slide 33
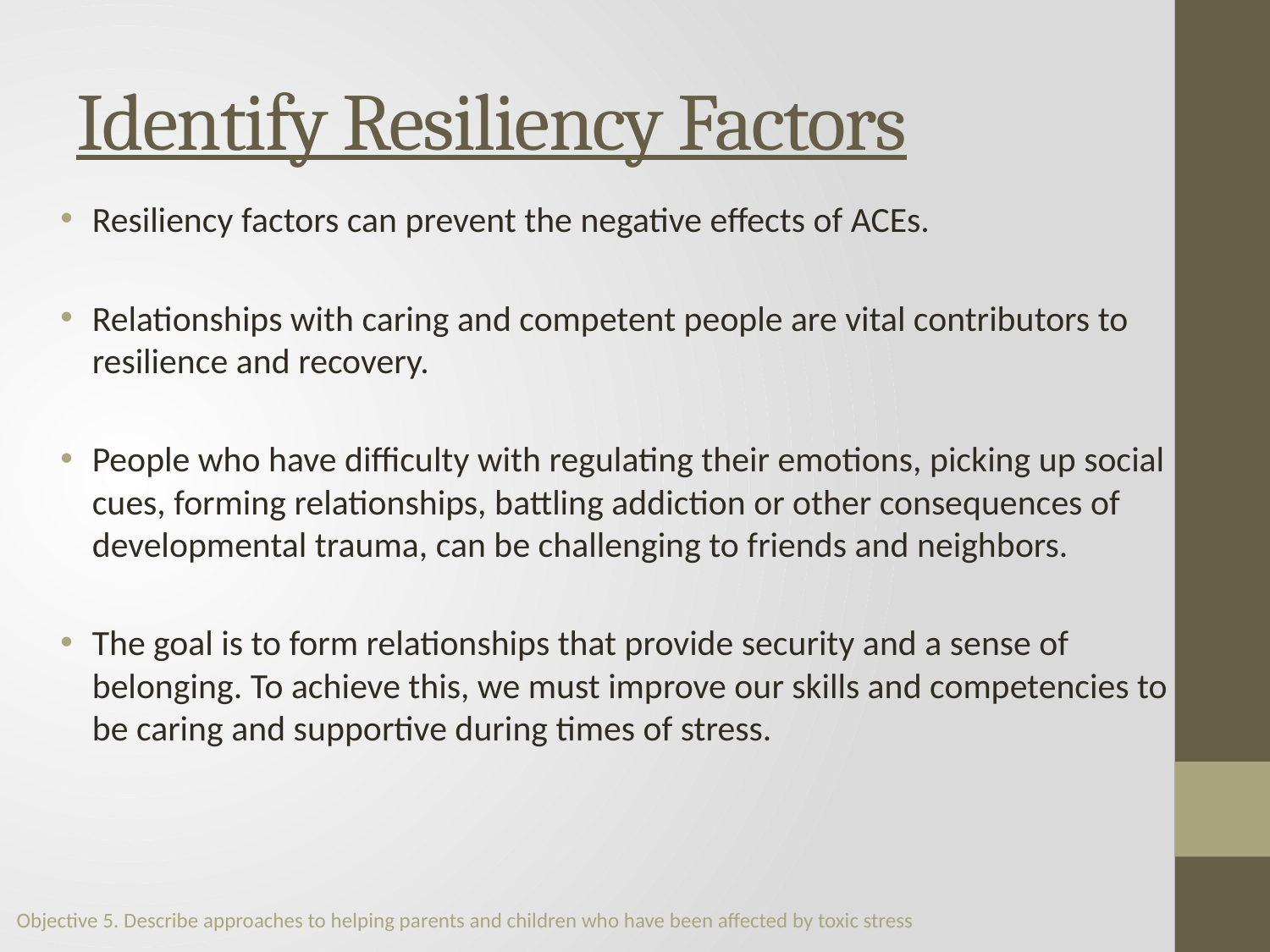

# Identify Resiliency Factors
Resiliency factors can prevent the negative effects of ACEs.
Relationships with caring and competent people are vital contributors to resilience and recovery.
People who have difficulty with regulating their emotions, picking up social cues, forming relationships, battling addiction or other consequences of developmental trauma, can be challenging to friends and neighbors.
The goal is to form relationships that provide security and a sense of belonging. To achieve this, we must improve our skills and competencies to be caring and supportive during times of stress.
Objective 5. Describe approaches to helping parents and children who have been affected by toxic stress

## Slide 34
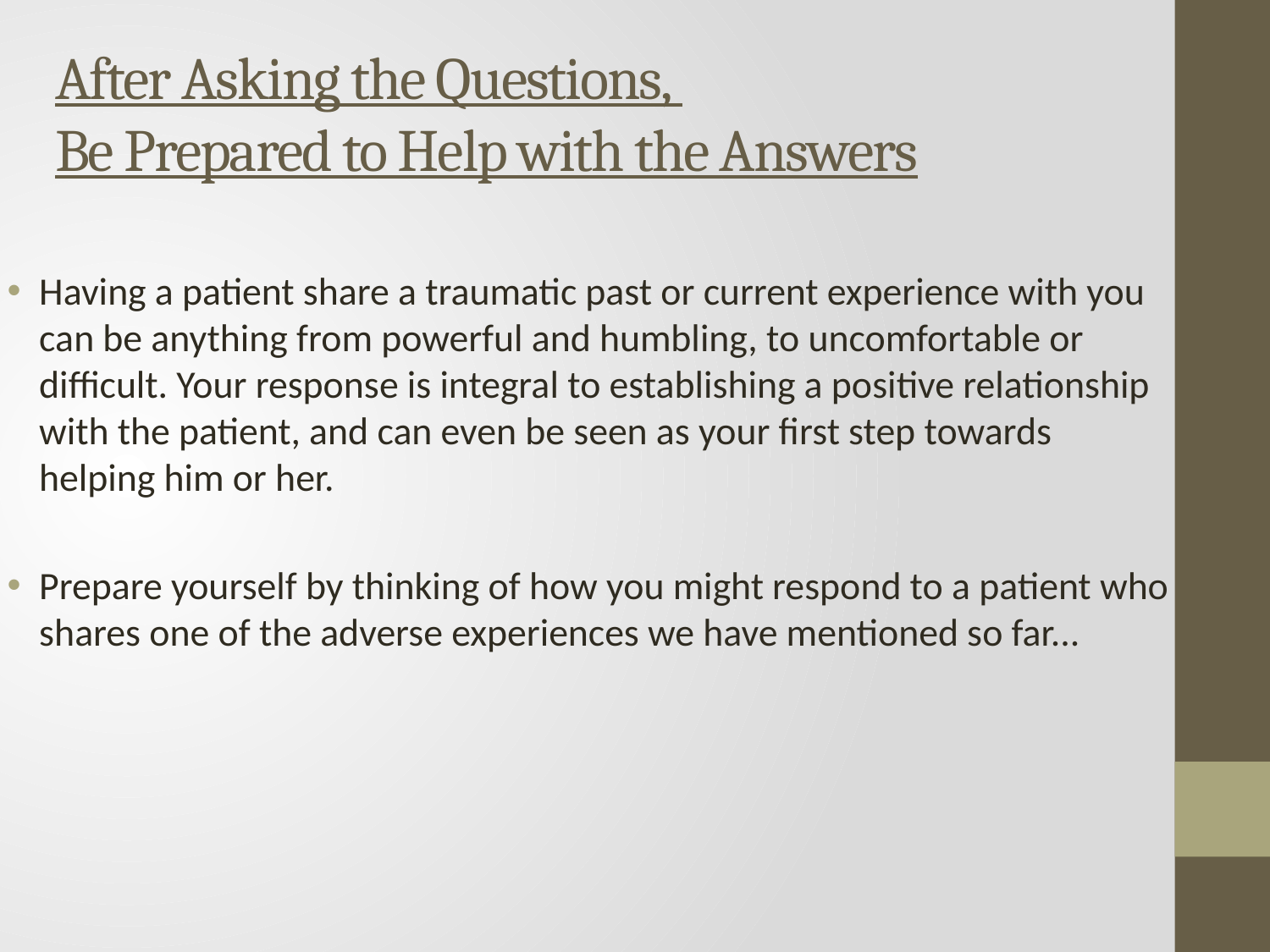

# After Asking the Questions, Be Prepared to Help with the Answers
Having a patient share a traumatic past or current experience with you can be anything from powerful and humbling, to uncomfortable or difficult. Your response is integral to establishing a positive relationship with the patient, and can even be seen as your first step towards helping him or her.
Prepare yourself by thinking of how you might respond to a patient who shares one of the adverse experiences we have mentioned so far...

## Slide 35
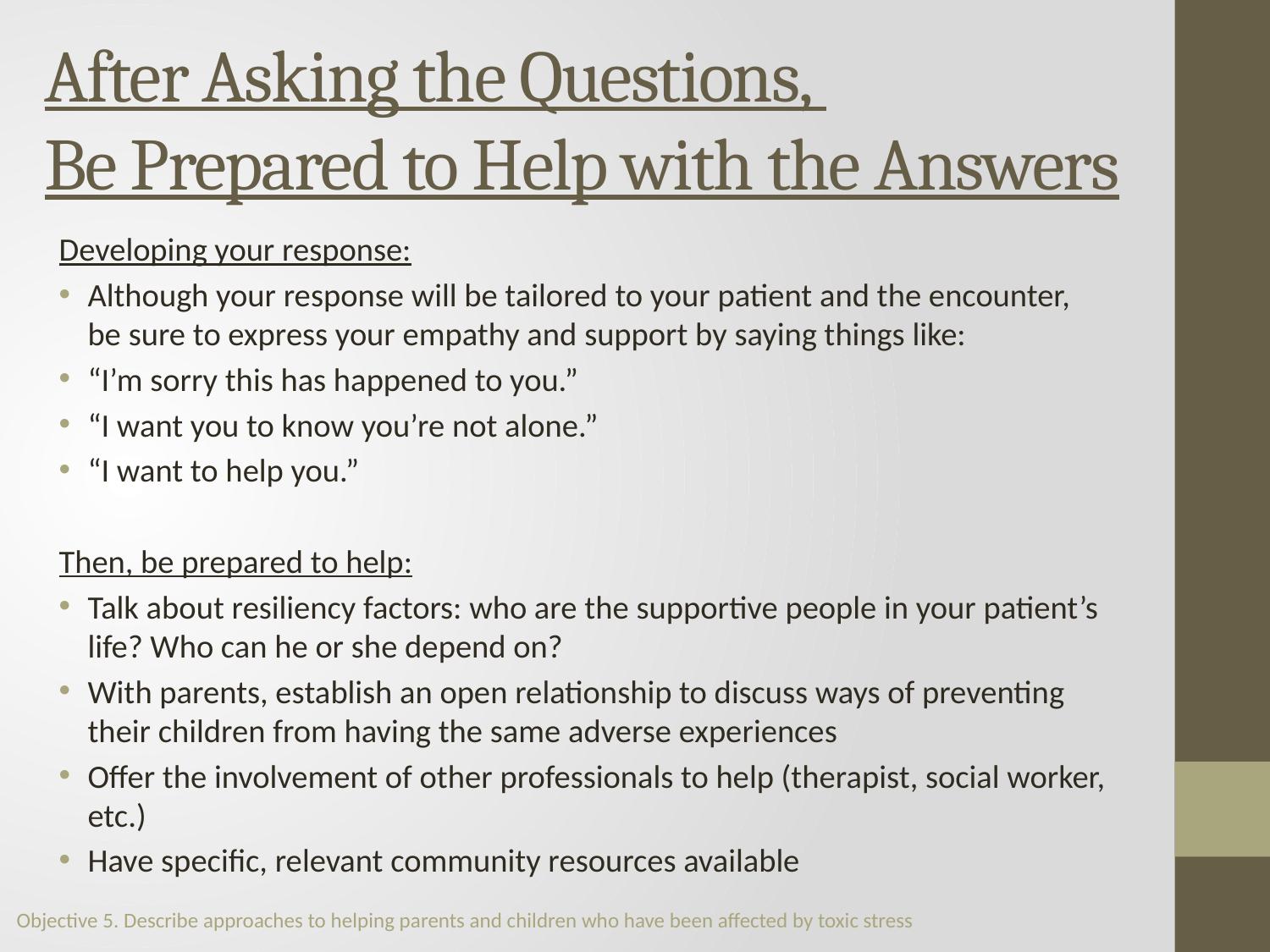

# After Asking the Questions, Be Prepared to Help with the Answers
Developing your response:
Although your response will be tailored to your patient and the encounter, be sure to express your empathy and support by saying things like:
“I’m sorry this has happened to you.”
“I want you to know you’re not alone.”
“I want to help you.”
Then, be prepared to help:
Talk about resiliency factors: who are the supportive people in your patient’s life? Who can he or she depend on?
With parents, establish an open relationship to discuss ways of preventing their children from having the same adverse experiences
Offer the involvement of other professionals to help (therapist, social worker, etc.)
Have specific, relevant community resources available
Objective 5. Describe approaches to helping parents and children who have been affected by toxic stress

## Slide 36
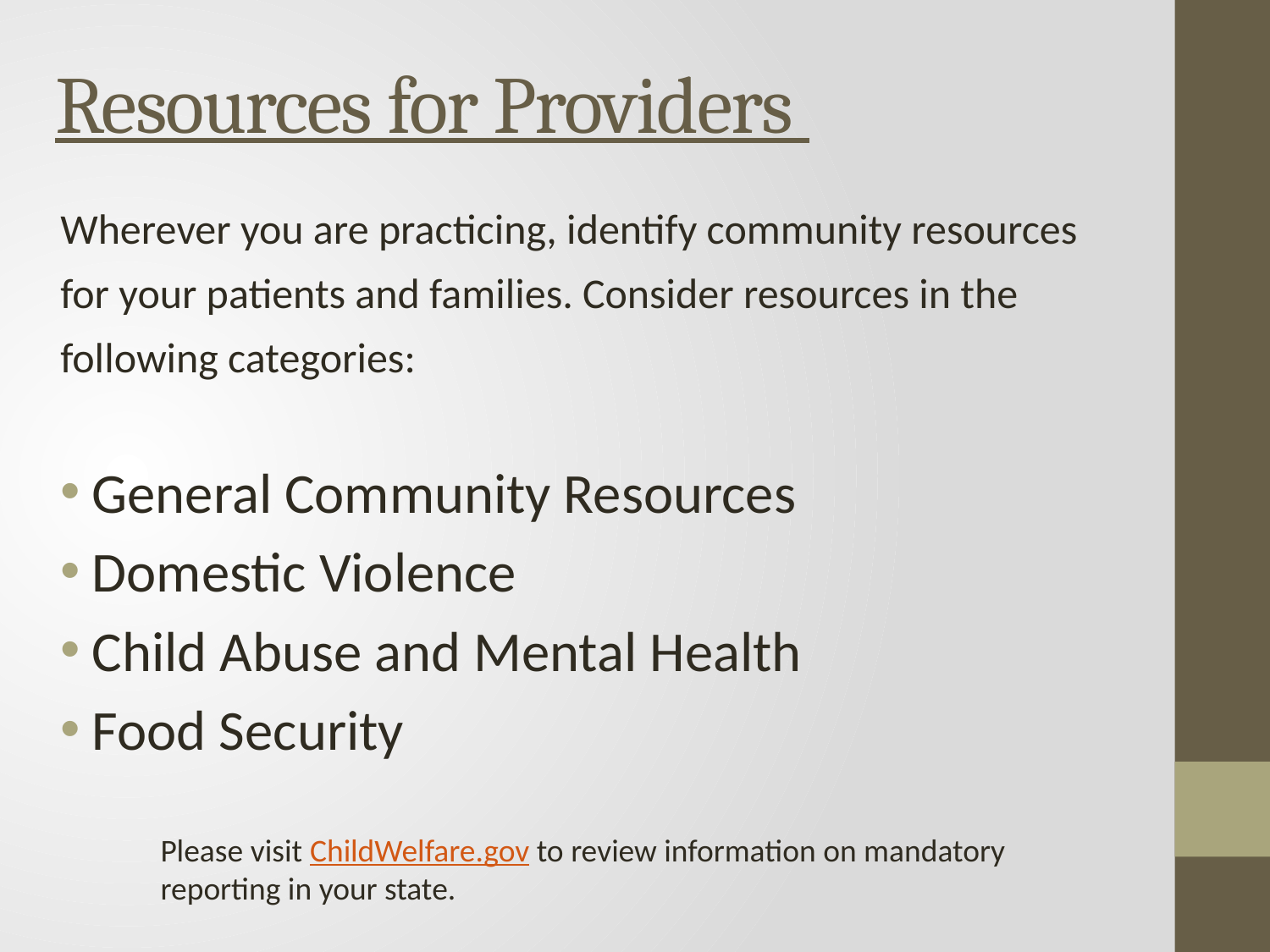

# Resources for Providers
Wherever you are practicing, identify community resources
for your patients and families. Consider resources in the
following categories:
General Community Resources
Domestic Violence
Child Abuse and Mental Health
Food Security
Please visit ChildWelfare.gov to review information on mandatory reporting in your state.

## Slide 37
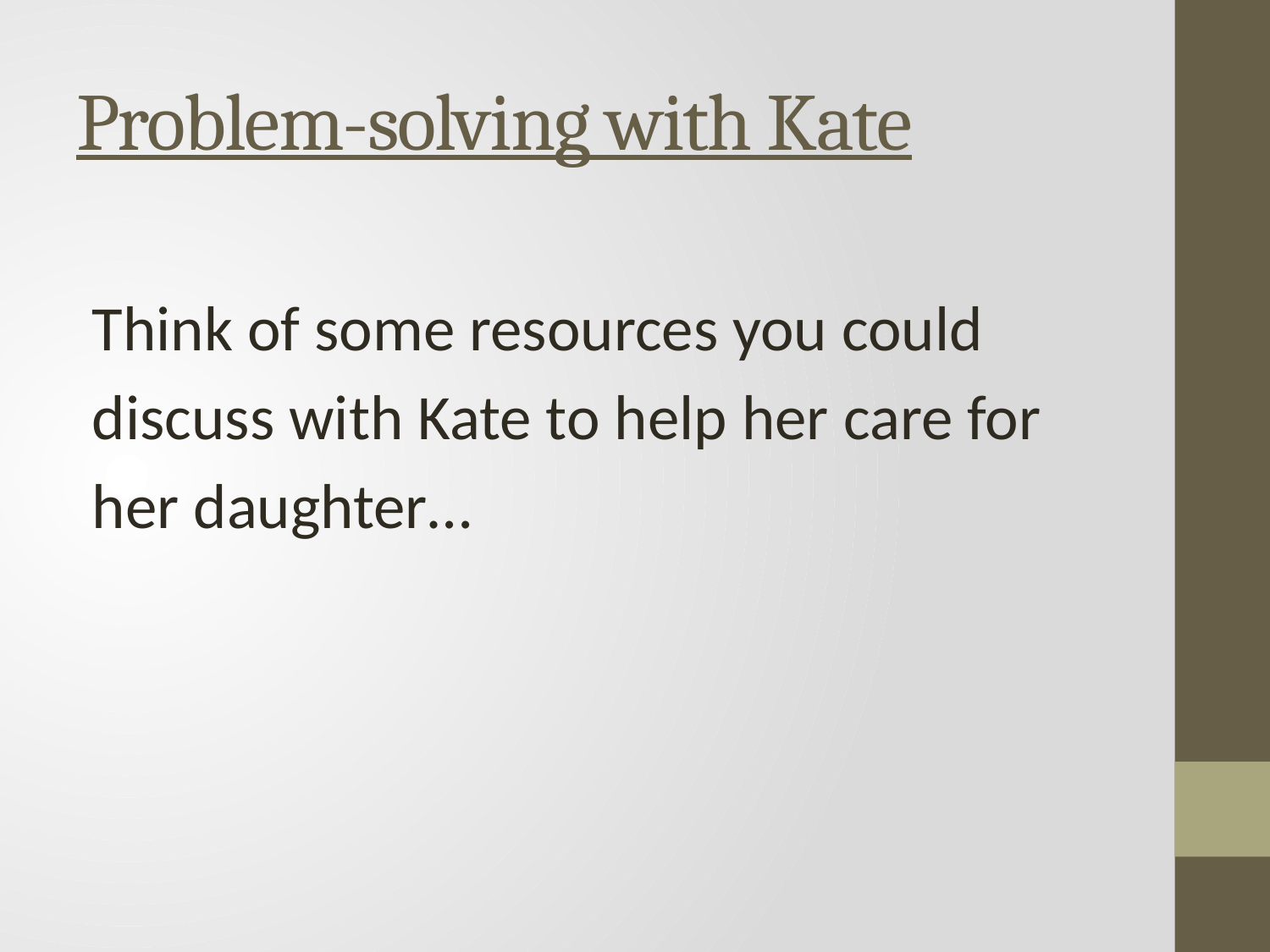

# Problem-solving with Kate
Think of some resources you could
discuss with Kate to help her care for
her daughter…

## Slide 38
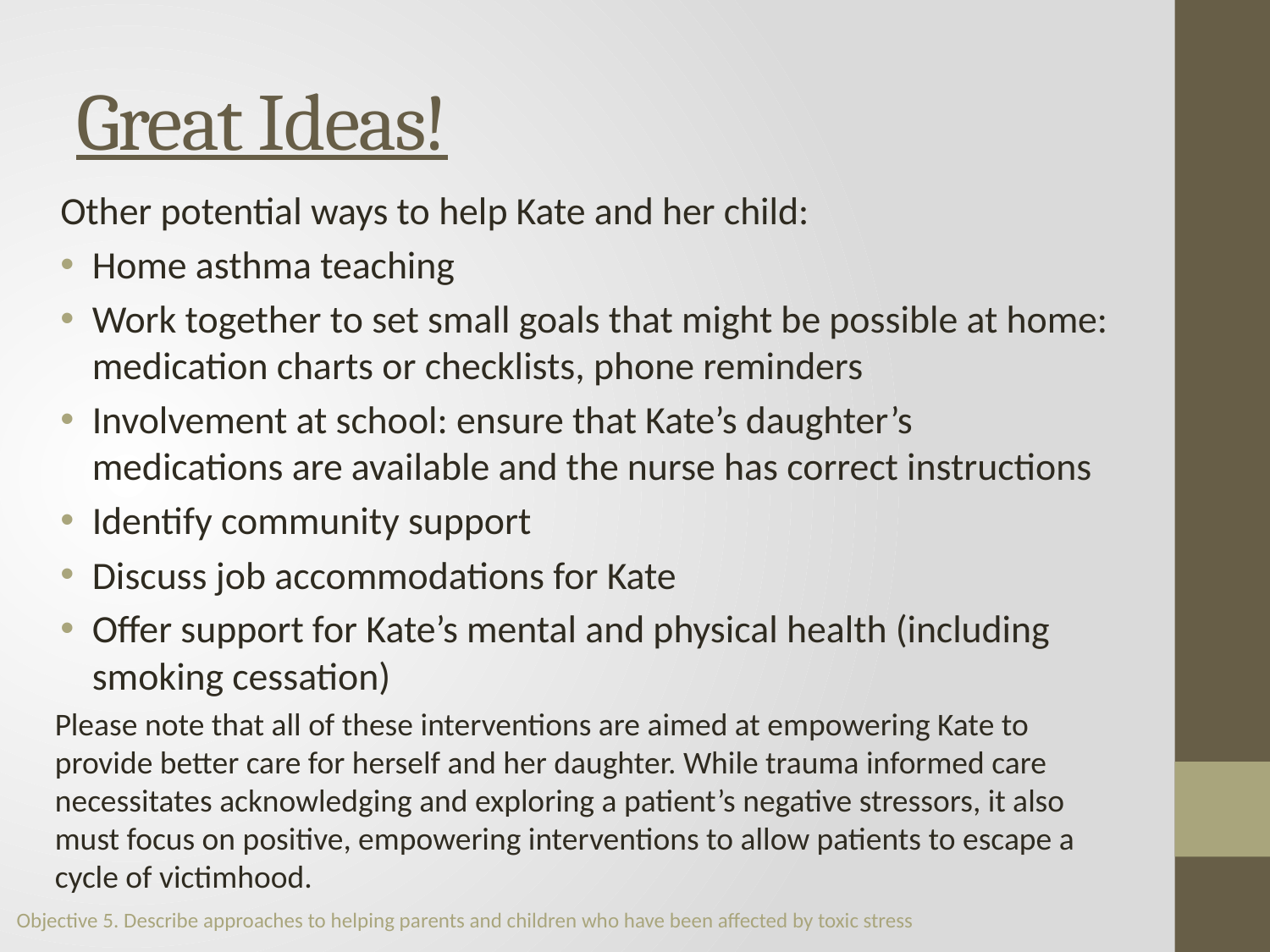

# Great Ideas!
Other potential ways to help Kate and her child:
Home asthma teaching
Work together to set small goals that might be possible at home: medication charts or checklists, phone reminders
Involvement at school: ensure that Kate’s daughter’s medications are available and the nurse has correct instructions
Identify community support
Discuss job accommodations for Kate
Offer support for Kate’s mental and physical health (including smoking cessation)
Please note that all of these interventions are aimed at empowering Kate to provide better care for herself and her daughter. While trauma informed care necessitates acknowledging and exploring a patient’s negative stressors, it also must focus on positive, empowering interventions to allow patients to escape a cycle of victimhood.
Objective 5. Describe approaches to helping parents and children who have been affected by toxic stress

## Slide 39
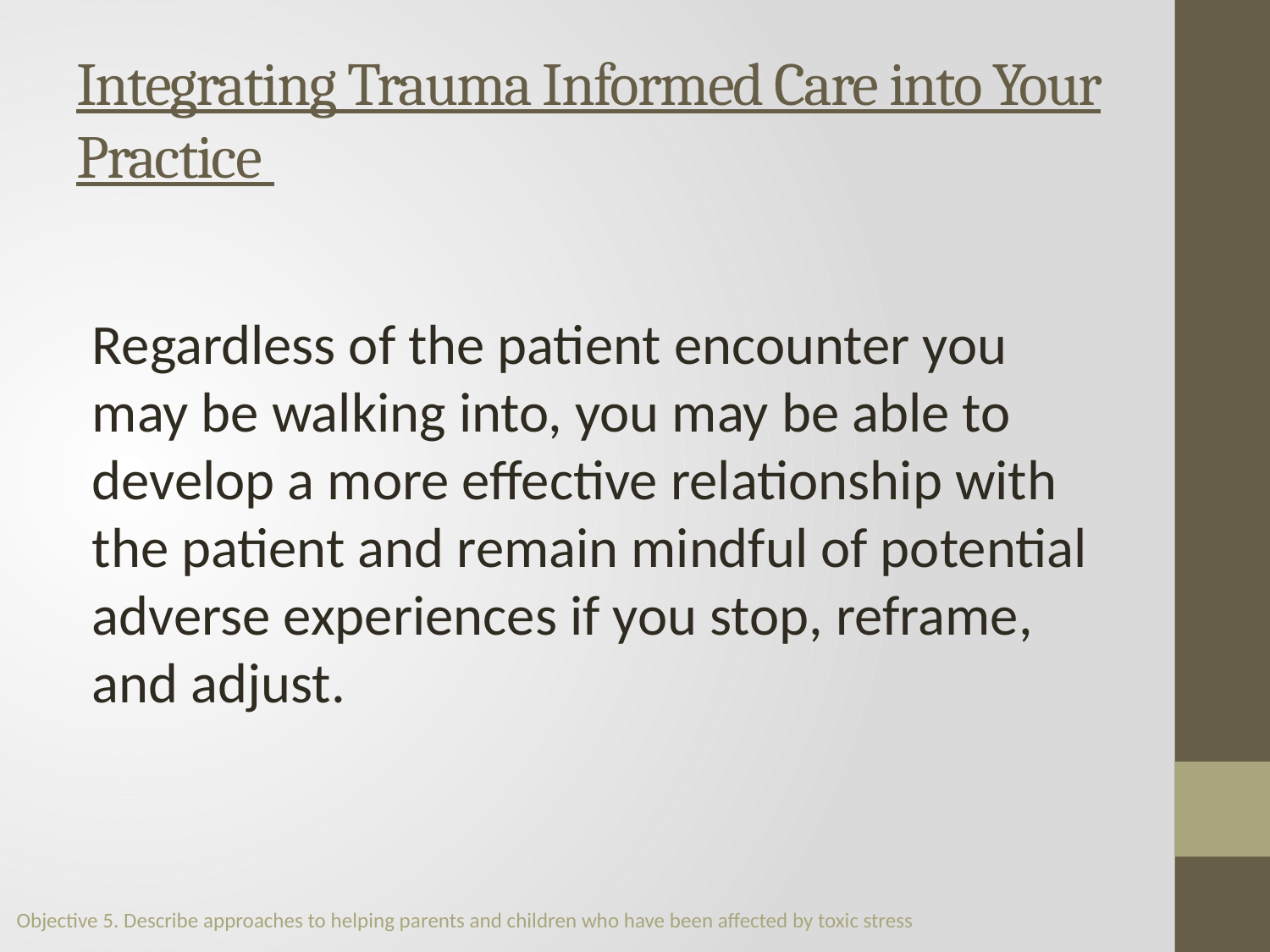

# Integrating Trauma Informed Care into Your Practice
Regardless of the patient encounter you may be walking into, you may be able to develop a more effective relationship with the patient and remain mindful of potential adverse experiences if you stop, reframe, and adjust.
Objective 5. Describe approaches to helping parents and children who have been affected by toxic stress

## Slide 40
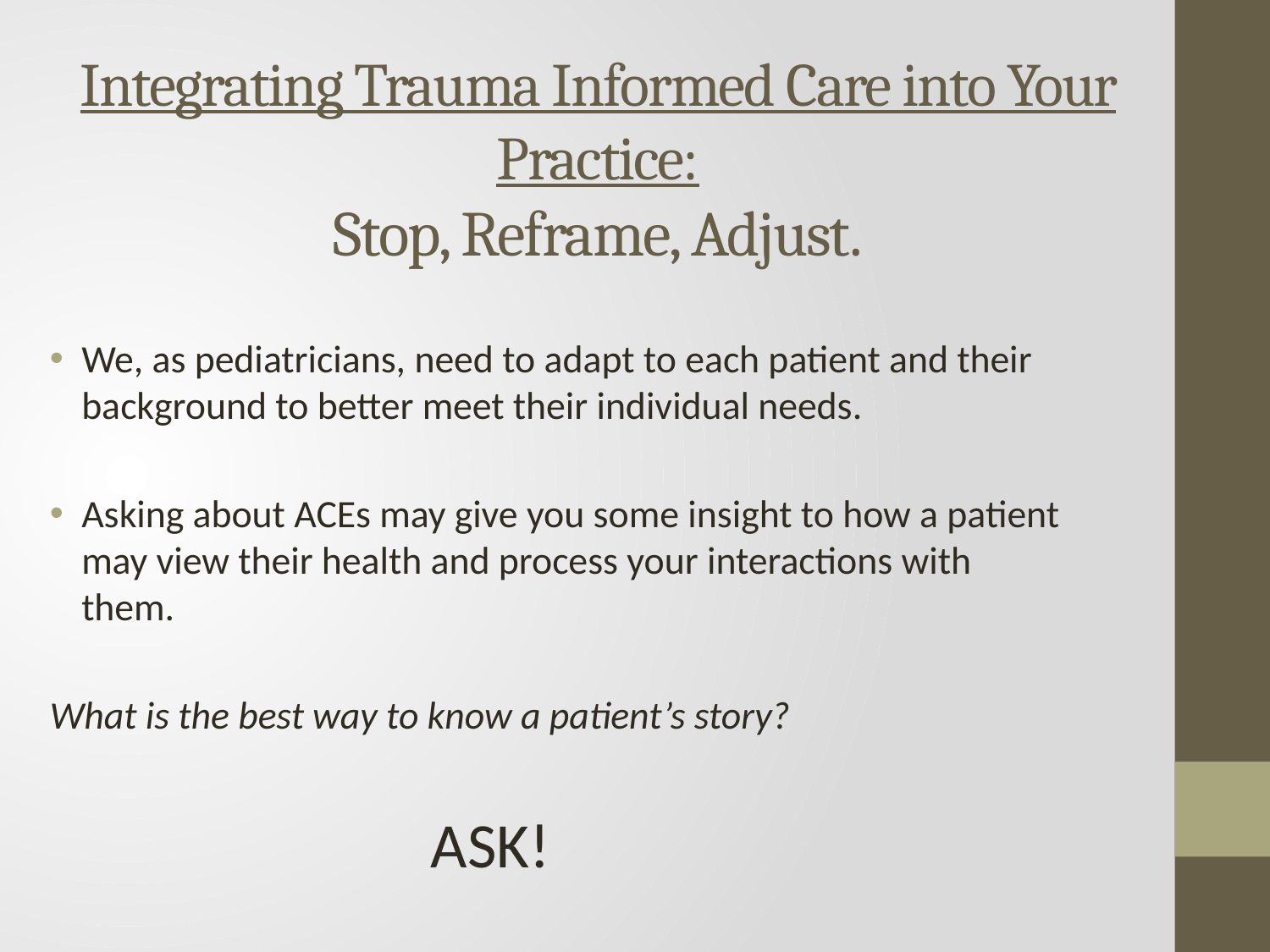

# Integrating Trauma Informed Care into Your Practice: Stop, Reframe, Adjust.
We, as pediatricians, need to adapt to each patient and their background to better meet their individual needs.
Asking about ACEs may give you some insight to how a patient may view their health and process your interactions with them.
What is the best way to know a patient’s story?
			ASK!

## Slide 41
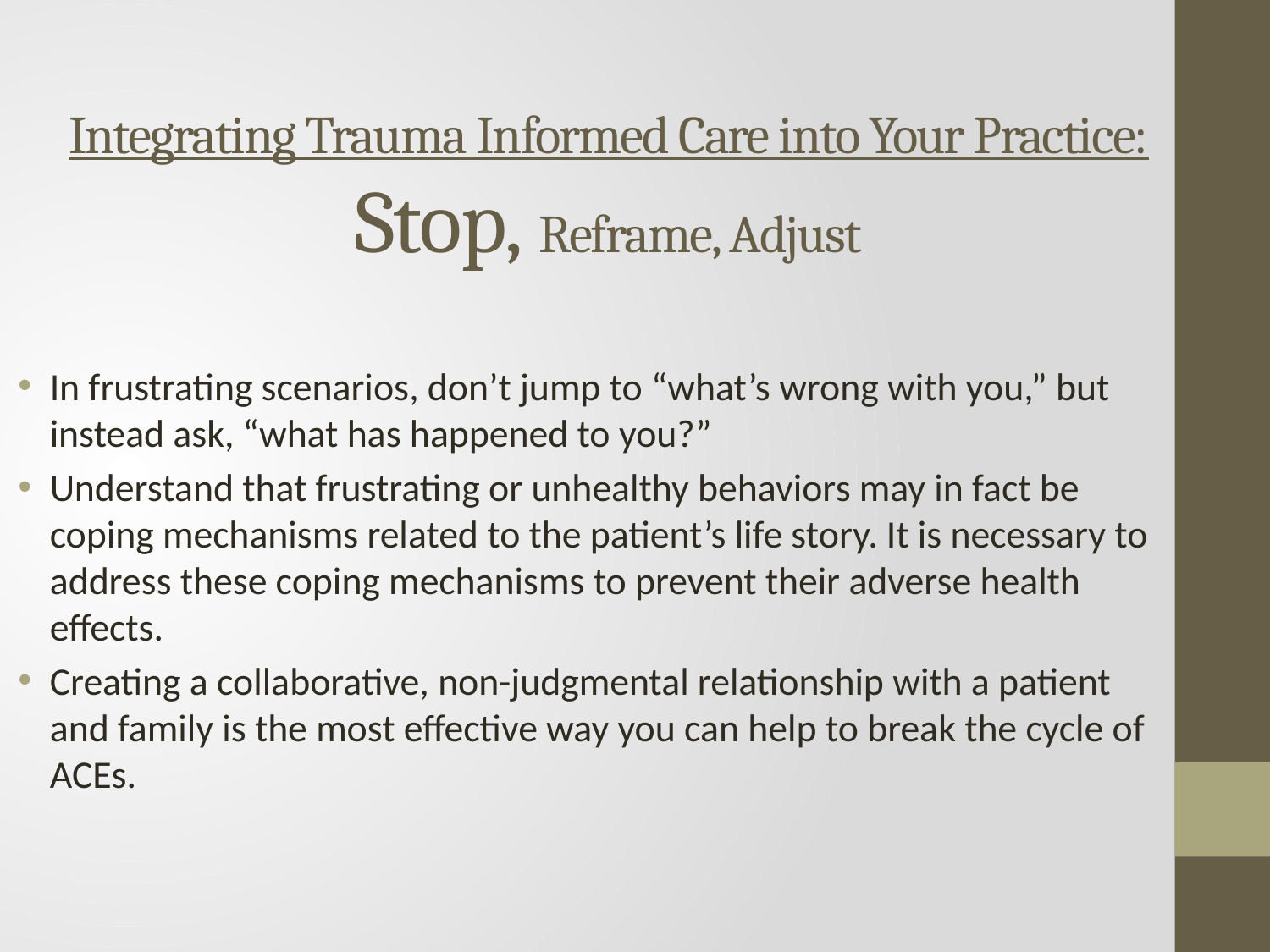

# Integrating Trauma Informed Care into Your Practice: Stop, Reframe, Adjust
In frustrating scenarios, don’t jump to “what’s wrong with you,” but instead ask, “what has happened to you?”
Understand that frustrating or unhealthy behaviors may in fact be coping mechanisms related to the patient’s life story. It is necessary to address these coping mechanisms to prevent their adverse health effects.
Creating a collaborative, non-judgmental relationship with a patient and family is the most effective way you can help to break the cycle of ACEs.

## Slide 42
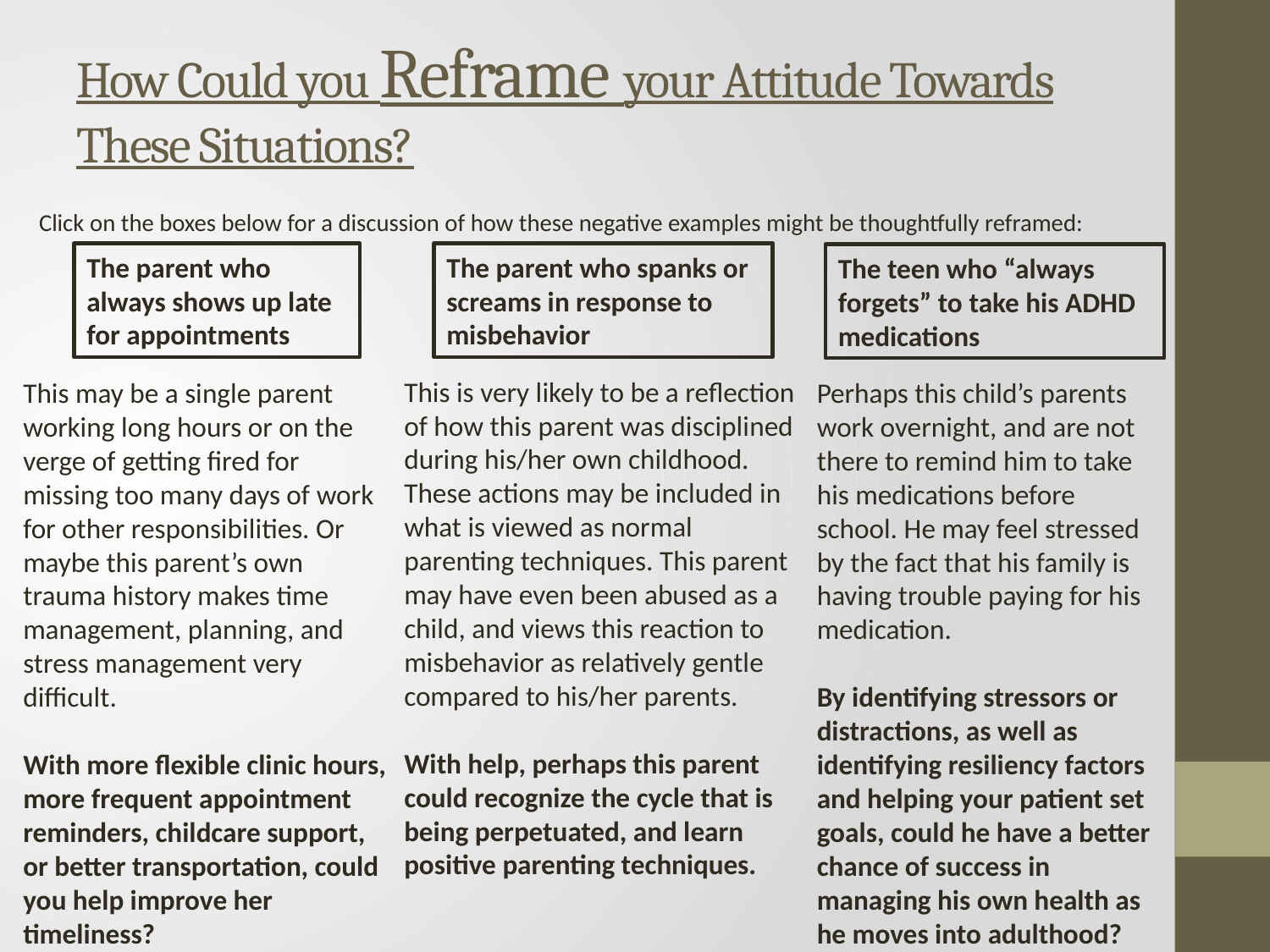

# How Could you Reframe your Attitude Towards These Situations?
Click on the boxes below for a discussion of how these negative examples might be thoughtfully reframed:
The parent who always shows up late for appointments
The parent who spanks or screams in response to misbehavior
The teen who “always forgets” to take his ADHD medications
This is very likely to be a reflection of how this parent was disciplined during his/her own childhood. These actions may be included in what is viewed as normal parenting techniques. This parent may have even been abused as a child, and views this reaction to misbehavior as relatively gentle compared to his/her parents.
With help, perhaps this parent could recognize the cycle that is being perpetuated, and learn positive parenting techniques.
This may be a single parent working long hours or on the verge of getting fired for missing too many days of work for other responsibilities. Or maybe this parent’s own trauma history makes time management, planning, and stress management very difficult.
With more flexible clinic hours, more frequent appointment reminders, childcare support, or better transportation, could you help improve her timeliness?
Perhaps this child’s parents work overnight, and are not there to remind him to take his medications before school. He may feel stressed by the fact that his family is having trouble paying for his medication.
By identifying stressors or distractions, as well as identifying resiliency factors and helping your patient set goals, could he have a better chance of success in managing his own health as he moves into adulthood?

## Slide 43
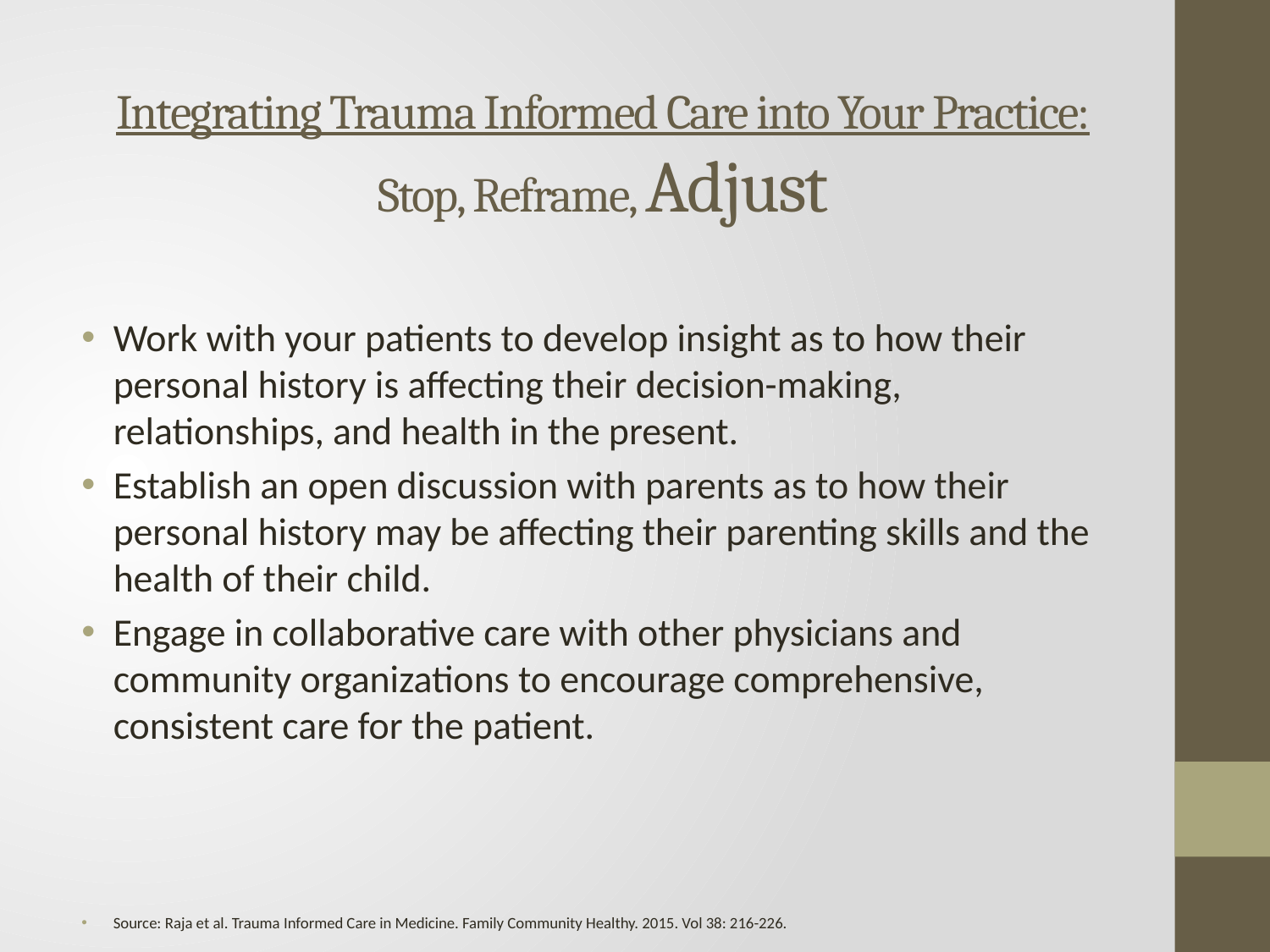

# Integrating Trauma Informed Care into Your Practice: Stop, Reframe, Adjust
Work with your patients to develop insight as to how their personal history is affecting their decision-making, relationships, and health in the present.
Establish an open discussion with parents as to how their personal history may be affecting their parenting skills and the health of their child.
Engage in collaborative care with other physicians and community organizations to encourage comprehensive, consistent care for the patient.
Source: Raja et al. Trauma Informed Care in Medicine. Family Community Healthy. 2015. Vol 38: 216-226.

## Slide 44
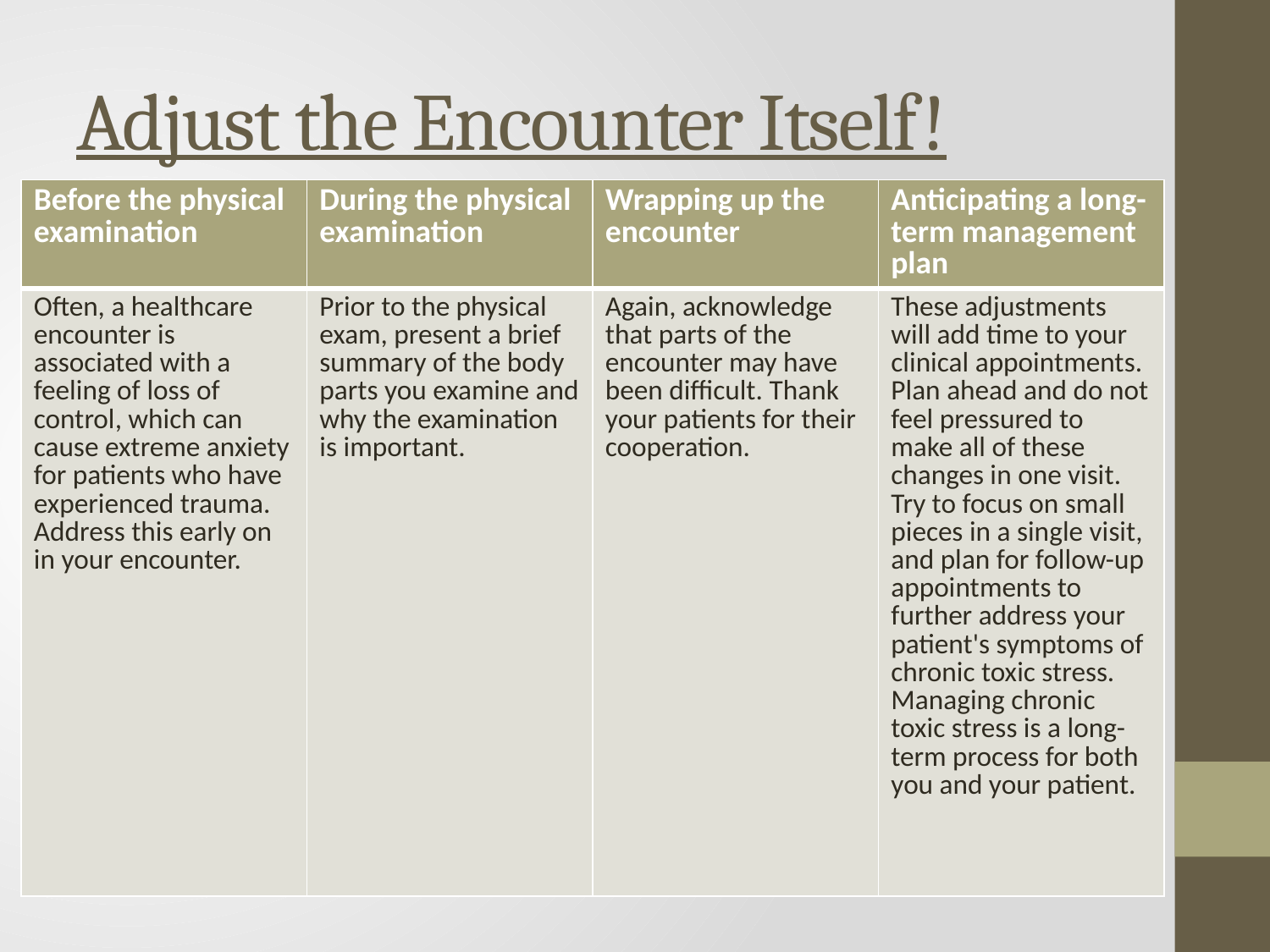

# Adjust the Encounter Itself!
| Before the physical examination | During the physical examination | Wrapping up the encounter | Anticipating a long-term management plan |
| --- | --- | --- | --- |
| Often, a healthcare encounter is associated with a feeling of loss of control, which can cause extreme anxiety for patients who have experienced trauma. Address this early on in your encounter. | Prior to the physical exam, present a brief summary of the body parts you examine and why the examination is important. | Again, acknowledge that parts of the encounter may have been difficult. Thank your patients for their cooperation. | These adjustments will add time to your clinical appointments. Plan ahead and do not feel pressured to make all of these changes in one visit. Try to focus on small pieces in a single visit, and plan for follow-up appointments to further address your patient's symptoms of chronic toxic stress. Managing chronic toxic stress is a long-term process for both you and your patient. |

## Slide 45
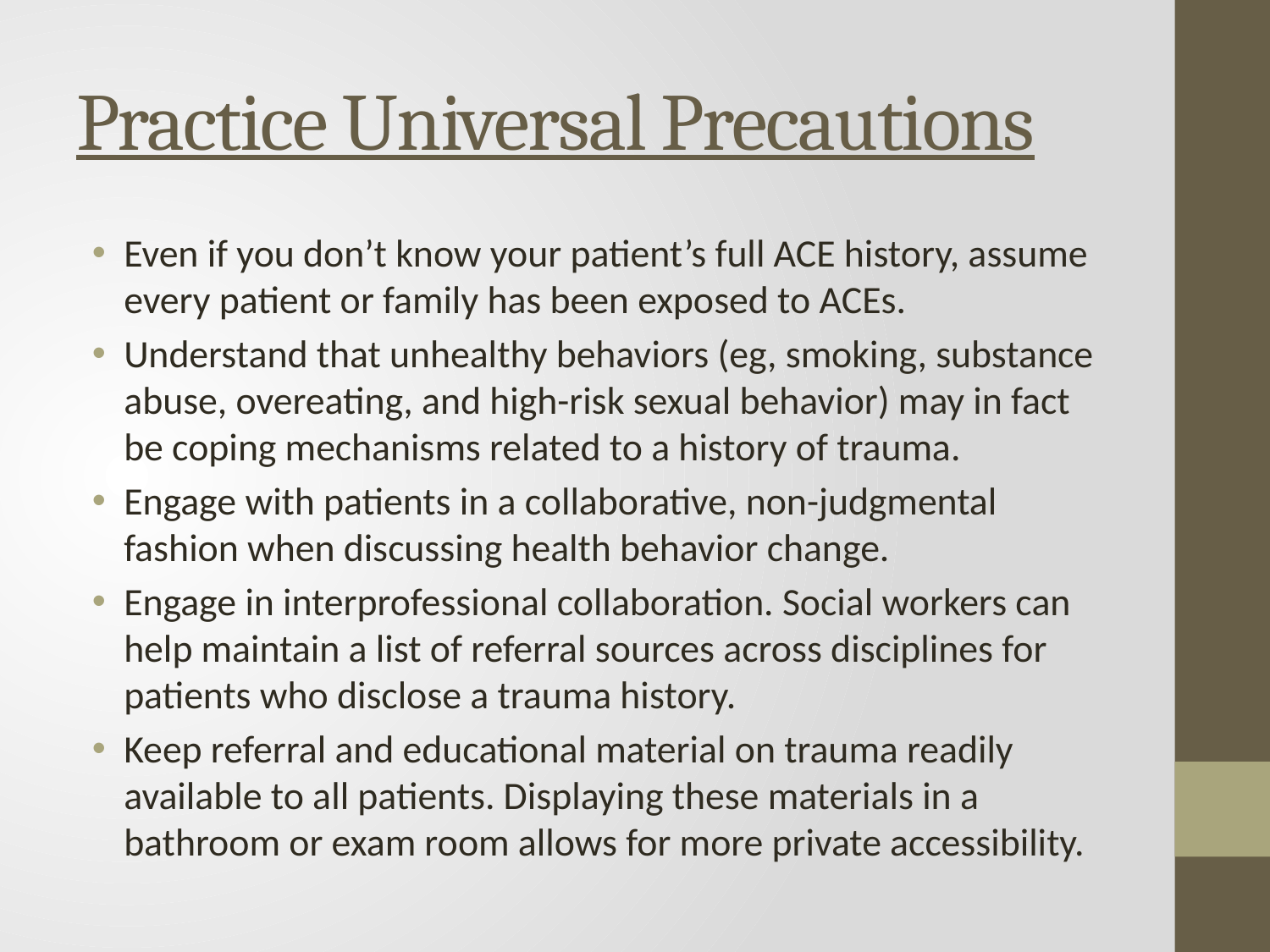

# Practice Universal Precautions
Even if you don’t know your patient’s full ACE history, assume every patient or family has been exposed to ACEs.
Understand that unhealthy behaviors (eg, smoking, substance abuse, overeating, and high-risk sexual behavior) may in fact be coping mechanisms related to a history of trauma.
Engage with patients in a collaborative, non-judgmental fashion when discussing health behavior change.
Engage in interprofessional collaboration. Social workers can help maintain a list of referral sources across disciplines for patients who disclose a trauma history.
Keep referral and educational material on trauma readily available to all patients. Displaying these materials in a bathroom or exam room allows for more private accessibility.

## Slide 46
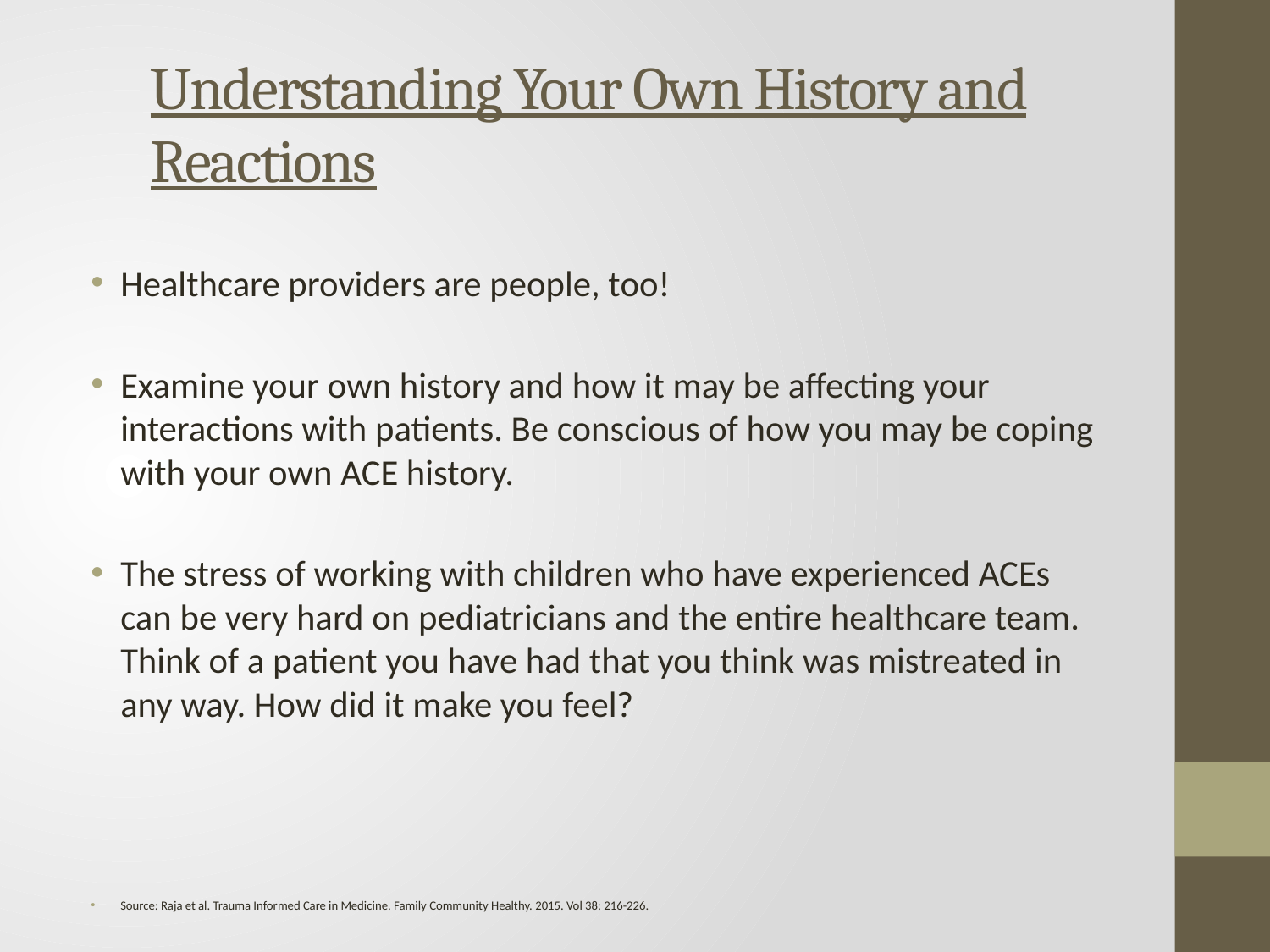

# Understanding Your Own History and Reactions
Healthcare providers are people, too!
Examine your own history and how it may be affecting your interactions with patients. Be conscious of how you may be coping with your own ACE history.
The stress of working with children who have experienced ACEs can be very hard on pediatricians and the entire healthcare team. Think of a patient you have had that you think was mistreated in any way. How did it make you feel?
Source: Raja et al. Trauma Informed Care in Medicine. Family Community Healthy. 2015. Vol 38: 216-226.

## Slide 47
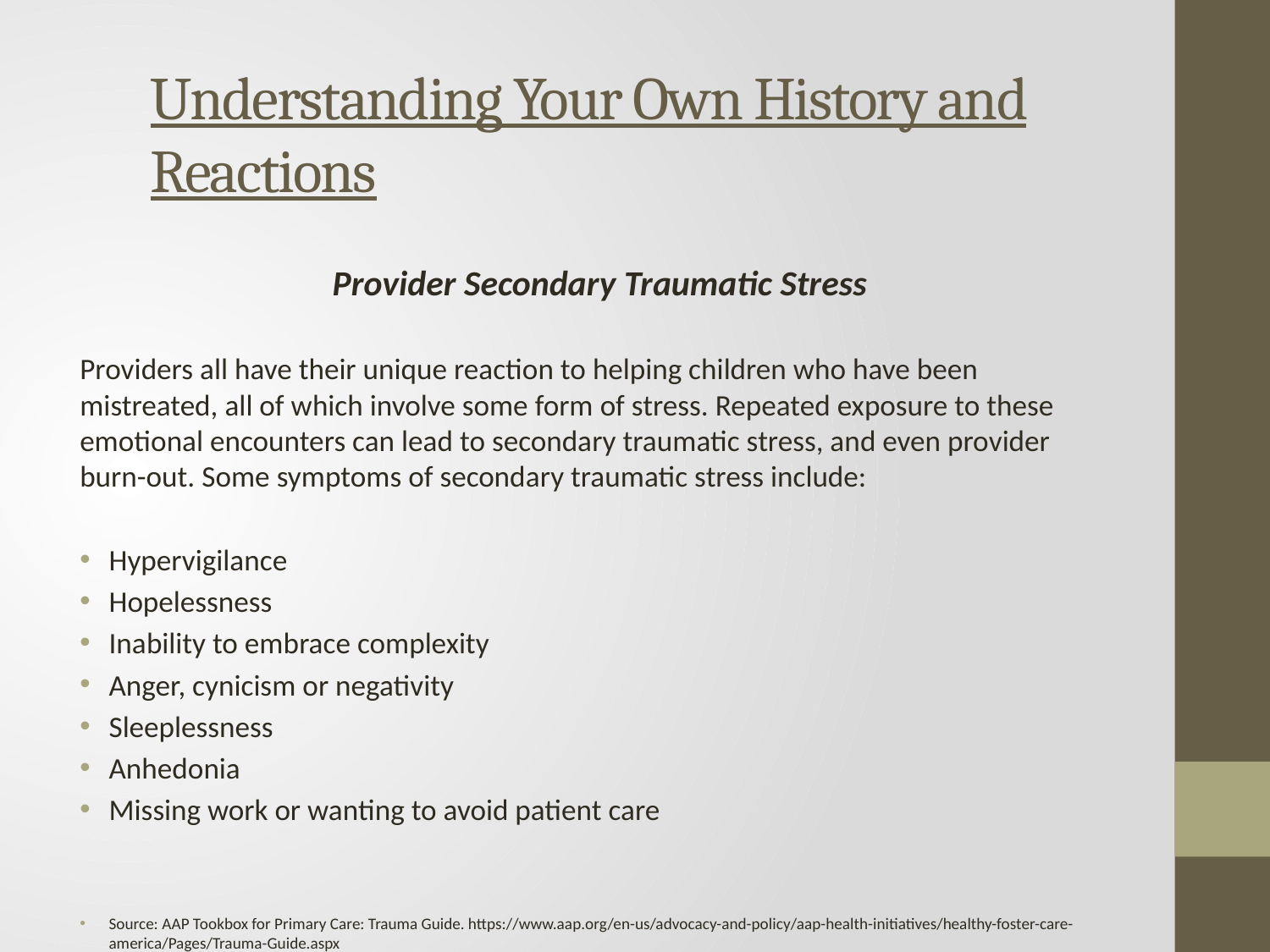

# Understanding Your Own History and Reactions
Provider Secondary Traumatic Stress
Providers all have their unique reaction to helping children who have been mistreated, all of which involve some form of stress. Repeated exposure to these emotional encounters can lead to secondary traumatic stress, and even provider burn-out. Some symptoms of secondary traumatic stress include:
Hypervigilance
Hopelessness
Inability to embrace complexity
Anger, cynicism or negativity
Sleeplessness
Anhedonia
Missing work or wanting to avoid patient care
Source: AAP Tookbox for Primary Care: Trauma Guide. https://www.aap.org/en-us/advocacy-and-policy/aap-health-initiatives/healthy-foster-care-america/Pages/Trauma-Guide.aspx

## Slide 48
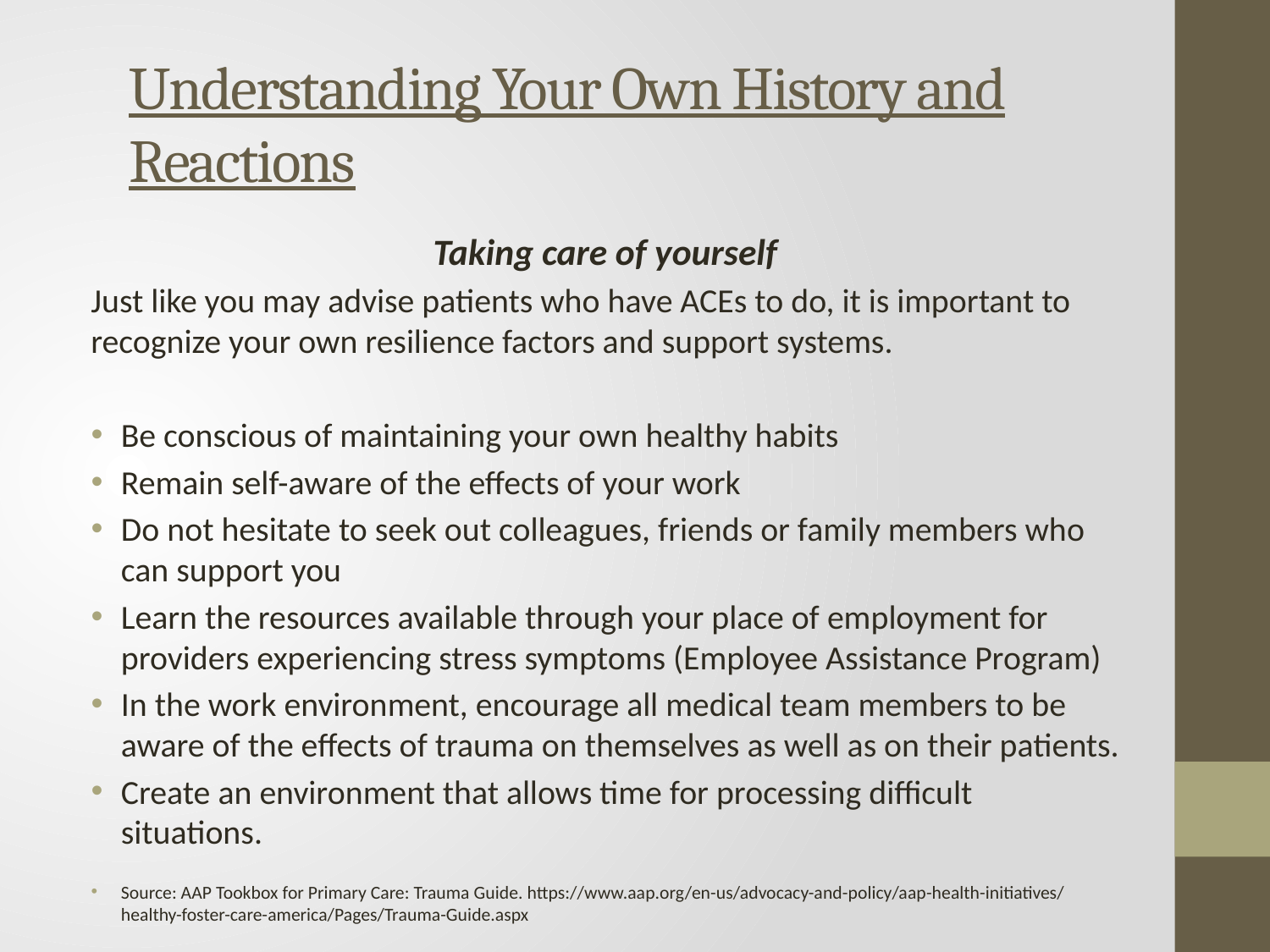

# Understanding Your Own History and Reactions
Taking care of yourself
Just like you may advise patients who have ACEs to do, it is important to recognize your own resilience factors and support systems.
Be conscious of maintaining your own healthy habits
Remain self-aware of the effects of your work
Do not hesitate to seek out colleagues, friends or family members who can support you
Learn the resources available through your place of employment for providers experiencing stress symptoms (Employee Assistance Program)
In the work environment, encourage all medical team members to be aware of the effects of trauma on themselves as well as on their patients.
Create an environment that allows time for processing difficult situations.
Source: AAP Tookbox for Primary Care: Trauma Guide. https://www.aap.org/en-us/advocacy-and-policy/aap-health-initiatives/healthy-foster-care-america/Pages/Trauma-Guide.aspx

## Slide 49
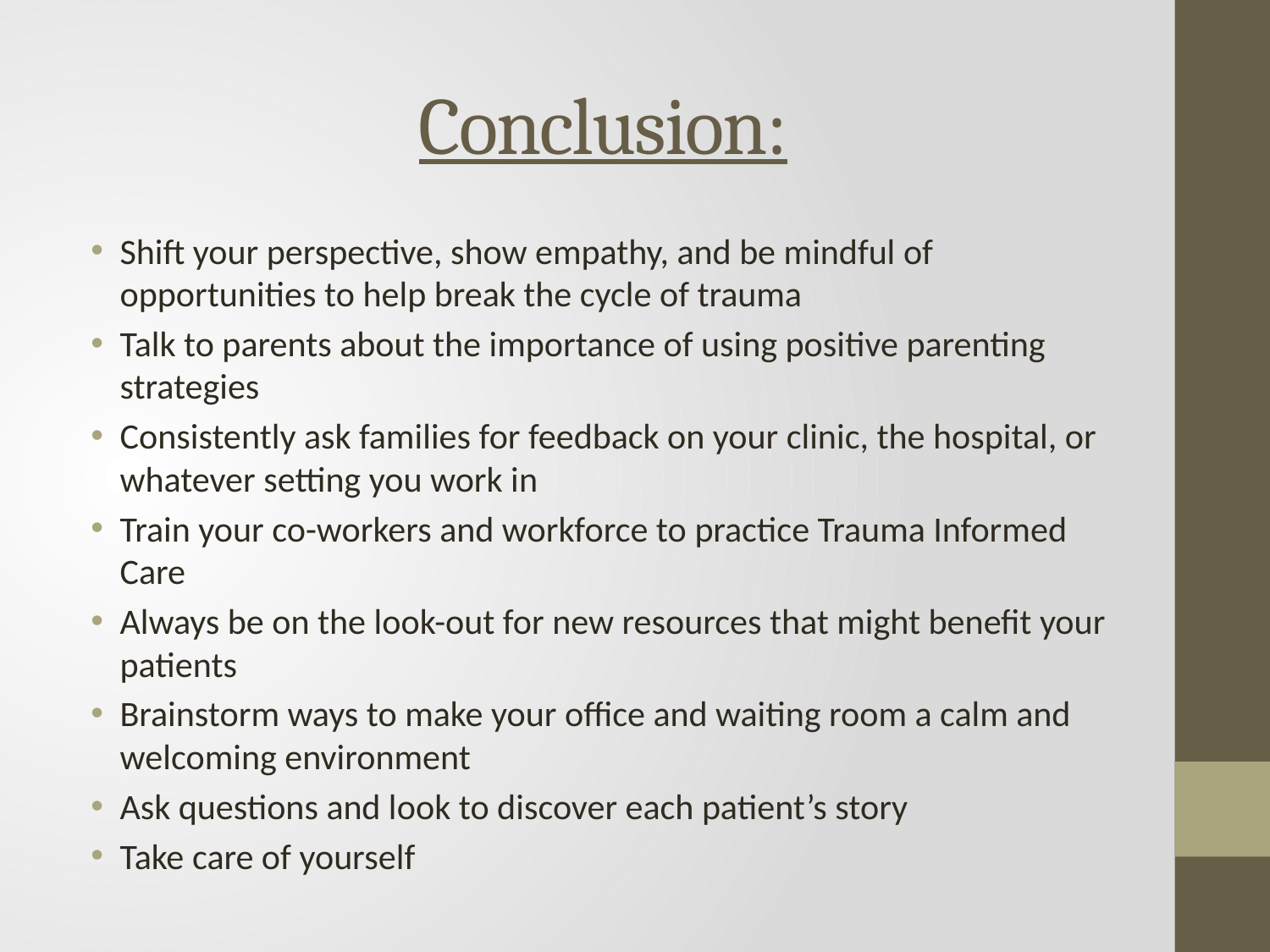

# Conclusion:
Shift your perspective, show empathy, and be mindful of opportunities to help break the cycle of trauma
Talk to parents about the importance of using positive parenting strategies
Consistently ask families for feedback on your clinic, the hospital, or whatever setting you work in
Train your co-workers and workforce to practice Trauma Informed Care
Always be on the look-out for new resources that might benefit your patients
Brainstorm ways to make your office and waiting room a calm and welcoming environment
Ask questions and look to discover each patient’s story
Take care of yourself

## Slide 50
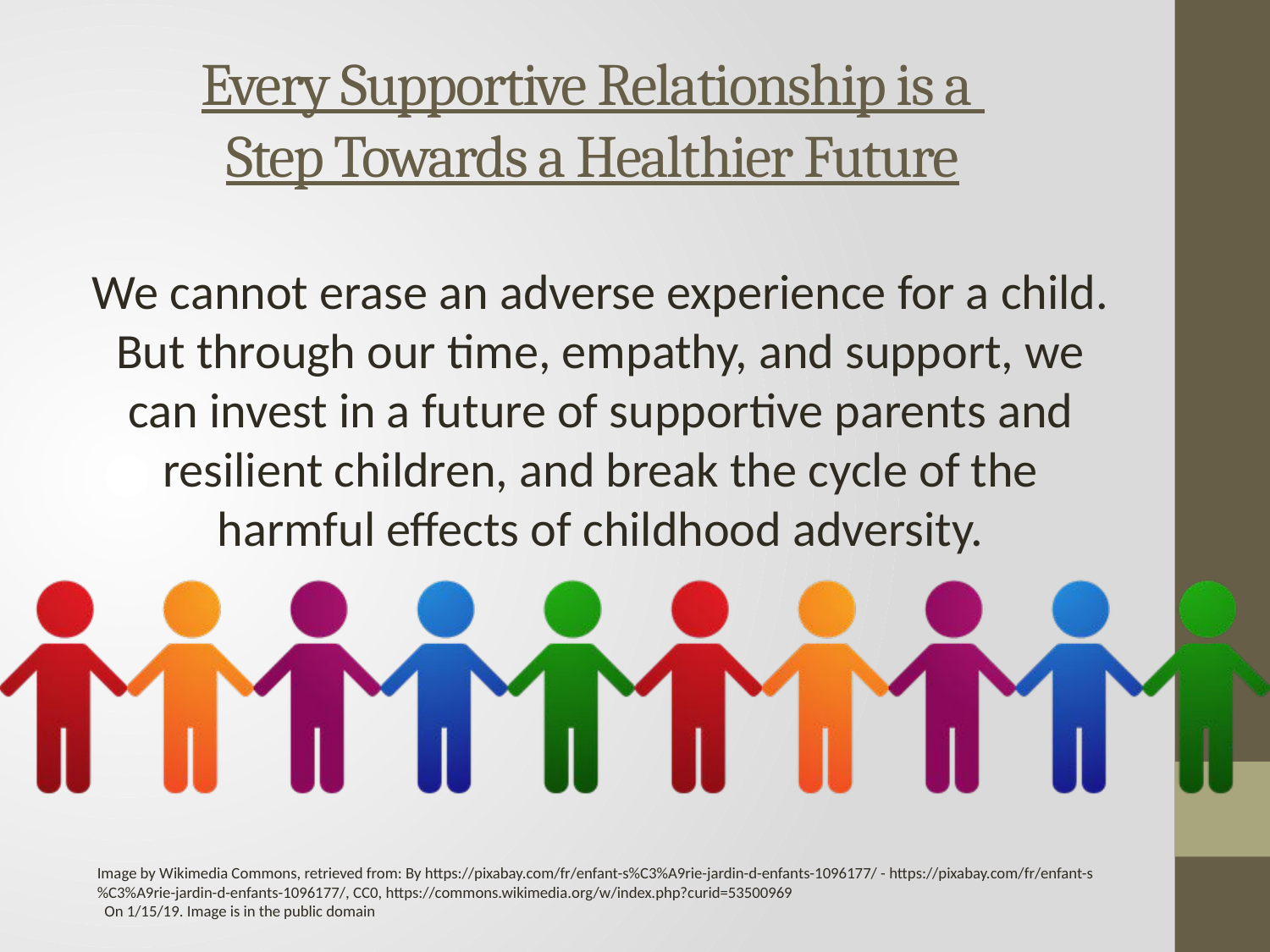

# Every Supportive Relationship is a Step Towards a Healthier Future
We cannot erase an adverse experience for a child. But through our time, empathy, and support, we can invest in a future of supportive parents and resilient children, and break the cycle of the harmful effects of childhood adversity.
Image by Wikimedia Commons, retrieved from: By https://pixabay.com/fr/enfant-s%C3%A9rie-jardin-d-enfants-1096177/ - https://pixabay.com/fr/enfant-s%C3%A9rie-jardin-d-enfants-1096177/, CC0, https://commons.wikimedia.org/w/index.php?curid=53500969
 On 1/15/19. Image is in the public domain

## Slide 51
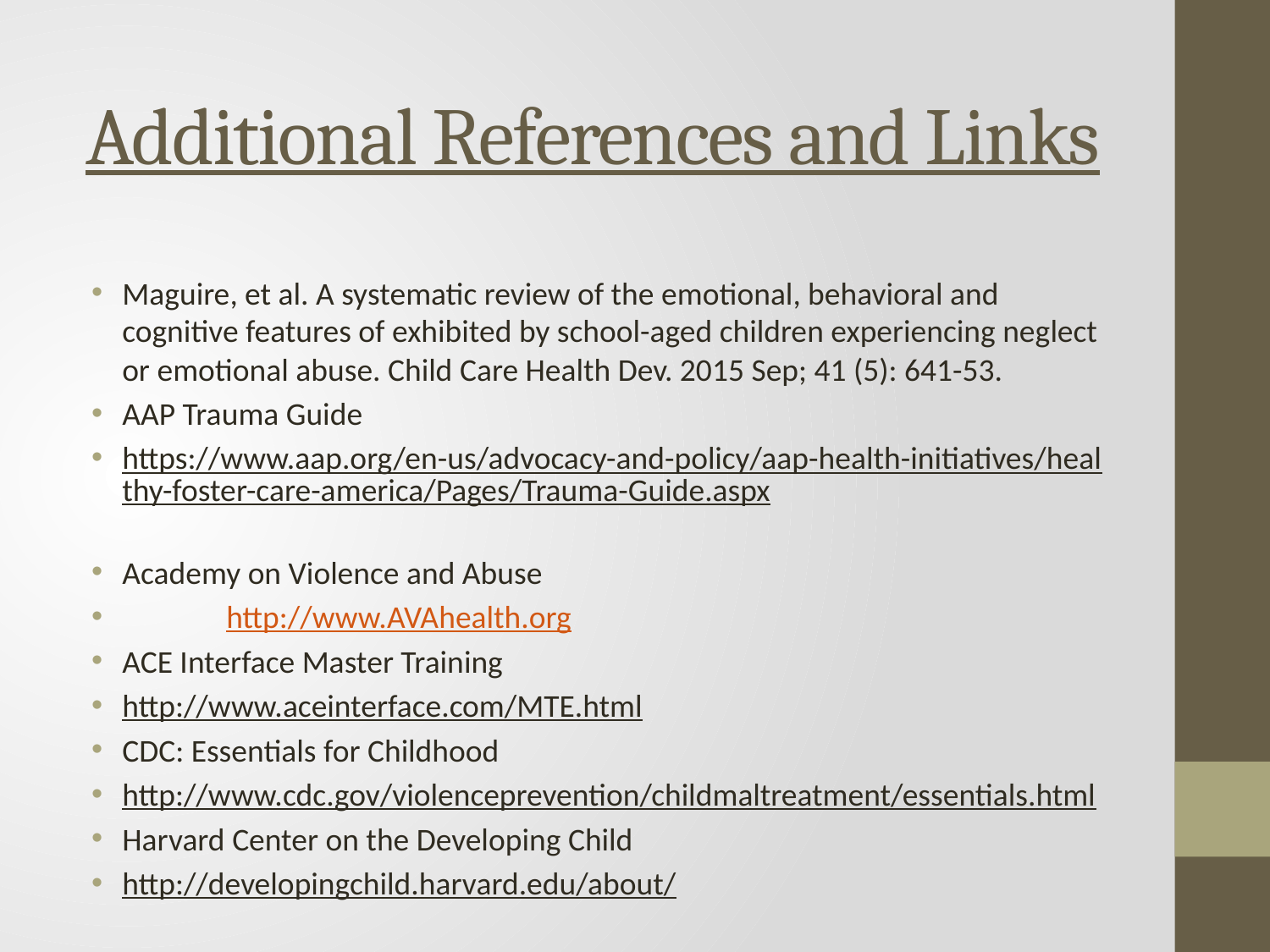

# Additional References and Links
Maguire, et al. A systematic review of the emotional, behavioral and cognitive features of exhibited by school-aged children experiencing neglect or emotional abuse. Child Care Health Dev. 2015 Sep; 41 (5): 641-53.
AAP Trauma Guide
https://www.aap.org/en-us/advocacy-and-policy/aap-health-initiatives/healthy-foster-care-america/Pages/Trauma-Guide.aspx
Academy on Violence and Abuse
	http://www.AVAhealth.org
ACE Interface Master Training
http://www.aceinterface.com/MTE.html
CDC: Essentials for Childhood
http://www.cdc.gov/violenceprevention/childmaltreatment/essentials.html
Harvard Center on the Developing Child
http://developingchild.harvard.edu/about/
