# Supplementary material for: Adverse Childhood Experiences and Trauma-Informed Care: An Online Module for Pediatricians
Source: MedEdPORTAL. 2019 Nov 1;15:10851. doi: 10.15766/mep_2374-8265.10851 (PMC6952282; doi:10.15766/mep_2374-8265.10851)
Supplement: Supplementary file 1 — A. ACEs PowerPoint.pptx B. ACEs Premodule Survey.docx C. ACEs Postmodule Survey.docx [file mep-15-10851-s001.zip › B. ACEs Premodule Survey.docx]

**ACEs Pre Module Survey**

1. **Please create your unique ID number: using all caps, type the first 2 letters of your mother's first name, the month of your birth, and the last 2 digits of your social security number: For example, your mother's name is Sarah, you were born in April, and your SSN is ***-**-**67, your ID would be: SA0467**

*** 2. How confident do you feel in your knowledge of Adverse Childhood Experiences, Trauma Informed Care, Toxic Stress and Resiliency?**

|  | **Not Confident** | **Slightly Confident** | **Neutral** | **Somewhat Confident** | **Very Confident** |
| --- | --- | --- | --- | --- | --- |
| **Adverse Childhood Experiences** |  Not Confident | Slightly Confident |  Neutral |  Somewhat Confident |  Very Confident |
| **Trauma Informed Care** | Not Confident |  Slightly Confident |  Neutral |  Somewhat Confident |  Very Confident |
| **Toxic Stress** |  Not Confident | Slightly Confident |  Neutral | Somewhat Confident |  Very Confident |
| **Resiliency** |  Not Confident |  Slightly Confident |  Neutral |  Somewhat Confident |  Very Confident |

*** 3. How confident do you feel discussing Adverse Childhood Experiences, Trauma Informed Care, Toxic stress, and resiliency with patients and families?**

|  | **Not Confident** | **Slightly Confident** | **Neutral** | **Somewhat Confident** | **Very Confident** |
| --- | --- | --- | --- | --- | --- |
| **Adverse Childhood Experiences** |  Not Confident |  Slightly Confident |  Neutral |  Somewhat Confident |  Very Confident |
| **Trauma Informed Care** |  Not Confident |  Slightly Confident |  Neutral |  Somewhat Confident |  Very Confident |
| **Toxic Stress** |  Not Confident |  Slightly Confident |  Neutral |  Somewhat Confident |  Very Confident |
| **Resiliency** |  Not Confident |  Slightly Confident |  Neutral |  Somewhat Confident |  Very Confident |

*** 4. How often do you discuss Adverse Childhood Experiences, Trauma-Informed Care, Toxic Stress, and Resiliency with families in your continuity clinic?**

|  | **No visits** | **Rare visits** | **Some visits** | **Most visits** | **All visits** |
| --- | --- | --- | --- | --- | --- |
| **Adverse Childhood Experiences** |  No visits |  Rare visits |  Some visits | Most visits |  All visits |
| **Trauma-Informed Care** |  No visits |  Rare visits |  Some visits |  Most visits |  All visits |
| **Toxic Stress** |  No visits |  Rare visits | Some visits |  Most visits |  All visits |
| **Resiliency** |  No visits |  Rare visits |  Some visits |  Most visits |  All visits |

*** 5. How frequently does your knowledge of your patient's Adverse Childhood Experiences, trauma-Informed care, toxic stress, and resiliency affect your clinic visit and clinical decision making for that patient?**

|  | **Never** | **Rarely** | **Occasionally** | **Usually** | **Always** |
| --- | --- | --- | --- | --- | --- |
| **Adverse Childhood Experiences** |  Never |  Rarely |  Occasionally |  Usually |  Always |
| **Trauma-Informed Care** |  Never |  Rarely |  Occasionally |  Usually |  Always |
| **Toxic Stress** |  Never |  Rarely |  Occasionally |  Usually |  Always |
| **Resiliency** |  Never |  Rarely |  Occasionally |  Usually |  Always |

*** 6. How important is it to discuss or address Adverse Childhood Experiences, Trauma Informed Care, Toxic Stress, and resiliency with families?**

|  | **Not Important** | **Slightly Important** | **Neutral** | **Somewhat Important** | **Very Important** |
| --- | --- | --- | --- | --- | --- |
| **Adverse Childhood Experiences** |  Not Important |  Slightly Important |  Neutral |  Somewhat Important |  Very Important |
| **Trauma Informed Care** |  Not Important |  Slightly Important |  Neutral |  Somewhat Important |  Very Important |
| **Toxic Stress** |  Not Important |  Slightly Important |  Neutral |  Somewhat Important |  Very Important |
| **Resiliency** |  Not Important |  Slightly Important |  Neutral |  Somewhat Important |  Very Important |

*** 7. What type of continuity clinic do you participate in?**

Federally Qualified Health Center

Suburban Private Practice- <50% Medicaid patients

Suburban Private Practice- >50% Medicaid patients

*** 8. How long do you spend in a typical visit at your continuity clinic?**

<10 minutes

10-20 minutes

20-30 minutes

Greater than 30 minutes
